# Dual Amino Acid Swap in MUC7-Derived Peptide Enhances Resistance and Modulates Zn(II) and Cu(II) Complex Stability, Secondary Structure and Antimicrobial Activity

**DOI:** 10.1021/acs.inorgchem.5c05849

**Published:** 2026-03-04

**Authors:** Klaudia Szarszoń, Jan Kachnowicz, Tomasz Janek, Alicia Domínguez-Martin, Aneta Jezierska, Joanna Wątły

**Affiliations:** † Faculty of Chemistry, 49572 University of Wrocław, F. Joliot-Curie 14, 50-383 Wrocław, Poland; ‡ Department of Biotechnology and Food Microbiology, 56641 Wrocław University of Environmental and Life Sciences, Chełmońskiego 37, 51-630 Wrocław, Poland; § Department of Inorganic Chemistry, Faculty of Pharmacy, 16741 University of Granada, E-18071 Granada, Spain

## Abstract

Metal ion complexes with antimicrobial peptides are emerging as promising therapeutic agents, but their efficacy is often limited by rapid proteolytic degradation and poorly understood metal-driven mechanisms. Here, we stabilize a sequence derived from mucin 7 by introducing two d-amino acids at the site most susceptible to proteolytic cleavage and show that this subtle modification rewires its copper­(II) and zinc­(II) coordination chemistry and antimicrobial activity. The coordination, structure and activity of the modified peptide (peptidomimetic) with Cu­(II) and Zn­(II) were probed by potentiometry, mass spectrometry, multiple spectroscopies and quantum-chemical calculations, together with antimicrobial and antibiofilm assays. The peptidomimetic is resistant to enzymatic cleavage and forms thermodynamically more stable Cu­(II) complexes with {3N_im_} donor sets, in contrast to the native peptide which favors {2N_im_} binding, while Zn­(II) speciation is only modestly affected. Metal loading together with the d-amino-acid-induced increase in conformational flexibility converts the otherwise inactive peptide into a selective, metal-dependent antimicrobial peptide (AMP) that targets *Streptococcus mutans* and *Streptococcus sanguinis*, inhibits their biofilm formation at subinhibitory minimum inhibitory concentration (sub-MIC) levels and remains nontoxic to human fibroblasts. These results demonstrate how fine-tuning histidine-rich coordination environments by minimal stereochemical editing can be used to design stable, Cu­(II)- and Zn­(II)-based antimicrobial peptidomimetics with distinct, metal-specific mechanisms of action.

## Graphical abstract

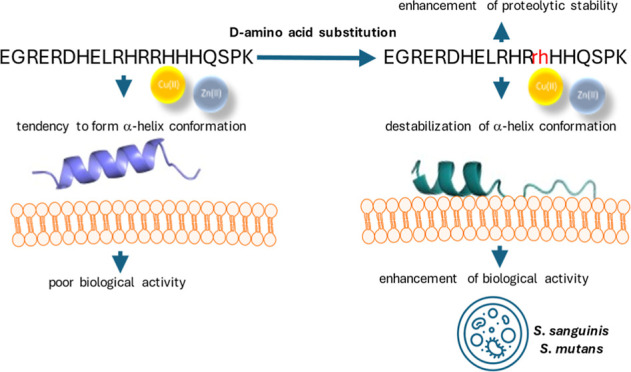

## Introduction

Antimicrobial peptides (AMPs) are small molecules that play a key role in the host’s innate immune system.^1,2^ They serve as a primary defense mechanism against various pathogens, including bacteria, viruses, fungi, and parasites.^3,4^ They typically act by disrupting the integrity of microbial membranes,^5,6^ resulting in cell death, and by targeting intracellular components to inhibit the synthesis of nucleic acids, enzymes, and other essential proteins.^7^ Additionally, they can utilize less common methods, such as molecular electroporation and sinking raft mechanisms.^8^ Due to their broad-spectrum antimicrobial activity and their ability to evade resistance mechanisms commonly seen in conventional antibiotics, AMPs are emerging as promising therapeutic agents for treating infectious diseases and other conditions.^9^ Despite their considerable therapeutic potential, peptides exhibit significant limitations, particularly their low stability, which makes them highly susceptible to degradation by proteases.^10^ Proteases (also referred to as proteolytic enzymes, peptidases, or proteinases) are enzymes that degrade proteins and rapidly cleave peptides, thereby reducing their half-life and frequently diminishing their efficacy as therapeutics.^10,11^ Their mode of action involves hydrolyzing peptide bonds within proteins, yielding smaller peptide fragments or individual amino acids. Most proteolytic enzymes specifically target α-peptide bonds between naturally occurring amino acids.^12^

Human saliva consists of a diverse mixture of bioactive proteins and peptides,^13^ including those with antimicrobial activity such as histatins, defensins, and mucins.^14,15^ It also contains a small yet significant amount of proteolytic enzymes. Many bacteria secrete a variety of proteases, ranging from broad-spectrum enzymes that degrade key proteins involved in innate immunity to highly specific proteases with targeted functions.^16^ Proteolytic enzymes from periodontal bacteria contribute to periodontal diseases by affecting IgG and IgA levels in saliva,^17^ for instance it has been observed that these bacteria produce ‘trypsin-like’ proteinases.^18^ Many of histidine-rich peptides found in saliva arise as products of enzyme-mediated proteolysis, indicating the presence of a proteolytic degradation pathway in the oral environment.^19,20^ The most well-known examples are histatins.,^19,21–23^ and peptides derived from N-terminal fragment of mucin 7.^24^

Natural enzymatic degradation often leads to the formation of peptides with enhanced antimicrobial activity compared to the native peptide^25^ or the parent protein, and these fragments frequently exhibit distinct mechanisms of action. However, eventually, proteolytic cleavage results in a complete loss of activity due to extensive peptide fragmentation.^26^ To avoid the impact of proteases, antimicrobial peptides can be modified to create antimicrobial peptidomimetics, which possess a high potential for combating pathogens. These analogues mimic key physicochemical features of native peptides, such as amphipathicity and positive charge, while maintaining resistance to proteolytic degradation.^27–29^ One type of modification that can be used is the incorporation of unusual amino acids into the native peptide sequence, such as d-, β- and γ-amino acids.^30–32^ In our previous study, we examined a peptide^33^ derived from the N-terminal fragment of the salivary glycoprotein mucin 7 (MUC7/MG2), EGRERDHELRHRRHHHQSPK, along with its hydrolytic fragments: EGRERDHELRHRR and HHHQSPK (Figure 1) in the presence of Cu­(II) and Zn­(II) ions. The results showed that Zn­(II) coordination only slightly enhanced the antimicrobial activity of the 20-mer peptide, and only against *Streptococcus sanguinis*, with a minimum inhibitory concentration (MIC) of 500 μg/mL at pH 7.4. More intriguing was the observation that the Cu­(II) and Zn­(II) complexes formed with the shorter peptides (resulting from proteolytic degradation) exhibited superior antimicrobial activity compared to the native peptide, with the best MIC values for the Zn­(II)-HHHQSPK system at pH 5.4 (125 μg/mL).^33^

**Figure 1:**
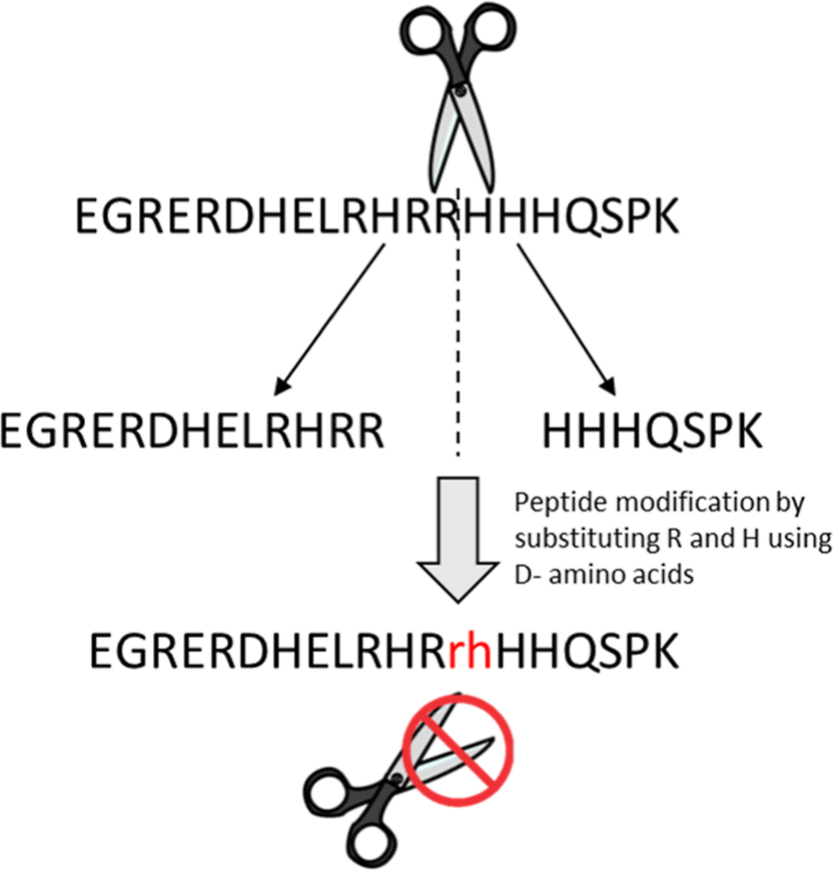
Amino acid sequence of the native peptide (EGR L-aa, top), its naturally occurring hydrolytic fragments (middle), and the peptidomimetic analogue used in this work containing two d-amino acids (highlighted in red, EGR 2D-aa, bottom).

Building on our previous studies of the native peptide (EGRERDHELRHRRHHHQSPK, EGR L-aa),^33^ in this work we investigated its peptidomimetic analogue (EGRERDHELRHRrhHHQSPK, EGR 2D-aa), generated by substituting two l-amino acids (Arg and His) at a proteolytically sensitive cleavage site with their d-enantiomers. Such stereochemical modification has been previously used, herein concomitant site-specific modification eager not only to enhance proteolytic stability but also alter donor accessibility, which in turn could influence coordination modes and metal ion selectivity. The experimental investigations, employing a range of physicochemical techniques (potentiometry, mass spectrometry, and spectroscopic methods including ultraviolet–visible spectroscopy (UV–vis), circular dichroism (CD), and electron paramagnetic resonance (EPR)), were complemented by quantum-chemical calculations to gain insight into the binding mode of Cu­(II) and Zn­(II) ions with the studied peptidomimetic. Enzymatic stability of the peptidomimetic, as well as biological activity assays for the ligand and its complexes at two different pH values (5.4 and 7.4), typical of human saliva, were also performed. The objectives of the study were to determine whether the modified peptide would: (i) exhibit greater proteolytic stability, (ii) differ in its coordination modes with Cu­(II) and Zn­(II) ions compared to the native peptide, (iii) display altered thermodynamic stability of the resulting metal complexes, (iv) differ in its propensity to form secondary structures, and finally (v) display greater antimicrobial activity and, if so, to elucidate the underlying molecular and coordination-chemical basis of this effect.

## Experimental Section

### Materials

The peptidomimetic EGRERDHELRHRrhHHQSPK (EGR 2D-aa, in which lower-case letters denote d-amino acids), an analogue of the MUC7 fragment, was purchased from KareBay Biochem (certified purity: 98%) and used as received (Figure S1). Its purity was checked using potentiometry and mass spectrometry. Cu­(ClO_4_)_2_·6H_2_O and Zn­(ClO_4_)_2_·6H_2_O were extra-pure products (Sigma-Aldrich, St. Louis, MO). The concentrations of stock solutions were determined by using inductively coupled plasma spectrometry (ICP-OES). The carbonate-free stock solution of 0.1 M NaOH (EuroChem, Zug, Switzerland) was standardized potentiometrically with potassium hydrogen phthalate (Sigma-Aldrich, St. Louis, MO). All samples were prepared using ultrapure water of the first degree of purity according to the ISO 3696:1999 standard (produced by Hydrolab Ultra UV system). The measurements were recorded in 4 mM HClO_4_ with ionic strength 100 mM NaClO_4_. All samples were weighted using an analytical scale, Sartorius R200D.

### Mass Spectrometric Measurements

To record high-resolution mass spectra to confirm the purity of the ligand and the stoichiometry of metal–ligand complexes, a Bruker Compact QTOF (Bruker Daltonics, Bremen, Germany) spectrometer equipped with an electrospray ionization source (ESI-MS) with an ion funnel was used. The mass spectrometer was operated in the positive ion mode with the following parameters: scan range *m*/*z* 50–3000; dry gas nitrogen; temperature 180 °C; capillary voltage 4000 V; ion energy 5 eV. The Cu­(II) and Zn­(II) complexes [(metal/ligand stoichiometry of 1:1) [ligand]_tot_ = 0.1 mM] were prepared in a 50:50 (v/v) MeOH/H_2_O mixture. The samples were infused at a flow rate of 3 μL/min. The instrument was calibrated externally with the Low Concentration Tuning Mix ESI-ToF (Agilent Technologies, Waldbronn, Germany). The data were processed using the Bruker Compass DataAnalysis 4.2 program (Bruker Daltonics, Bremen, Germany).

### Trypsin Digestion Experiment

An experiment was performed in which two types of samples were examined: (i) a sample containing the peptide/peptidomimetic (1 mg/mL), trypsin solutions, and an ammonium carbonate buffer at pH 8.2, and (ii) a control sample containing trypsin solutions and an ammonium carbonate buffer at pH 8.2. The solutions were incubated for 24 h in a water bath at 37 °C, then a 5% trifuoloacetic acid (TFA) solution was added to stop the reaction—the pH was lowered to approximately 3.5. Then the solutions were transferred to Vivaspin 500 concentrators (PES Membrane 5000 Da) and centrifuged. For the filtered solutions, mass spectrometry analysis was performed using JEOL JMS-S3000 SpiralTOF-plus Ultra-High Mass Resolution Matrix-assisted Laser Desorption/Ionization (MALDI-TOF) mass spectrometer. The matrix compound used to produce the matrix solution was sinapic acid. Sinapic acid (10 mg) was dissolved in 1 mL of 50/50 acetonitrile/water solvent containing 0.1% TFA. The sample and matrix mix were mixed 1:1 by volume and spotted onto a 96-well stainless steel plate and allowed to air-dry. The results were analyzed using the msTornado Analysis (version 2.0; 2.0.11.1).

### Potentiometric Measurements

Potentiometric measurements were performed under an argon atmosphere at a constant temperature of 298 K using a Metrohm Titrando 905 titrator equipped with a Mettler Toledo InLab Semi-Microcombined pH electrode. Stability constants for the proton and metal (Cu­(II) and Zn­(II)) complexes were determined from titration curves performed over the pH range of 2.0–12.0 in a total volume of 2.7 mL. The glass cell was equipped with a magnetic stirring system, a microburet delivery tube, and an inlet–outlet tube for argon. The potentiometric titrations were carried out in 4 mM HClO_4_ with an ionic strength (*I*) of 100 mM NaClO_4_. The solutions were titrated with 100 mM carbonate-free NaOH (Fluka). The electrodes were calibrated daily for hydrogen ion concentration by titrating HClO_4_ with NaOH using a total volume of 3.0 mL under the same experimental conditions as above. The purity and the exact concentrations of the ligand solutions were determined using the Gran method.^34^ The ligand concentration was 0.4 mM, with a Cu­(II) and Zn­(II) to ligand ratio of 0.9:1. Stability constant calculations were conducted using the HYPERQUAD 2006 program.^35^ Standard deviations were computed by HYPERQUAD 2006 and were referred to random errors exclusively. Hydrolysis constants for Zn­(II) ions were taken from the literature.^36^ The speciation and competition diagrams were computed using the HYSS program^37^ and visualized in the OriginPro 2016 program.

### Spectroscopic Studies

The UV–vis spectra were recorded on a Jasco V-750 spectrophotometer in the 800–200 nm range using a quartz cuvette with a 1 cm path length. The CD spectra were obtained with a Jasco J-1500 CD spectropolarimeter within the 800–180 nm range using different path lengths (1 cm and 0.2 mm). The concentrations of solutions utilized for UV–vis and CD spectroscopic analyses were similar to those employed in the potentiometric experiments. The pH of the samples was adjusted by adding appropriate amounts of concentrated NaOH and HClO_4_ solutions, if needed. The CD spectra were obtained using the Jasco Spectra Analysis Software. Characterization of the species generated in solution involved comparing the observed wavelength of maximum absorption in the UV–vis range at specific pH values with λ_max_ values reported in the literature. The EPR spectra were acquired at liquid nitrogen temperature utilizing a Bruker ELEXSYS E500 CW-EPR spectrometer operating at a band frequency of 9.5 GHz. The ligand under investigation was dissolved in aqueous solutions of HClO_4_ acid at *I* = 100 mM (NaClO_4_), with the addition of ethylene glycol (25%) as a cryoprotectant. The concentration of copper ions was 1 mM, and the metal/ligand ratio was 0.9:1. The obtained EPR spectra were analyzed to determine the EPR parameters, which characterize the molecular and electron structure of the copper complexes, employing a simulation method. This involved identifying the best fit between the theoretical and experimental spectra. The theoretical (simulated) EPR spectra were generated using the WinEPR SimFonia software, version 1.2 (Bruker), using the appropriately selected spin Hamiltonian EPR parameters for *S* = 1/2, including the diagonal components of the tensors: *g* (*g*_∥_ = *g*_*z*_, *g*_⊥_ = *g*_*x*_ = *g*_*y*_) and *A*—interaction of an unpaired electron of copper­(II) with a nuclear spin of copper, *I*(^63,65^Cu) = 3/2, (*A*_∥_ = *A*_*z*_, *A*_⊥_ = *A*_*x*_ = *A*_*y*_). All spectroscopic measurements were recorded in the pH range 3.0–12.0. OrginPro 2016 was used to process and visualize the obtained spectra.

### Computational Methodology

The EGR 2D-aa peptidomimetic (a model with amino acid residues numbered according to their position is shown in Figure 2) was used in theoretical investigations with both Cu­(II) and Zn­(II) ions at two pH values (5.4 and 7.4). In the prepared model, two l-amino acids (Arg-13 and His-14) were replaced by their d-isomers. XYZ coordinate files of short models discussed are given as the last part of the Supporting Information file.

**Figure 2:**
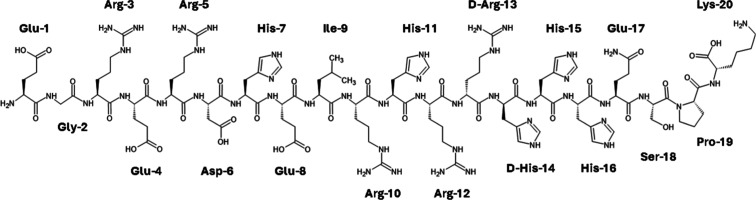
Peptidomimetic ligand EGR 2D-aa (EGRERDHELRHRrhHHQSPK) shown with the amino acid numbering scheme used in the Cu­(II) and Zn­(II) studies. Note that this scheme does not imply structural changes as a function of pH.

To prepare the peptidomimetic ligand for the quantum-chemical studies, the Modeler 10.5 ^38^ program was used. Based on the obtained results, the most probable secondary structure was selected for further theoretical simulations. For each possible combination of donor groups, molecular models (long models) were constructed on the basis of potentiometric and spectroscopic studies using the Avogadro^39^ and Molden^40,41^ programs. To compare the energies of the possible complexes, all structures were optimized using the GFN2-xTB^42^ semiempirical method with the ALPB^43^ solvation model to simulate the aqueous environment in the xTB 6.7.1.^44^ The Single Point Hessian (SPH)^45^ was employed to calculate harmonic frequencies and thermodynamic corrections in order to obtain free energies. Models that experienced convergence problems were optimized using the same approach, but with the GFN1-xTB method.^46^ Based on these results, three models with the lowest free energies at pH 5.4 and 7.4 with Cu­(II)/Zn­(II) ions were selected for further studies. In total, 12 models were obtained. Subsequently, short models were constructed by deleting nonbinding amino acid residues on both sides, leaving only fragments starting with His-7 and ending with His-16 (see Figure 3). The C-terminal group was capped with an amine to form an amide group, while the N-terminal group was capped with COCH_3_. In cases where the N-terminal amine group contributed to metal ion binding, short models were constructed by deleting the four nonbinding amino acids and capping only at the C-terminal end. Short models were further optimized using more accurate Density Functional Theory (DFT)^47,48^ composite method r^2^SCAN-3c,^49^ together with the CPCM^50^ solvation model (water as a solvent) in the ORCA 6.0.1.^51–53^ suite of programs. All graphical visualizations were prepared using the VMD 1.9.3.^54^

**Figure 3:**
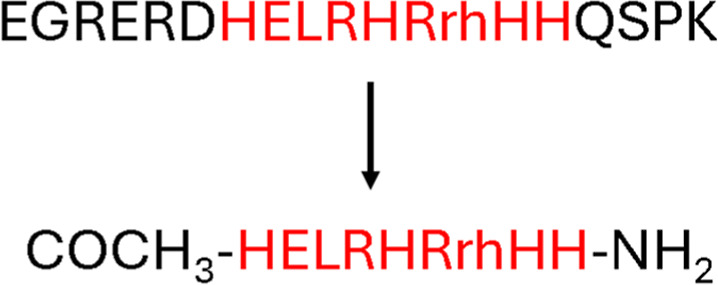
Schematic representation of short models construction.

The peptidomimetic ligand EGR 2D-aa models with Cu­(II) and Zn­(II) ions at two pH environments (pH 5.4 and 7.4) were used in quantum-chemical simulations as a starting point to estimate possible stable conformations. The obtained so-called long models gave us a general overview of the structural changes when the metal ion was introduced to the system, exhibiting high flexibility, which forced us to decrease the number of amino acids to better describe the binding of metal ions. The set of so-called short models was obtained. It is worth mentioning that during the energy minimization, some of the models proved to be unstable. Another important issue associated with the models’ construction is the presence of water molecules in the vicinity of the metal ions. In the present study, we considered models containing the zinc ion with two arbitrarily located water molecules. Experimental data indicate that water molecules are highly labile, making it difficult to determine their exact number and positions.

### ROS Detection

#### Ascorbate Consumption Experiments

UV–vis spectra were recorded on a JASCO V-750 spectrophotometer at 25 °C. The intensity of the ascorbate (Asc) absorption band at λ_max_ = 265 nm was monitored as a function of time in a 50 mM phosphate buffer solution at pH 5.4. The final concentrations of the reagents in the cuvette were as follows: 10 μM (peptide), 8 μM (Cu­(II)), and 100 μM (Asc). The absorbance was measured every 10 s for 40 min after the addition of the peptide/Cu­(II)/complex to Asc solution in buffer (control measurement was measured every 10 s for 10 min) using a quartz cuvette with an optical path of 1 cm. To guarantee the reliability of the results, each experiment was conducted three times.

#### HO^•^ Scavenging Monitoring

Coumarin-3-carboxylic acid (CCA) was employed as a fluorescence probe to detect hydroxyl radicals (HO^•^). This detection occurs because HO^•^ reacts with CCA to produce 7-hydroxycoumarin-3-carboxylic acid (7-OH–CCA), which exhibits fluorescence at 452 nm when excited at 395 nm. The fluorescence intensity is directly proportional to the quantity of 7-OH–CCA molecules formed, which correlates with the amount of HO^•^ radicals generated. The samples were prepared in 50 mM phosphate buffer at pH 5.4. The final concentrations of the reagents in the cuvette were as follows: 60 μM (peptidomimetic), 50 μM (Cu­(II)), 200 μM (CCA), and 200 μM (Asc). Ascorbate was added last, and the measurement started immediately. Fluorescence experiments were performed on an RF-6000 spectrofluorometer (Shimadzu). The fluorescence was measured every 30 s for 1 h.

#### In Vitro Antimicrobial Activity of Peptidomimetic and Peptidomimetic-Metal Ion Systems

The antimicrobial properties of the peptidomimetic and its complexes were tested against human pathogenic strains. The microbial strains used in this study were sourced from the American Type Culture Collection (ATCC, USA) and the Polish Collection of Microorganisms (PCM, Poland). The antimicrobial efficacy of the ligand and its metal complexes was evaluated against the following microorganisms: Gram-negative bacteria *Escherichia coli* ATCC 25922 and *Pseudomonas aeruginosa* ATCC 15442, Gram-positive bacteria *Enterococcus faecalis* ATCC 29212, *Staphylococcus aureus* ATCC 25923, *Streptococcus mutans* PCM 2502, and *Streptococcus sanguinis* PCM 2335, as well as the yeast *Candida albicans* SC5314.^55^ These strains were preserved at −80 °C as glycerol stocks in the Department of Biotechnology and Food Microbiology at the Wrocław University of Environmental and Life Sciences, Wrocław, Poland. *E. coli* ATCC 25922, *P. aeruginosa* ATCC 15442, *E. faecalis* ATCC 29212, and *S. aureus* ATCC 25923 were cultivated at 37 °C in Mueller–Hinton broth (MHB; Merck Millipore, Darmstadt, Germany). *S. mutans* PCM 2502 and *S. sanguinis* PCM 2335 were grown in Brain Heart Infusion (BHI) broth (Merck Millipore, Darmstadt, Germany) and incubated anaerobically (85% N_2_, 10% H_2_, and 5% CO_2_) at 37 °C overnight. *C. albicans* SC5314 was cultured aerobically at 37 °C in Yeast Peptone Dextrose (YPD) broth (A&A Biotechnology, Gdańsk, Poland).

The minimum inhibitory concentration (MIC) values of the peptidomimetic, its metal complexes, and metal ions were determined using the broth microdilution technique. Briefly, 2-fold serial dilutions of each peptidomimetic/complex were prepared in Mueller–Hinton broth (MHB), Brain Heart Infusion (BHI) broth, and Yeast Peptone Dextrose (YPD) broth, each buffered with 10 mM 4-morpholineethanesulfonic acid (MES) buffer, pH 5.4 (Merck Millipore, Darmstadt, Germany), or 10 mM 4-(2-hydroxyethyl)­piperazine-1-ethanesulfonic acid (HEPES) buffer, pH 7.4 (Merck Millipore, Darmstadt, Germany). A volume of 100 μL was added to each well of 96-well flat-bottom microtiter plates (Sarstedt, Nümbrecht, Germany), with final concentrations of the peptide/complex ranging from 7.8 to 500 μg/mL. Similarly, the MIC assay for copper (Cu) and zinc (Zn) ions was performed with concentrations ranging from 0.3 to 38 μg/mL, corresponding to the metal ion concentrations present in the respective complexes. A 1 μL aliquot of a 24 h microbial culture was inoculated into each well of a microtiter plate, ensuring a final cell density of 5 × 10^7^ CFU/mL. The microplates were incubated at 37 °C for 24 h for *E. coli* ATCC 25922, *P. aeruginosa* ATCC 15422, *E. faecalis* ATCC 29212, *S. aureus* ATCC 25923, and *C. albicans* SC5314. The two oral bacterial strains, *S. mutans* PCM 2502 and *S. sanguinis* PCM 2335, were incubated anaerobically (85% N_2_, 10% H_2_, and 5% CO_2_) at 37 °C, with optical density at 600 nm (OD_600_) measured after 72 h using a microplate reader (Spark, Tecan Trading AG, Switzerland).

The MIC end point was defined as the lowest concentration with complete (100%) growth inhibition. All the measurements were performed in three independent experiments.

#### Biofilm Quantification

Biofilm inhibition was assessed in 96-well flat-bottom polystyrene microplates (Sarstedt, Nümbrecht, Germany) against the bacterial strains for which peptidomimetic and its complexes showed antimicrobial activity, namely *S. sanguinis* and *S. mutans*. Peptidomimetic and its metal complexes were prepared at 0.5 × MIC and 0.75 × MIC in BHI broth, and bacterial inoculum was added to each well to achieve a final concentration of 5 × 10^5^ CFU/mL, with a total volume of 100 μL per well. The plates were incubated at 37 °C for 72 h.

To assess biofilm formation, the supernatant was carefully removed after incubation, and the wells were washed twice with 100 μL of 0.9% NaCl solution to remove planktonic cells. The remaining biofilm was fixed with 100 μL of 100% methanol for 15 min, stained with 100 μL of 1% (v/v) crystal violet for 5 min and washed three times with saline solution. Finally, 150 μL of isopropanol-0.04 N HCl and 50 μL of 0.25% sodium dodecyl sulfate (SDS) per well were added to solubilize the stain. The samples were mixed gently, and absorbance was measured at 590 nm using a microplate reader (Spark, Tecan Trading AG, Switzerland).

#### Cytotoxicity Assay

Normal Human Dermal Fibroblasts (NHDF) (Lonza, Basel, Switzerland) were cultured in α-Minimum Essential Medium (α-MEM, Institute of Immunology and Experimental Therapy (IITD), Wroclaw, Poland), supplemented with 10% Fetal Bovine Serum (FBS; Capricorn Scientific GmbH, Ebsdorfergrund, Germany), 2 mM glutamine, and antibiotics (100 U/mL penicillin, 100 μg/mL streptomycin; Merck Millipore, Darmstadt, Germany). The cells were seeded onto 96-well plates at a density of 3 × 10^3^ cells per well in 100 μL of the growth medium and incubated at 37 °C with 5% CO_2_ for 24 h or until they reached 80% confluence. NHDF cells were incubated for 24 h with 500 μg/mL of peptide and their Cu­(II) and Zn­(II) complexes (molar ratio, 1:1). Cell proliferation was assessed using the standard MTT (3-(4,5-dimethylthiazol-2-yl)-2,5-diphenyltetrazolium bromide) assay (Merck Millipore, Darmstadt, Germany). Cell viability was calculated using the following formula

$$cell\;viability\left(\%\right)=\left[\frac{\left(A_{treatment}-A_{blank}\right)}{\left(A_{control}-A_{blank}\right)}\right]\times 100$$

where A represents absorbance at 570 nm. Measurements were made in three independent experiments, each performed in triplicate.

## Results and Discussion

### EGR 2D-aa Ligand

#### Enzymatic Stability of the Peptidomimetic

Trypsin digestion experiments were performed to assess the proteolytic stability of the native peptide and its peptidomimetic analogue. The native peptide, composed entirely of l-amino acids, was readily cleaved by trypsin, as confirmed by the presence of multiple signals in the MALDI-TOF MS spectrum, including a characteristic signal at *m*/*z* 870.398 corresponding to the HHHQSPK fragment (Figure S2A). In contrast, this signal was absent in the spectrum of the peptidomimetic EGR 2D-aa (Figure S2B), demonstrating that substitution with d-amino acids effectively enhances resistance to enzymatic degradation at this site.

#### Deprotonation Constants

Based on potentiometric titrations, ten deprotonation constants (Table 1, Figure S3) were established for EGR 2D-aa peptidomimetic MUC7 fragment. The determined values correspond to those reported in the literature for similar systems.^23,33,56–60^ The first three constants (p*K*_a_ = 3.36; 3.58, and 4.21) can be assigned to the deprotonation of three carboxylic side chains of glutamic acid. The next five constants (p*K*_a_ = 4.89, 5.62, 5.94, 6.44, and 6.8) are related to the deprotonation of five histidine imidazole groups. The last two values (p*K*_a_ = 7.67; 10.07) correspond to the deprotonation of the N-terminal amine group and the lysine side-chain amine, respectively. The determined p*K*_a_ values for the amino acid residues (both l- and d-enantiomers) are consistent with those found in the literature.^61–65^

**Table 1:** Deprotonation Constants (pK_a_) for the EGR 2D-aa Peptide and Stability Constants (log β) for Its Complexes with Cu­(II) and Zn­(II) Ions in Aqueous Solution of 4 mM HClO_4_ with *I* = 100 mM NaClO_4_ at 298 K^e^

EGR 2D-aa				Cu(II)-EGR 2D-aa			Zn(II)-EGR 2D-aa		
species	log β_jk_ ^a^	p*K*_a_ ^b^	residue	species	log β_jk_ ^c^	p*K*_a_ ^d^	species	log β_jk_ ^c^	p*K*_a_ ^d^
[H_10_L]^10+^	58.58(1)	3.36	Glu	[CuH_5_L]^7+^	42.46(1)		[ZnH_4_L]^6+^	35.18(1)	
[H_9_L]^9+^	55.22(1)	3.58	Glu	[CuH_4_L]^6+^	37.8(2)	4.66	[ZnH_3_L]^5+^	29.47(0)	5.71
[H_8_L]^8+^	51.64(1)	4.21	Glu	[CuH_3_L]^5+^	32.92(1)	4.88	[ZnH_2_L]^4+^	23.34(0)	6.13
[H_7_L]^7+^	47.43(1)	4.89	His	[CuH_2_L]^4+^	26.97(1)	5.95	[ZnHL]^3+^	16.29(0)	7.05
[H_6_L]^6+^	42.54(1)	5.62	His	[CuHL]^3+^	20.15(2)	6.82	[ZnL]^2+^	8.31(0)	7.98
[H_5_L]^5+^	36.92(1)	5.94	His	[CuL]^2+^	13.17(1)	6.98	[ZnH_–1_L]^+^	–0.72(1)	9.03
[H_4_L]^4+^	30.98(1)	6.44	His	[CuH_–1_L]^+^	5.16(2)	8.01	[ZnH_–2_1L]	–10.95(2)	10.23
[H_3_L]^3+^	24.54(1)	6.80	His	[CuH-_2_L]	–4.61(2)	9.77	[ZnH_4_L]^6+^	35.18(1)	
[H_2_L]^2+^	17.74(1)	7.67	H_3_N^+^	[CuH_–3_L]^−^	–15.06 (2)	10.45			
[HL]^+^	10.07(0)	10.07	Lys						

a Constants are presented as cumulative log β_jk_ values. β­(H_*j*_L_*k*_) = [H_*j*_L_*k*_]/([H]_*j*_[L]_*k*_), in which [L] is the concentration of the fully deprotonated peptide.

b p*K*_a_ values of the peptides were derived from cumulative constants: p*K*_a_ = log β­(H_*j*_L_*k*_) – log β­(H_*j*–1_L_*k*_).

c Cu­(II) and Zn­(II) stability constants are presented as cumulative log β_*ijk*_ values. L stands for a fully deprotonated peptide ligand that binds Cu­(II) and Zn­(II) ions: β­(M_*i*_H_*j*_L_*k*_) = [M_*i*_H_*j*_L_*k*_]/([M]*i*[H]*j*[L]*k*), where­[L] is the concentration of the fully deprotonated peptide.

d p*K*_a_ = logβ (M_*i*_H_*j*_L_*k*_) – logβ­(M_*i*_H_*j*–1_L_*k*_).

e C_L_ = 0.4 mM; molar ratio M/L – 0.9:1. The standard deviations are reported in parentheses as uncertainties on the last significant figure.

In the measured pH range (2.0–12.0), the C-terminal carboxyl group, the carboxylic side-chain of aspartic acid, and the five arginine side-chains were not detected.^66,67^ The peptide behaves as an acid, [H_17_L]^12+^. However, as mentioned above, seven amino acid residues (five Arg and two acidic residues) lie outside the reliable operating range of the electrode. Therefore, in our calculations, we accounted for both the appropriate protonation states of residues that may undergo deprotonation within the investigated pH range and for the charges arising from all possible residues. This consideration is essential not only for the accurate determination of thermodynamic equilibria in the system but also for theoretical calculations.

### Coordination Modes of Metal-EGR 2D-aa Complexes

A variety of experimental methods were employed to investigate the precise stoichiometry, structural characteristics, and thermodynamic behavior of metal-EGR 2D-aa complexes. These techniques included ESI-MS, multiple potentiometric titrations, and spectroscopic analyses using UV–vis, CD, and EPR. In addition, computational studies were employed to support the experimental results.

#### Cu­(II)-EGR 2D-aa Complexes

The ESI-MS analysis confirmed the purity of the studied peptidomimetic ligand and the presence of only an equimolar metal to ligand stoichiometry in the complex formed in solution. No bis- or polynuclear complexes were detected using either ESI-MS (Figure S4, Table S1) or potentiometry (Table 1). The most intense signals (*m*/*z*) for Cu­(II)-EGR 2D-aa system were detected and aligned with the corresponding species. Both the experimental and simulated spectra showed agreement in terms of signals and isotopic distributions, validating the interpretation (Figure S4A, Table S1). The most intense signal corresponds to the [CuL]^4+^ complex with an *m*/*z* value of 665.313, [CuL]^5+^ complex with an *m*/*z* value of 532.452, while the signal at *m*/*z* = 443.878 corresponds to the [CuL]^6+^ species. Other signals observed in the spectra mainly represent perchlorate adducts of the ligands and complexes, along with trace impurities from the measuring device.

The stability constants for Cu­(II) complexes with the EGR 2D-aa peptide were determined from titration curves, and in potentiometric analysis identified nine complex species in this system (Figure S5A).

The peptide starts to bind Cu­(II) ion at pH 3.5. The first observed complex species is [CuH_5_L]^7+^, reaching its maximum concentration at pH 4.4 (Figure S5A), and is most likely associated with the involvement of two His residues in Cu­(II) ion coordination. The 2N coordination mode is confirmed by the d–d band in UV–vis spectrum with a maximum absorbance at 623 nm (Figure 4A), while the involvement of N_im_ group is suggested by the presence of a CD band with a negative Cotton effect at 238 nm (Figure 4B).^68^ The next complex species, [CuH_4_L]^6+^, predominates at pH 4.8 and is likely associated with the deprotonation and binding of the next histidine residue, as indicated by the decrease in the p*K*_a_ value from 5.94 (in the free ligand) to 4.66 (in the complex). The band with a maximum absorbance at 604 nm in the UV–vis spectrum (Figure 4A) and EPR parameters A_∥_ = 184, g_∥_ = 2.22 (Figure S6) confirms the presence of a complex with 3N coordination. Notably, at this pH, the 2N-coordinated species is still present, which is supported by the two sets of EPR parameters (Figure S6) and by distribution diagram (Figure S5A).

**Figure 4:**
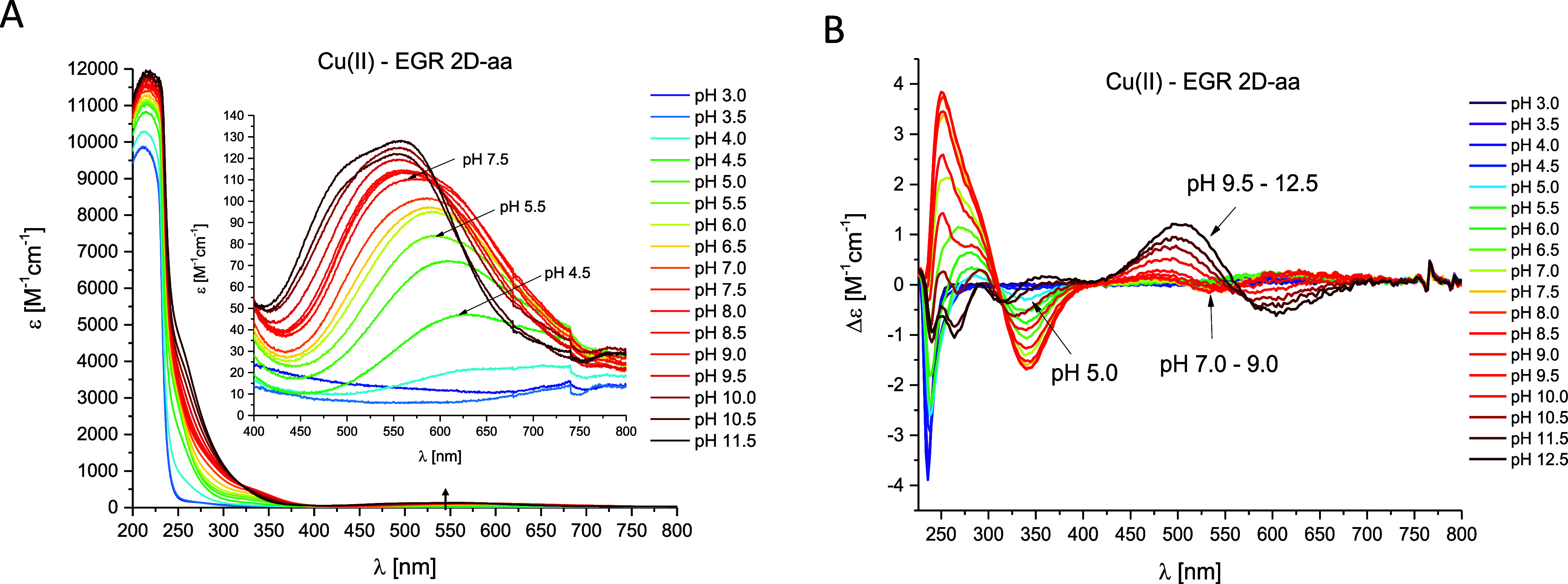
pH-dependent spectra: (A) UV–vis spectra (inset on the right corresponds to the d–d transition region), and (B) CD spectra for the Cu­(II)-EGR 2D-aa system in aqueous solution of 4 mM HClO_4_ with *I* = 100 mM NaClO_4_. Optical path length of 1 cm. C_L_ = 0.4 mM; molar ratio M/L–0.9:1.

The subsequent complex species, [CuH_3_L]^5+^ and [CuH_2_L]^4+^, form as a result of the stepwise deprotonation of two His residues (with p*K*_a_ values of 4.88 and 5.95, respectively). The lowering of the p*K*_a_ values compared to those of the free ligand (p*K*_a_ = 6.44 and 6.80, respectively), without notable changes in the spectroscopic data, is indicative of a specific coordination mode, known as ‘polymorphic binding states‘. This type of interaction refers to a situation in which two (or more) distinct sets of His imidazole residues are coordinated to the metal ion, with one His residue being shared by both sets.^69^ For the [CuH_3_L]^5+^ and [CuH_2_L]^4+^ species, the experimental data suggest the coexistence of distinct Cu­(II) complexes with 3N coordination modes in equilibrium. Interestingly, previous studies of the native MUC7 fragment (EGR L-aa) have shown that Cu­(II) is also bound through polymorphic binding states but involving at most two His residues.^33^ These differences in the number of coordinated nitrogen atoms are supported, for example, by the wavelength of the absorption maximum at pH 5.5 for both complexes (Figure S7).

Additionally, as shown in Figure 5, computational studies also support the presence of Cu­(II) complexes with 3N_im_ donor sets coordinated in different ways within the [CuH_3_L]^5+^ species. At pH 5.4, the three most stable structures were identified, in which Cu­(II) is coordinated by (A) His-7, His-11, and His-15, with an energy of −4168464.98 kcal/mol; (B) His-11, d-His-14, and His-15, with an energy of −4168462.53 kcal/mol; and (C) His-7, His-15, and His-16 with energy −4168459.08 kcal/mol. The most stable structure found is the complex denoted as A. The energy differences between the obtained models range from 2.45 to 5.90 kcal/mol. Despite the prediction limitations of the computational studies due to the complexity of the conformational landscape in solution, the revealed conformations support the transition of different metal coordination as a function of pH.

**Figure 5:**
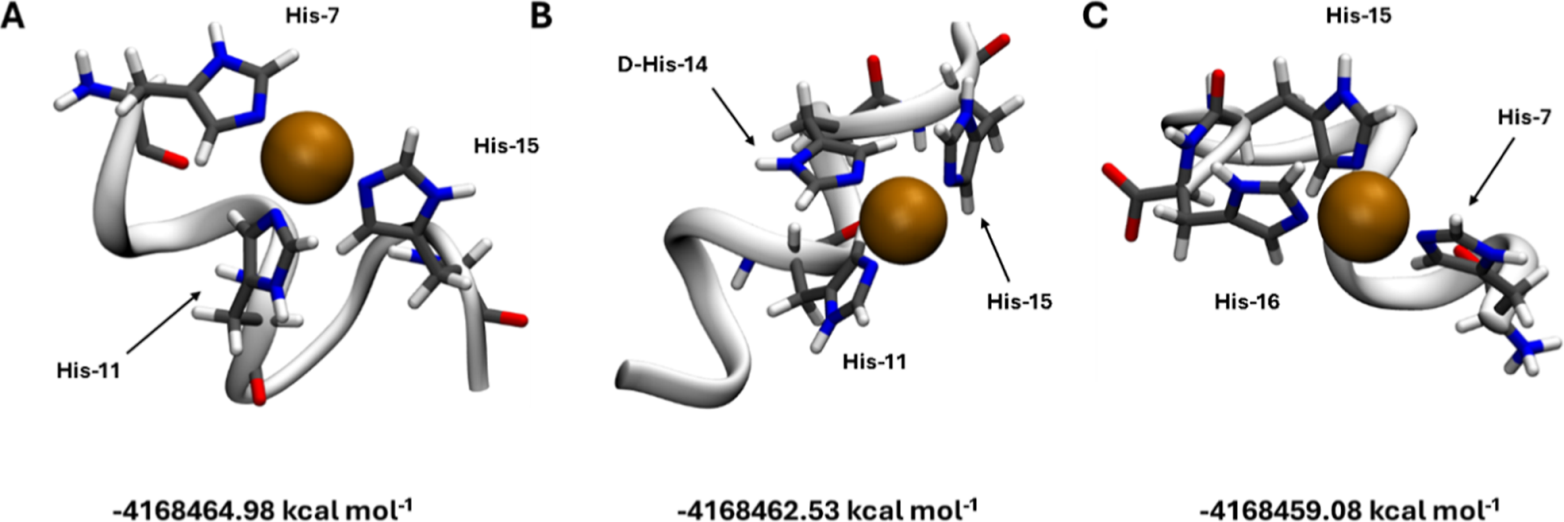
Short models of the three most stable structures of [CuH_3_L]^5+^ with the following coordinating residues: (A) His-7, His-11, His-15; (B) His-11, d-His-14, His-15, (C) His-7, His-15, His-16, obtained from models reproducing complex formation at pH 5.4.

The next complex species, [CuHL]^3+^, which reaches its maximum concentration at pH 6.9, is most likely formed through coordination of the first amide group. In the CD spectrum, new weak bands appear at 534 and 598 nm, together with a shift of the CT band from 280 to 254 nm that suggests a N_am_ → Cu­(II) charge-transfer transition.^70,71^ Additionally, in the UV–vis spectrum, a slight band broadening toward shorter wavelengths (Figure 4A), together with subtle changes in the EPR parameters at pH 7.0: A_∥_ = 185 and g_∥_ = 2.22 (Figure S6), suggest the formation of a 3N or 4N complex. Notably, for the native peptide at pH 7.0, polymorphic states involving {2N_im_} coordination were still observed, whereas coordination of the amide nitrogen atom occurred above pH 7.5.^33^

The next species in the Cu­(II)-EGR 2D-aa system, [CuL]^2+^, which predominates in the pH range pH 7.0–7.9, can be attributed to nonbonding deprotonation of the N-terminal amine group, as indicated by the absence of a significant decrease in the p*K*_a_ value relative to the free ligand (p*K*_a_ = 6.98 and 7.67, respectively). The spectral parameters remain unchanged (except for an increase in the intensity of the band with positive Cotton effect at 254 nm in the CD spectrum), suggesting the same coordination mode as in the previous complex species, with the {3N_im_, 1N_am_} donor set.

Theoretical calculations revealed three stable structures (Figure 6) corresponding well with the experimental findings. It is worth underlining that no structures involving the N-terminal amine group were considered in the theoretical study for this species, as no evidence of its involvement was found in the potentiometric and spectroscopic experiments. However, this does not exclude the possibility of its stabilizing effect on the Cu­(II) ion due to the flexibility of the experimental models as well as complexity of the possible conformations from the thermodynamics point of view.

**Figure 6:**
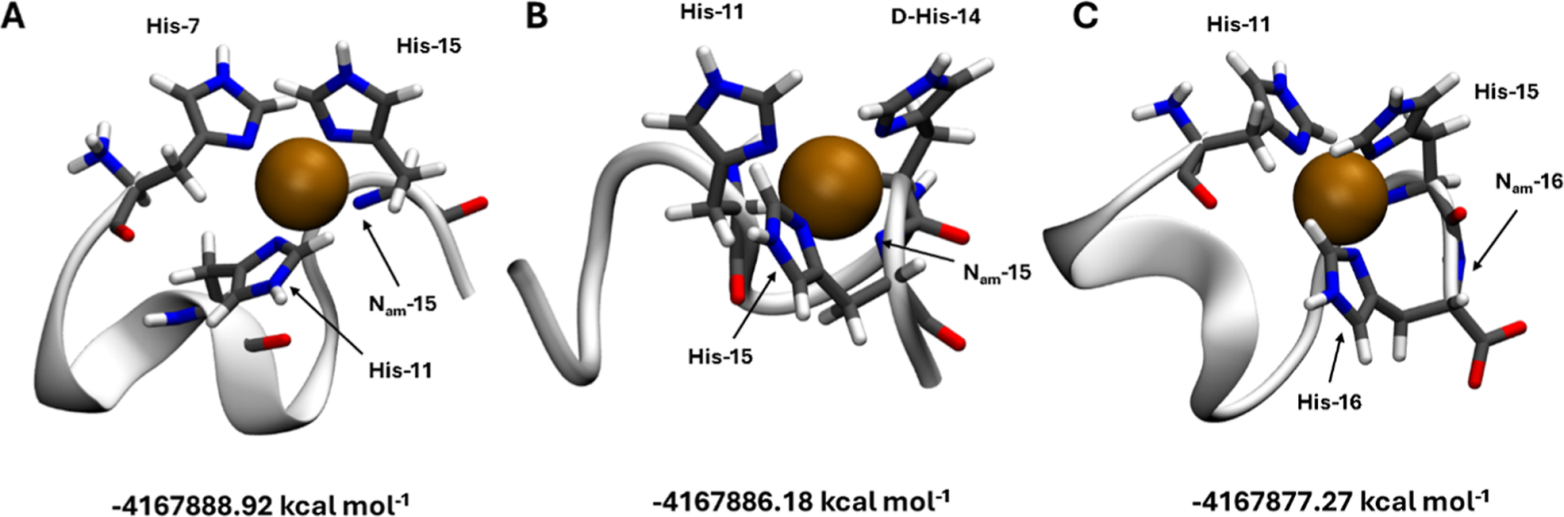
Short models of the three possible structures of [CuL]^2+^ with the following coordinating residues: (A) His-7, His-11, His-15, N_am_-15; (B) His-11, d-His-14, His-15, N_am_-15; and (C) His-11, His-15 His-16, N_am_-16, obtained for models reproducing complexation at pH 7.4. The models were constructed on the basis of potentiometric and spectroscopic findings.

While the CD spectra at pH 7.0–9.0 reveal comparable His–imidazole/amide coordination in both Cu­(II) complexes (Figure S8A), enhanced stability is observed for the peptidomimetic (Figure 7).^33^ This effect can most likely be attributed to two factors: (i) the involvement of a larger number of histidine residues (three in the peptidomimetic vs two in the native peptide) and (ii) the spatial arrangement of the coordinating residues, where the presence of a d-His residue may further stabilize the complex through its stereochemical orientation.

**Figure 7:**
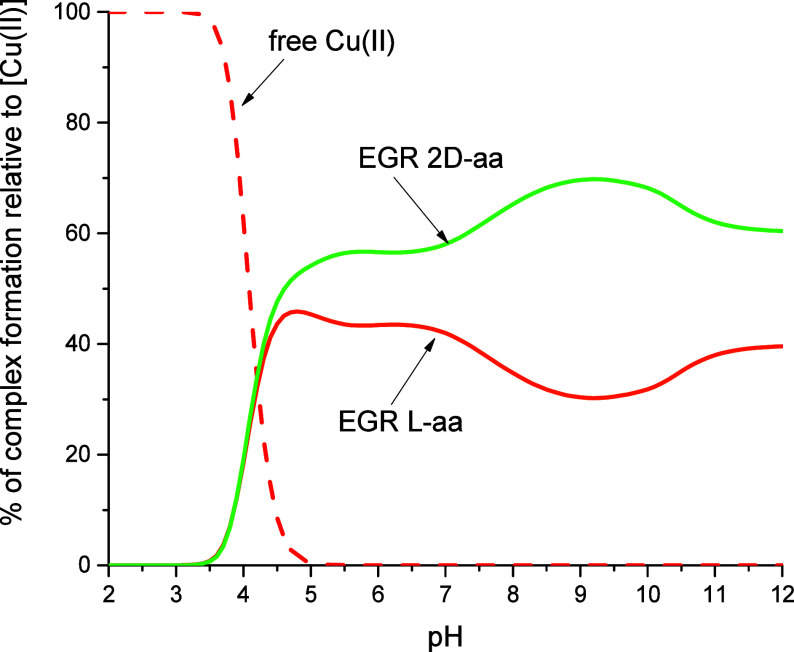
Competition plot, describing complex formation at different pH values in a hypothetical situation, in which equimolar amounts of all reagents are mixed, for Cu­(II) complexes with EGR L-aa (MUC 7 native fragment) and EGR 2D-aa (peptidomimetic fragment) based on potentiometric data (Table 1 and ref 33) Conditions: T = 25 °C, [Cu­(II)] = [EGR 2D-aa] = [EGR L-aa] = 1 mM.

The formation of the next complex species, [CuH_–1_L]^+^, is associated with the involvement of an additional amide nitrogen in the coordination sphere, replacing one of the imidazole nitrogens and forming a {2N_im_, 2N_am_} donor set. The subsequent complex species arises from the coordination of the third amide nitrogen atom, which displaces another N_im_ donor and results in a complex with {1N_im_, 3N_am_} donor set and typical square-planar geometry. It is supported by the CD spectrum showing characteristic bands with a positive Cotton effect at 490 nm and a negative Cotton effect at 586 nm (Figure 5B). Further proton loss leads to the formation of the [CuH_–3_L]^−^ complex species, with the corresponding p*K*_a_ value (10.07) arising from deprotonation of a nonbinding Lys residue. Above pH 10, both the native and peptidomimetic ligands form Cu­(II) complexes with the same coordination mode ({1N_im_, 3N_am_}). However, an intriguing observation is the difference in the CD spectra (Figure S8B) between the Cu­(II) complexes of the native peptide and the peptidomimetic above pH 9.5. The nearly mirror-image spectra in the Vis region, together with the differences observed in the UV range (for the charge-transfer bands), suggest that the d-His residue and the amide nitrogen of the peptide bond between d-His and d-Arg are most likely involved in Cu­(II) coordination. The above-described experimental results for the Cu­(II) complex species are summarized in Table S2.

#### Zn­(II)-EGR 2D-aa Complexes

Analysis of ESI-MS spectra (Figure S4B, Table S1) and potentiometric titrations (Table 1) confirmed the formation of only equimolar complexes with Zn­(II), similarly to what was observed for the Cu­(II) complexes. The most intense m/z signals for each system matched the expected species. Experimental and simulated spectra exhibited consistent signals and isotopic distributions, supporting the accuracy of the assignments. The most intense peak in the spectrum corresponds to the [ZnL]^4+^ complex at m/z value of 665.563 (Figure S4B), followed by the [ZnL]^3+^ complex at m/z 887.082 (Table S1). Additional signals mainly arise from perchlorate adducts of the ligands and complexes, as well as minor impurities originating from the measuring device.

The titration curves for the Zn­(II)-EGR 2D-aa system were best fitted by assuming the formation of seven complex species (Table 1 and Figure S5B). The first complex species, [ZnH_4_L]^6+^, appears at pH 4.4 and reaches its maximum concentration at pH 5.5, where Zn­(II) is coordinated by three imidazole residues, forming a {3N_im_} donor set.

To further elucidate the Zn­(II) binding mode at this pH value, theoretical simulations based on DFT were performed (Figure 8). For the [ZnH_4_L]^6+^ species, the semiempirical approach indicated that the three lowest-energy structures involve coordination by the following sets of histidine residues: (A) His-7, d-His-14, His-16; (B) His-7, His-11, His-15; and (C) His-7, d-His-14, His-15. The energy differences among these three proposed coordination models range from 1.74 to 12.75 kcal/mol. Concerning the structure B, during the energy minimization, we have noticed that one water molecule increased the distance to the metal center, forming an intermolecular hydrogen bond with the second water molecule considered in the model.

**Figure 8:**
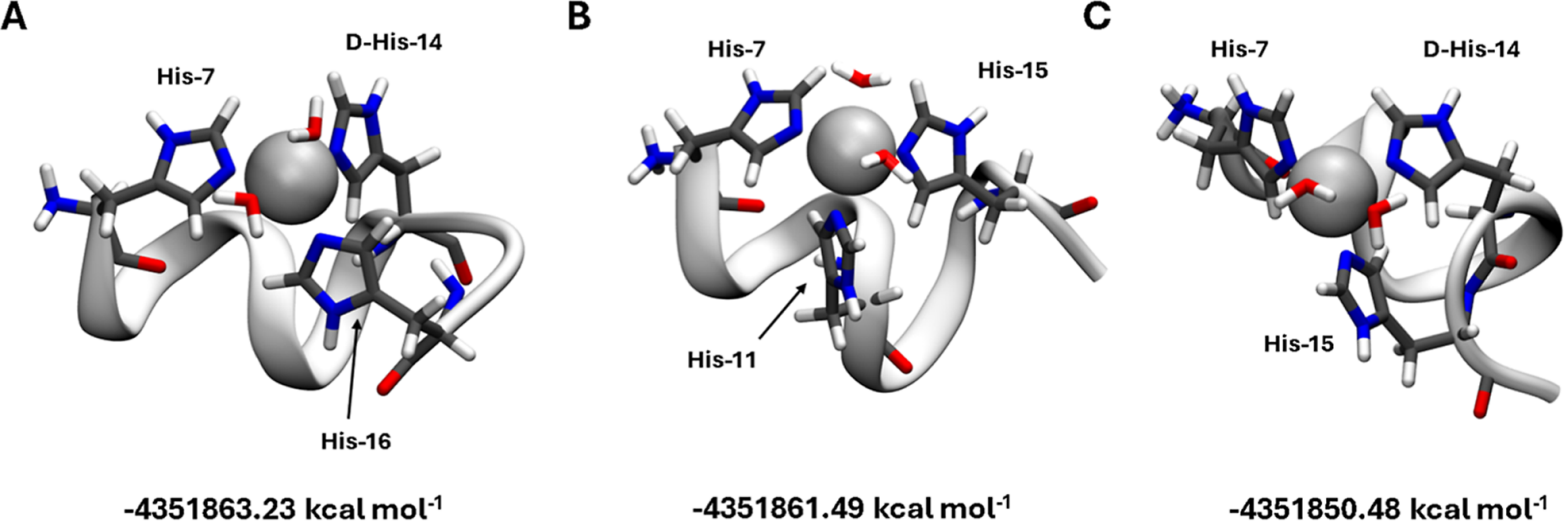
Short models of three most stable structures of [ZnH_4_L]^+^ with following coordinating residues: (A) His-7, d-His-14, His-16, (B) His-7, His-11, His-15, and (C) His-7, d-His-14 His-15 obtained for models reproducing complexation at pH 5.4.

The subsequent complexes, [ZnH_3_L]^5+^ and [ZnH_2_L]^4+^, exhibit decreased p*K*_a_ values for the histidine residues compared to the free ligand (p*K*_a_ = 6.44 → p*K*_a_ = 5.71 and p*K*_a_ = 6.8 → p*K*_a_ = 6.13, respectively), indicating ‘polymorphic binding states’ involving the {3N_im_} donor set. This coordination mode was also observed for the Zn­(II) complex of the native peptide studied previously.^33^ Similar coordination and thermodynamic stability for both complexes is further supported by the competition plot (Figure 9), where the native l-amino acids peptide (EGR L-aa) shows a comparable affinity for Zn­(II) ions to that of its peptidomimetic analogue containing two d-amino acids (EGR 2D-aa).

**Figure 9:**
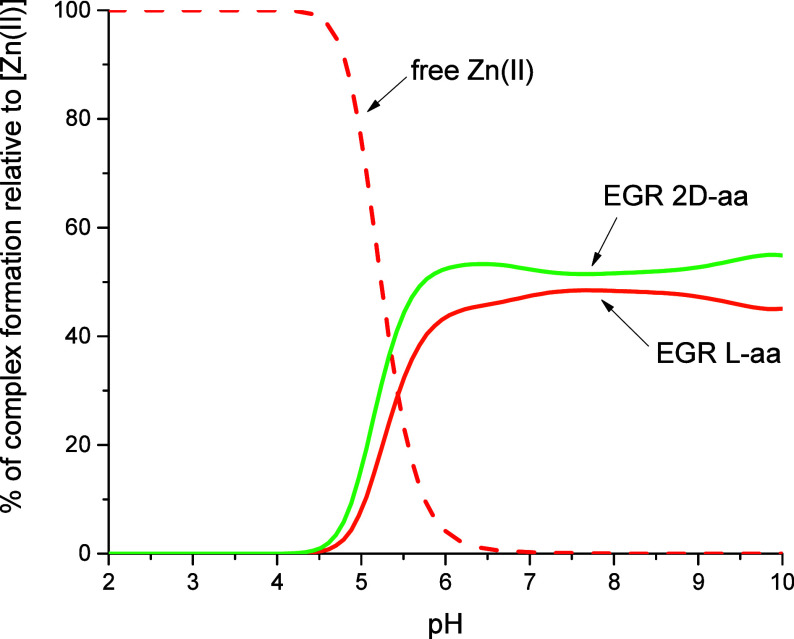
Competition plots describing complex formation at different pH values in a hypothetical situation, in which equimolar amounts of all reagents are mixed, for Zn­(II) complexes with EGR L-aa (MUC 7 native fragment) and EGR 2D-aa (peptidomimetic fragment) based on potentiometric data (Table 1 and ref 33), Conditions: T = 25 °C, [Zn­(II)] = [EGR L-aa] = [EGR 2D-aa] = 0.001 M.

The formation of the next complex species, [ZnHL]^3+^, reaching its maximum concentration at pH 7.5, can be attributed to coordination of the N-terminal amine group, resulting in a {3N_im_, NH_2_} donor set.

Theoretical calculations support this coordination mode, and the three stable structures with a {3N_im_, NH_2_} donor set were identified (Figure 10). The coordinating imidazole nitrogen atoms originate from the following residues: (A) His-7, His-11, d-His-14; (B) His-11, His-15, His-16 and (C) His-7, d-His-14, His-16. Energy minimization of the truncated models using r^2^SCAN-3c composite method confirmed the stability of all short models, suggesting that these complexes represent the most favorable arrangements in terms of Zn­(II) binding affinity. Among the models obtained at pH 7.4, the smallest energy difference was observed between (A) and (B) (1.67 kcal/mol), whereas the largest differences occurred between (A) and (C) (7.55 kcal/mol). It is worth to underline that in the model B, one water molecule exhibited a very large mobility, which resulted in its dissociation.

**Figure 10:**
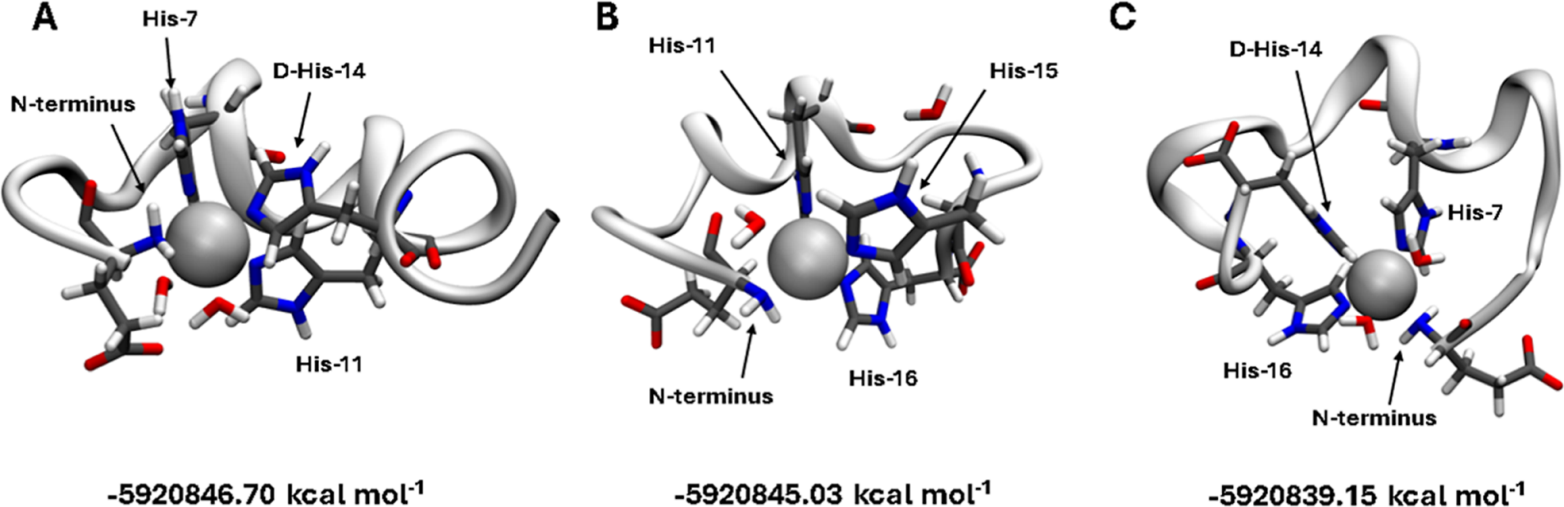
Short models of the three most stable structures of [ZnHL]^3+^ species with the following coordinating residues: (a) N-terminus, His-7, His-11, d-His-14, (b) N-terminus, His-11, His-15, His-16, (c) N-terminus, His-7, d-His-14, His-16 obtained for pH environment of 7.4 (lower panel).

The subsequent complex species, [ZnL]^2+^ and [ZnH_–1_L]^+^, with assigned p*K*_a_ values of 7.98 and 9.03, respectively, are formed as a result of the stepwise deprotonation of two aqua ligands in the coordination sphere of the Zn­(II) ion. The final species, [ZnH_–2_L], with p*K*_a_ = 10.23, is attributed to deprotonation of the noncoordinating Lys residue.

Interestingly, the ligand under study also contains the HEXXH motif (−HELRH−), characteristic of many metalloproteinases, in which the Zn­(II) ion is coordinated by two His residues and the carboxyl group of Glu residue.^72–74^ To assess the likelihood that Zn­(II) binds through this motif, we compared the thermodynamic stability, i.e., the Zn­(II)-binding efficiency, of other human salivary peptides containing the same sequence motif, such as histatin 5, as well as a closely related motif (HEXXXH) present in the C-terminal fragment of a chemokine CCL-28, both of which are known to bind Zn­(II) through these specific sites.^23,63^ As shown in the competitive plot (Figure 11A), the comparable Zn­(II)-binding efficiencies of these ligands suggest that, for the EGR 2D-aa peptide, coordination through the HEXXH motif cannot be excluded, a conclusion also supported by DFT calculations (Figure 11B). However, this is not the dominant coordination mode and most likely exists in equilibrium with the 3N_im_-bound species.

**Figure 11:**
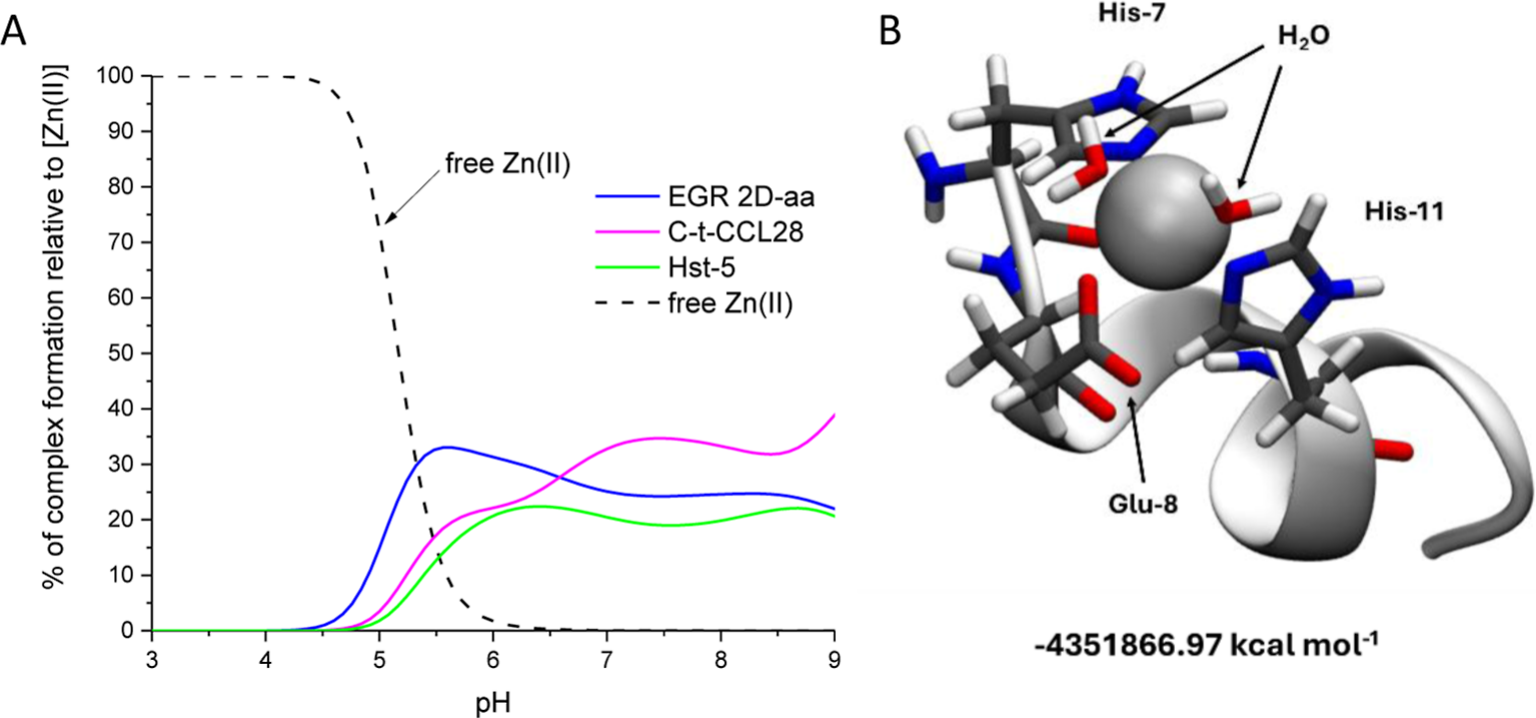
(A) Competition plot describing complex formation at different pH values in a hypothetical situation, in which equimolar amounts of all reagents are mixed, for Zn­(II) complexes with EGR 2D-aa (peptidomimetic fragment), C-t-CCL28 (C-terminal fragment of Chemokine 28) and Hst-5 (Histatin 5) based on potentiometric data (Table 1 and refs 23, 33 and 75 ). Conditions: T = 25 °C, [Zn­(II)] = [EGR 2D-aa] = [C-t-CCL28] = [Hst-5] = 0.001 M. (B) The fragment of EGR 2D-aa (HEXXXH motif) with zinc ion and two water molecules. The zinc ion binds to His-7, His-11 and Glu-8 residues. The model was stable at the r^2^SCAN-3c level of theory. The simulations were performed at pH 5.4.

### Far-UV CD Studies of MUC7 Peptidomimetic Fragments and Their Complexes

One of the possible mechanisms of action of antimicrobial peptides involves their interaction with the cell membrane, and the secondary structure is a key factor governing this process.^76^ Importantly, interactions with metal ions can influence the secondary structure formation of AMPs, thereby affecting their antimicrobial activity.^9^ Therefore, far-UV CD (Figure S9) was used to determine the structure of the peptidomimetic fragment of the studied peptidomimetic, its complexes with Cu­(II) and Zn­(II), and to assess the influence of metal ion coordination on the secondary structure. For the Cu­(II)-EGR 2D-aa system at pH 5.4 (Figure S10A), a small tendency toward α-helical structure formation is observed, as supported by the presence of characteristic bands.^77^ At this pH, the dominant contribution arises from the [CuH_3_L]^5+^ complex species with a {3N_im_} donor set, present in polymorphic binding states. Such behavior is often encountered in histidine-rich peptides, as demonstrated in both experimental and theoretical studies.^69,78^ Interestingly, when compared with the Cu­(II) complexes of the native peptide EGR L-aa (Figure 12A), a similar tendency is observed in both cases. It should be noted, however, that the α-helical contribution in the Cu­(II)-EGR 2D-aa system is slightly lower than in the Cu­(II)-EGR L-aa system, as evidenced by the less intense negative Cotton effect band at ∼224 nm and by the positive Cotton effect band shifted toward shorter wavelengths. This difference may arise from a residual contribution of a 2N_im_-coordinated species (coexisting with 3N_im_ complex) in solution (see section above).

**Figure 12:**
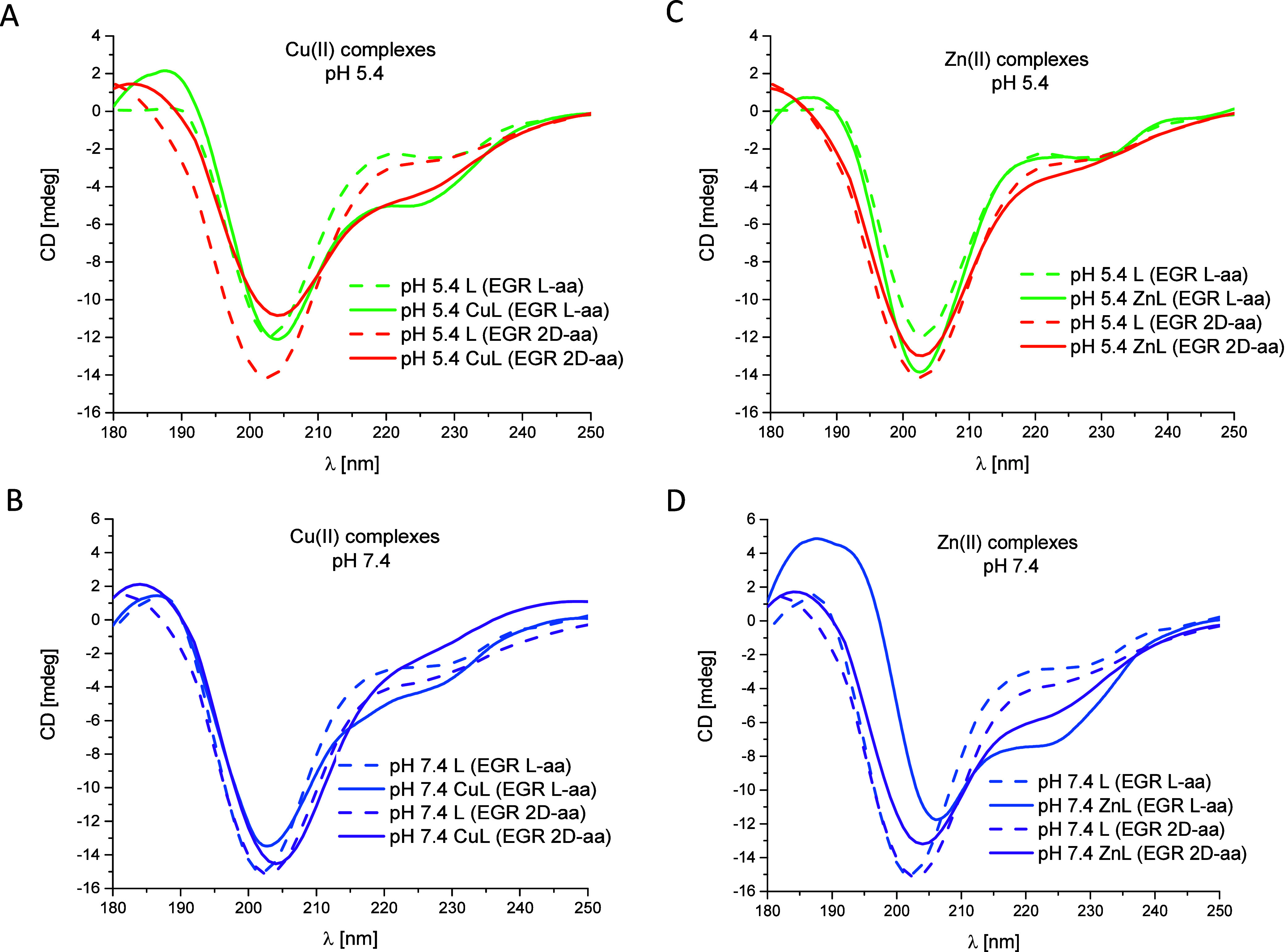
Comparison of Far-UV CD spectra at 180–250 nm for EGR L-aa and EGR 2D-aa with (A) Cu­(II) at pH 5.4, (B) Cu­(II) at pH 7.4; (C) Zn­(II) at pH 5.4 and (D) Zn­(II) at pH 7.4 in aqueous solution of 4 mM HClO_4_ with *I* = 100 mM NaClO_4_; molar ratio M/L 0.9:1; the optical path length = 0. Two mm; C_L_ = 0.3 Mm; the dashed lines correspond to peptide spectra. Data for EGR L-aa are based on ref 33.

At pH 7.4, where the [CuL]^2+^ species predominates, an unstructured conformation is observed (Figure S10B), which is consistent with a change in coordination mode from 3N to 4N (from a {3N_im_} to a {3N_im_, 1N_am_} donor set). In contrast, in the Cu­(II)-EGR L-aa system, a tendency toward helical structure is still observed, associated with still observed polymorphic binding states involving {2N_im_} donor set (Figure 12B).

For the Zn­(II) complexes, the spectra indicate that at pH 5.4 no specific secondary structure is present after metal ion coordination, whereas at pH 7.4 a slight tendency to α-helical structure formation is observed (Figure S10). The change in secondary structure coincides with the change in the coordination mode of the dominant complex species, from 3N ({3N_im_}, polymorphic binding states) at pH 5.4 to 4N {3N_im_, –NH_2_} at pH 7.4. Similar effects are observed when comparing the influence of Zn­(II) ion on the structure of the native EGR L-aa ligand and its peptidomimetic analogue (Figure 12C,D), with a significantly greater helical contribution in the Zn­(II)-EGR L-aa system (Figure 12D).

### ROS Detection

The generation of ROS was validated through ascorbate consumption experiments using UV–vis and fluorescence spectroscopy. To assess the production of hydroxyl radicals (OH^•^) by Cu­(II)-EGR 2D-aa in the presence of ascorbate, three comparative systems were analyzed: (i) Cu­(II) with ascorbate in buffer, (ii) Cu­(II)-EGR 2D-aa with ascorbate in buffer, and (iii) EGR 2D-aa with ascorbate in buffer (Figure S11). The most pronounced ascorbate oxidation occurred with free Cu­(II), consistent with a previous report.^79^ Nonetheless, Cu­(II)-EGR 2D-aa system also exhibited considerable OH^•^ production in the presence of ascorbate, whereas the free peptide showed minimal OH^•^ generation (Figure S11).

Moreover, ROS production was confirmed using a fluorescence assay with coumarin-3-carboxylic acid, a reliable probe for hydroxyl radicals generated via Cu­(II)-mediated oxidation of ascorbic acid (Asc).^80^ The highest level of ROS formation was observed for the Cu­(II)-EGR 2D-aa system in the presence of ascorbate after 25 min (Figure S12). The spectra exhibited the strongest emission at 452 nm, characteristic for 7-OH–CCA formed upon HO^•^ trapping. This effect supports that the Cu­(II)-EGR 2D-aa complex actively promotes ROS generation under the tested conditions.

It should be noted that the term ROS encompasses chemically distinct species formed via different pathways. The assays applied here intend to prove overall Cu-dependent oxidative reactivity under reducing conditions rather than to fully differentiate among all ROS species. The CCA fluorescence assay, widely employed as a trapping method for hydroxyl radicals, together with ascorbate consumption experiments, supports that OH^•^ is a major product formed in the Cu­(II)-EGR 2D-aa system. Mechanistically, in the presence of ascorbate, Cu complexes may undergo Cu­(II)/Cu­(I) redox cycling, enabling Fenton-like processes that lead to hydroxyl radical formation. The reduced rate of ascorbate oxidation observed for the Cu­(II)-EGR 2D-aa complex compared to free Cu­(II) indicates that peptide coordination modulates, but does not suppress, the redox activity of the metal center. In contrast, the peptide alone showed negligible ROS production, confirming that metal coordination is required for this oxidative pathway.

In our experimental studies, we observed a clear tendency toward (i) the formation of thermodynamically stable complexes, (ii) the induction of ordered secondary structures upon coordination with Cu­(II)/Zn­(II) ions, and (iii) the efficient generation of hydroxyl radicals in Cu­(II) complexes, which combining together may translate into effective antimicrobial activity, as previously reported in the literature.^75,81,82^

### *In Vitro* Antimicrobial and Antibiofilm Activities

Infections caused by pathogens can lead to both acute and chronic oral diseases, which can significantly impact overall health and quality of life.^83^ Oral infections are often associated with a disruption of the balance between the host’s immune system and microbial communities, leading to conditions such as periodontitis, gingivitis, and dental caries. Common multidrug-resistant pathogens associated oral infections include bacteria such as *S. mutans* and *S. sanguinis*, and the yeast *C. albicans*.^84^ Additionally, the ability of these pathogens to form biofilms further complicates treatment, as biofilm-associated cells exhibit increased resistance to antimicrobial agents compared to planktonic cells.^85^ Therefore, there is an urgent need to develop new antimicrobial treatments that can overcome these challenges and target both planktonic and biofilm forms of these pathogens.

Given that the pH of saliva typically ranges from 5.3 to 7.8,^86^ and varies depending on an individual’s health status, understanding the antimicrobial activity of agents at different pH levels is critical.^86,87^ The pH can influence the efficacy of antimicrobial agents, as acidic conditions may enhance or hinder their activity. Therefore, we evaluated the antimicrobial activity of the peptide and its metal complexes against six bacterial strains and one fungal strain at pH values of 5.4 and 7.4 to better understand their potential as broad-spectrum antimicrobial agents in oral environments.

The antimicrobial effectiveness of EGR 2D-aa is notably affected by both the pH and the presence of Cu­(II) and Zn­(II) ions. Remarkably, the peptidomimetic exhibits antimicrobial properties only when complexed with Cu­(II) and Zn­(II) ions, regardless of the pH value. In addition, the EGR 2D-aa metal complexes demonstrate antimicrobial activity exclusively against two common oral cavity pathogens tested in this work: *S. mutans* and *S. sanguinis* (Table 2). Tables S3 and S4 show the complete antibacterial and anti-*Candida* assessment of the peptidomimetic complexes.

**Table 2:** Comparison of the *In Vitro* Antibacterial Activity of Peptide/Complexes (Top Row) and Peptidomimetic/Complexes (Bottom Row) Determined as a Minimal Inhibitory Concentration (MIC) (μg/mL); Antimicrobial Assays Were Performed in 10 mM MES Buffer (pH 5.4) and 10 mM HEPES Buffer (pH 7.4); n/d - Not Determined within the Concentration Range Used in This Study^a^ ^,^ ^33^

	*S. sanguinis*PCM 2335 pH 5.4	*S. sanguinis* PCM 2335 pH 7.4	*S. mutans* PCM 2335 pH 5.4	*S. mutans* PCM 2335 pH 7.4
EGR L-aa	n/d	n/d	n/d	n/d
Cu(II)-EGR L-aa	n/d	n/d	n/d	n/d
Zn(II)-EGR L-aa	n/d	500	n/d	n/d
EGR 2D-aa	n/d	n/d	n/d	n/d
Cu(II)-EGR 2D-aa	250	250	500	500
Zn(II)-EGR 2D-aa	125	250	250	500

a Data for the native peptide and its complexes were taken from the literature.

At pH 7.4 for *S. sanguinis*, the MIC values determined for the peptidomimetic’s complexes were 250 μg/mL for both Cu­(II) and Zn­(II) complexes. At pH 5.4, the MICs for *S. sanguinis* were 125 μg/mL for Zn­(II)-peptidomimetic, and 250 μg/mL for Cu­(II)-peptidomimetic (Table 2). Interestingly, metal ions such as copper and zinc did not exhibit antibacterial effects against Gram-positive bacteria, Gram-negative bacteria, or *C. albicans* strains within the concentration range of 0.3 to 38 μg/mL (Tables S5 and S6).

A comparison of the antimicrobial activity between the complexes of the native fragment^33^ and those incorporating the d-amino acids (Table 2) show that this modification enhances activity against *S. sanguinis* and, notably, induces antimicrobial activity against *S. mutans*, an effect not observed for the native fragment complexes.

Both metal-EGR 2D-aa complexes were further evaluated for their ability to inhibit biofilm formation (Table 3). *S. mutans* and *S. sanguinis* were allowed to develop biofilms in the presence or absence of each complex at sub-MIC concentrations. Both metal-peptidomimetic complexes exhibited significant inhibition of biofilm formation, demonstrating antibiofilm activity at 0.75 × MIC and 0.5 × MIC under both pH conditions. These results suggest that the biofilm inhibition mechanisms of the metal-peptidomimetic complexes are not solely related to bacterial growth inhibition but may also involve direct interaction with biofilm matrix components, offering potential for future therapeutic applications.

**Table 3:** Biofilm Quantification^a^

	Biofilm (%)				
Strain	Control	Cu(II)-EGR 2D-aa 0.75 × MIC	Cu(II)-EGR 2D-aa 0.5 × MIC	Zn(II)-EGR 2D-aa 0.75 × MIC	Zn(II)-EGR 2D-aa 0.5 × MIC
pH 7.4					
*S. mutans* PCM 2502	100 ± 1.2	35 ± 1.6	67 ± 1.5	25 ± 1.4	65 ± 1.7
*S. sanguinis* PCM 2335	100 ± 1.7	33 ± 1.2	60 ± 1.8	20 ± 0.5	62 ± 0.7
pH 5.4					
*S. mutans* PCM 2502	100 ± 1.1	24 ± 2.1	63 ± 1.9	23 ± 1.5	64 ± 1.6
*S. sanguinis* PCM 2335	100 ± 0.7	21 ± 1.6	55 ± 0.5	19 ± 1.4	60 ± 1.1

a The total amount of biomass of *S. mutans* and *S. sanguinis* incubated with metal ion complexes at sub-MIC concentrations was quantified with crystal violet. The results represent the averages of triplicate experiments ± SD.

The findings presented in this study are of significant biological relevance, as *S. mutans* is a well-established member of the oral microbiota under physiological conditions, playing a central role in dental plaque formation and the onset of dental caries. *S. mutans* contributes to the pathogenesis of dental caries by metabolizing dietary sugars to produce acids, primarily lactic acid, which demineralize tooth enamel and initiate caries formation.^88^ The ability of *S. mutans* to form biofilms on tooth surfaces further exacerbates its cariogenic potential, making it a primary target for antimicrobial interventions aimed at preventing tooth decay. In addition, *S. sanguinis*, while generally considered a commensal organism, can also play a role in the initiation of infective endocarditis if it translocates into the bloodstream, particularly following invasive dental procedures.^89^ This underscores the importance of targeting these pathogens in both oral health and systemic infection contexts.

### Cytotoxicity Assay

The EGR 2D-aa, Cu­(II)-EGR 2D-aa, and Zn­(II)-EGR 2D-aa were nontoxic to NHDF cells, resulting in cell viabilities of approximately 97%, 95%, and 97%, respectively (Table 4). These results indicate that the peptidomimetic and its Cu­(II) and Zn­(II) complexes hold potential as promising candidates for the development of antimicrobial metal-peptide complexes and merit further investigation in biological assays.

**Table 4:** Cell Viability Assay^a^

compound	cell viability (%)
EGR 2D-aa	97 ± 0.3
Cu(II)-EGR 2D-aa	95 ± 0.4
Zn(II)-EGR 2D-aa	97 ± 0.2

a NHDF cells were plated in a 96-well titer plate with 500 μg/mL of peptide and Cu­(II) and Zn­(II) complexes and incubated for 24 h. The percentage of cell viability was determined by MTT assay. The results represent the averages of triplicate experiments ± SD.

### Concluding Remarks

Salivary antimicrobial peptides, although promising therapeutics, are limited by poor biological stability. To address this, we characterized the EGR 2D-aa peptidomimetic, in which two l-amino acids were substituted with d-amino acids at the most protease-sensitive site. This modification conferred resistance to enzymatic digestion at the amino acid exchange site, while maintaining metal-binding capacity.

Complexation with Cu­(II) increased thermodynamic stability relative to the native peptide due to a distinct coordination mode, whereas Zn­(II) binding did not produce comparable changes. Quantum-chemical calculations supported the proposed Cu­(II)/Zn­(II) coordination modes, yielding 12 experimentally consistent structures and highlighting three lowest-energy forms as the most probable in solution. Despite conformational flexibility, truncated models reproduced these preferred structures, fully aligning with the experimental data.

Spectroscopic studies showed that Cu­(II) enhanced α-helical propensity at pH 5.4, with Zn­(II) inducing a similar effect at pH 7.4. In both cases, the helical content was lower in the peptidomimetic than in the native peptide complexes, consistent with known helix-disrupting effects of d-amino acid substitution.^90^ Although helix disruption is often associated with diminished antimicrobial activity,^91,92^ the studied peptidomimetic exhibited a pronounced increase in activity—an enhancement not observed for the native peptide-metal complexes. This improvement likely arises from combined metal-induced activation and increased conformational flexibility/loosening, which may facilitate membrane adsorption and a carpet-type disruption mechanism.^93,94^ The observed selectivity toward *S. mutans* and *S. sanguinis* is consistent with differences in membrane composition among Gram-positive species.^95,96^ For Cu­(II) complexes, high thermodynamic stability and the ability to generate ROS may further enhance antibacterial effects.

Although changes in the coordination mode ({2N_im_} ↔ {3N_im_} and the onset of amide coordination at higher pH) clearly influence complex stability across the saliva-relevant pH range, antimicrobial activity should be viewed as multifactorial. Metal complexation is a necessary condition for activity (the free EGR 2D-aa ligand is inactive), but the magnitude and selectivity of the antimicrobial effect likely reflect a combination of complex stability, Cu­(II)-activated oxidative reactivity, and physicochemical properties such as conformational flexibility and proteolytic resistance. Thus, coordination mode should be regarded as a modulating component of antimicrobial activity rather than a single determining factor.

Importantly, both metal-peptidomimetic systems efficiently inhibited biofilm formation by *S. mutans* and *S. sanguinis* without host cell toxicity, underscoring their potential as safe metal–peptidomimetic antimicrobial agents.

Overall, this study demonstrates how subtle stereochemical modifications can reprogram the coordination chemistry of histidine-rich scaffolds, yielding metal-peptidomimetic agents with distinct inorganic mechanisms of action. Peptide backbone stereochemistry emerges as an independent design parameter for modulating donor accessibility, coordination modes, metal-ion selectivity, and ultimately the proteolytic stability and biological activity of metal-peptidomimetic therapeutics.

## Supplementary Material



## Data Availability

All data supporting this study are provided in the Supporting Information. Supporting Information will be available at the journal Web site via the article DOI. Additional information may be obtained from the corresponding authors upon reasonable request.

## Notes

Supporting Information includes analytical validation of the synthesized peptide; mass spectrometric and spectroscopic data; pH-dependent speciation, redox, and ROS-generation studies of its Cu­(II) and Zn­(II) complexes; thermodynamic parameters; antimicrobial assay data; and XYZ coordinates of then 12 truncated theoretical models discussed in the publication (PDF)
