# Supplementary material for: Dual Amino Acid Swap in MUC7-Derived Peptide Enhances Resistance and Modulates Zn(II) and Cu(II) Complex Stability, Secondary Structure and Antimicrobial Activity
Source: Inorg Chem. 2026 Mar 4;65(10):5611–26. doi: 10.1021/acs.inorgchem.5c05849 (PMC13298914; doi:10.1021/acs.inorgchem.5c05849)
Supplement: Supplementary file 1 [file ic5c05849_si_001.pdf]

Supporting Information

Dual Amino Acid Swap in MUC7-Derived Peptide Enhances Resistance and Modulates Zn(II) and Cu(II) Complex Stability, Secondary Structure and Antimicrobial Activity

Klaudia Szarszoń[a]†, Jan Kachnowicz[a]†, Tomasz Janek[b], Alicia Domínguez-Martin[c], Aneta Jezierska[a], Joanna Wątły[a*]

[a] Faculty of Chemistry, University of Wrocław, F. Joliot-Curie 14, 50-383 Wrocław, Poland

[b] Department of Biotechnology and Food Microbiology, Wrocław University of Environmental and Life Sciences, Chełmońskiego 37, 51-630, Wrocław, Poland

[c] Department of Inorganic Chemistry, Faculty of Pharmacy, University of Granada, E-18071 Granada, Spain

† Equally contributed

* Correspondence should be addressed to: Joanna Wątły, email: joanna.watly2@uwr.edu.pl

Table of Contents

Figure S 1. Certificates of analysis of synthesized peptide EGRERDHELRHRrhHHQSPK received from KareBay™ Biochem company. 4
Figure S 2. MALDI-TOF MS spectra of (A) EGRERDHELRHRRHHHQSPK and (B) EGRERDHELRHRrhHHQSPK peptides after trypsin digestion. Each sample consists of a peptide, a buffer, and trypsin. The signal at *m/z* 870.398 corresponds to the HHHQSPK fragment; this signal is absent in the peptidomimetic (EGR with two D-amino acids), indicating proteolytic resistance at the modified site in the peptide chain... 4
Figure S 3. Representative distribution diagram for EGRERDHELRHRrhHHQSPK peptide in aqueous solution of 4 mM $HClO_4$ with I = 100 mM $NaClO_4$, dependent on pH values. C_L = 4 mM. 5
Figure S 4. ESI-MS spectra for (A) Cu(II) and (B) Zn(II) complexes with EGR 2D-aa peptide with enlarged chosen *m/z* regions. Comparisons of experimental and simulated spectra are presented for selected signals. Molar ratio M:L – 1:1. [L] = 0.0001 M. Samples prepared in MeOH:H_2O (50:50) mixture. 6
Figure S 5. Representative distribution diagram for the (A) Cu(II) – EGR 2D-aa and (B) Zn(II) – EGR 2D-aa systems in aqueous solution of 4 mM $HClO_4$ with I = 100 mM $NaClO_4$, dependent on pH values. C_L = 0.4 mM; molar ratio M/L–0.9:1. 7
Figure S 6. pH-dependent EPR spectra and obtained parameters for the Cu(II) – EGR 2D-aa system in aqueous solution with the addition of ethylene glycol (30%) at a temperature of 77 K; [Cu(II)] = 1 mM; molar ratio M/L–0.9:1. 7
Figure S 7. Comparison of d-d band in UV-vis spectra at chosen pH values (5.5, 7.0, 7.5) for the Cu(II) – EGR L-aa (dashed line,[1]) and Cu(II) – EGR 2D-aa complexes (continuous line) in aqueous solution of 4 mM $HClO_4$ with I = 100 mM $NaClO_4$. Optical path length of 1 cm. C_L = 0.4 mM; molar ratio M/L–0.9:1.8
Figure S 8. Comparison of CD spectra in the pH ranges (A) 7.0 - 9.0 and (B) 9.5 - 12.5 for the Cu(II) – EGR L-aa (dashed lines, [1] and Cu(II) – EGR 2D-aa complexes (continuous lines). C_L = 0.4 mM; molar ratio M/L–0.9:1; I = 100 mM $NaClO_4$; the optical path length = 1 cm; T = 25 ℃. 9
Figure S 9. Far-UV CD spectra at 180-250 nm of (A) the EGR 2D-aa peptide, and its (B) Cu(II), and (C) Zn(II) complexes at chosen pH values (3.5, 5.5, 7.5, 9.5, and 11.5) in aqueous solution of 4 mM $HClO_4$ with I = 100 mM $NaClO_4$; the optical path length = 0.2 mm; C_L= 0.3 mM. 11
Figure S 10. Far-UV CD spectra at 180–250 nm at (A) pH 5.4, and (B) pH 7.4 for the EGR 2D-aa complexes with Zn(II) and Cu(II) in aqueous solution of 4 mM $HClO_4$ with I = 100 mM $NaClO_4$; molar ratio M/L 0.9:1; the optical path length = 0.2 mm; C_L =0.3 mM. 11
Figure S 11. UV-visible spectra showing ascorbate consumption by (A) Cu(II) (blue line); (B) Cu(II) – EGR 2D-aa complex (green line) and (C) EGR 2D-aa peptide (orange line) in phosphate buffer (pH 5.4) as a function of time. λ = 265 nm; [Asc] = 100 µM; [Cu(II)] = 8 µM; [EGR 2D-aa] =10 µM. 12
Figure S 12. Fluorescence curves of ROS production. Fluorescence of the 7-OH-CCA (λ_{exc} = 395 nm, λ_{em} = 452 nm) as a function of time, showing the scavenging kinetics of HO· produced by Cu(II) (50 µM), ascorbate (200 µM) and peptide (60 µM) in phosphate-buffered solution (50mM, pH 5.4) with CCA (200 µM). The comparison of curves were presented: (A) control sample with Cu(II), CCA and Asc (red curve); control sample with peptide EGR 2D-aa, Cu(II) and CCA (black curve); sample with peptide EGR 2D-aa, Cu(II), CCA and Asc (blue curve); (B) control sample with Cu(II), CCA and Asc (red curve); sample with peptide EGR 2D-aa, Cu(II) and CCA (black curve); (C) control sample with peptide EGR 2D-aa, Cu(II) and CCA (back curve); sample with peptide EGR 2D-aa, Cu(II), CCA and Asc (blue curve). 12

Table S 1. *m/z* values (for monoisotopic masses) for individual ions of the complex forms and ligands (with the highest intensity) obtained by ESI-MS spectrometry for the studied peptides with Cu(II) and Zn(II) ions. 5
Table S 2. Thermodynamic and spectroscopic data for proton and the Cu(II) – EGR 2D-aa in aqueous solution of 4 mM $HClO_4$ for each calculated complex species with the proposed coordination modes, C_L = 0.4 mM; molar ratio M/L–0.9:1; I = 100 mM $NaClO_4$; T = 25 ℃. 9
Table S 3. The antibacterial and anti-*Candida* activities of peptidomimetic/complexes were assessed *in vitro* by determining their MIC (µg/mL). Antimicrobial tests were conducted in a 10 mM MES buffer at

pH 5.4. Experiments were performed for the peptide and its metal ion complexes. *n/d*, not determined within the concentration range used in this study..12
Table S 4. The antibacterial and anti-*Candida* activities of peptidomimetic/complexes were assessed *in vitro* by determining their MIC (µg/mL). Antimicrobial tests were conducted in a 10 mM HEPES buffer at pH 7.4. Experiments were performed for the peptide and its metal ion complexes. *n/d,* not determined within the concentration range used in this study. 13

XYZ files of 12 short models presented in the publication...13

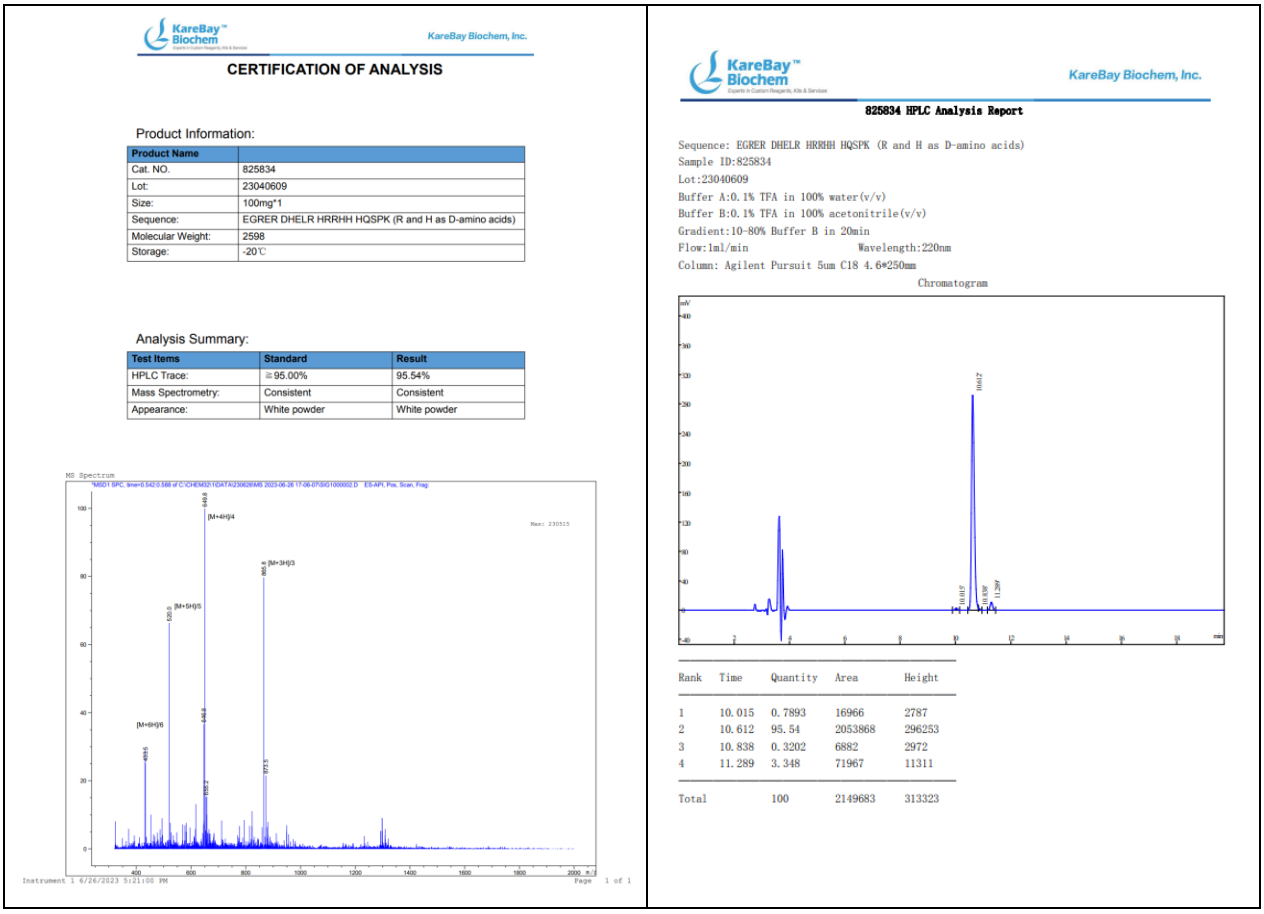

KareBay™ Biochem

KareBay Biochem, Inc.

CERTIFICATION OF ANALYSIS

Product Information:

Product Name	
Cat. NO.	825834
Lot:	23040609
Size:	100mg*1
Sequence:	EGRER DHELR HRRHH HQSPK (R and H as D-amino acids)
Molecular Weight:	2598
Storage:	-20℃

Analysis Summary:

Test Items	Standard	Result
HPLC Trace:	≥95.00%	95.54%
Mass Spectrometry:	Consistent	Consistent
Appearance:	White powder	White powder

MS Spectrum

Instrument 1 6/26/2023 5:21:00 PM Page 1 of 1

KareBay™ Biochem

KareBay Biochem, Inc.

825834 HPLC Analysis Report

Sequence: EGRER DHELR HRRHH HQSPK (R and H as D-amino acids)
Sample ID:825834
Lot:23040609
Buffer A:0.1% TFA in 100% water(v/v)
Buffer B:0.1% TFA in 100% acetonitrile(v/v)
Gradient:10-80% Buffer B in 20min
Flow:1ml/min Wavelength:220nm
Column: Agilent Pursuit 5um C18 4.6*250mm

Chromatogram

Rank	Time	Quantity	Area	Height
1	10.015	0.7893	16966	2787
2	10.612	95.54	2053868	296253
3	10.838	0.3202	6882	2972
4	11.289	3.348	71967	11311
Total		100	2149683	313323

Figure S 1. Certificates of analysis of synthesized peptide EGRERDHELRHRrhHHQSPK received from KareBay™ Biochem company.

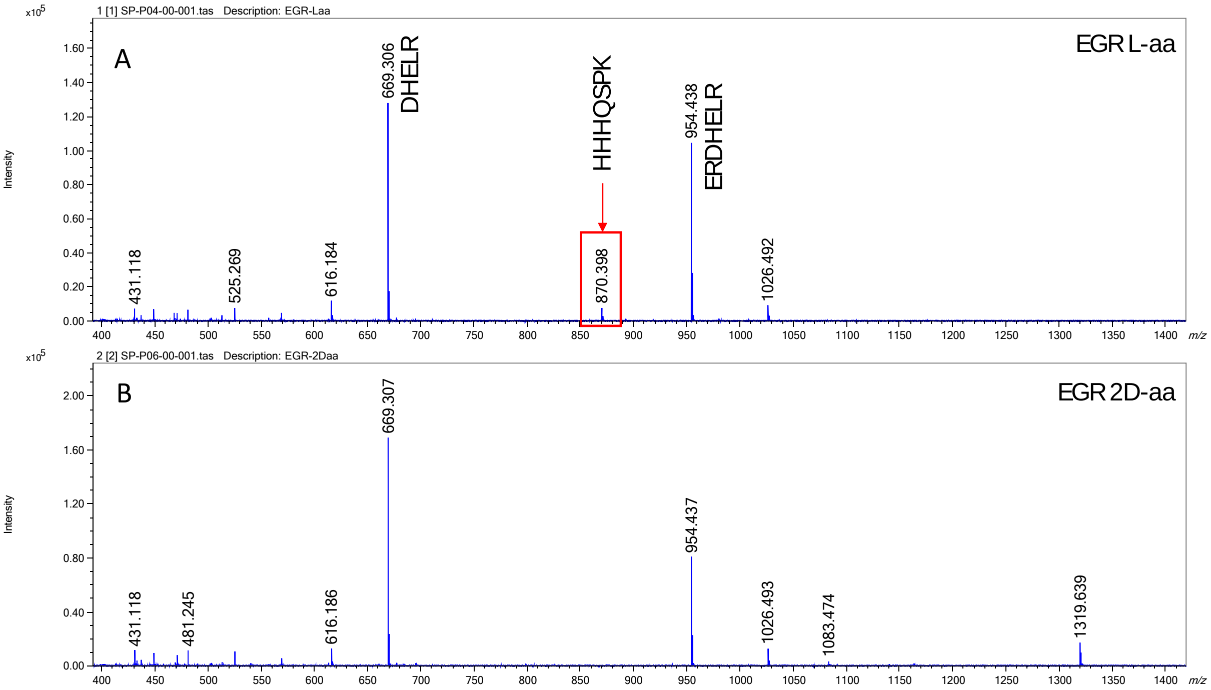

Figure S 2. MALDI-TOF MS spectra of (A) EGRERDHELRHRRHHHQSPK and (B) EGRERDHELRHRrhHHQSPK peptides after trypsin digestion. Each sample consists of a peptide, a buffer, and trypsin. The signal at m/z 870.398 corresponds to the HHHQSPK fragment; this signal is absent in the peptidomimetic (EGR with two D-amino acids), indicating proteolytic resistance at the modified site in the peptide chain.

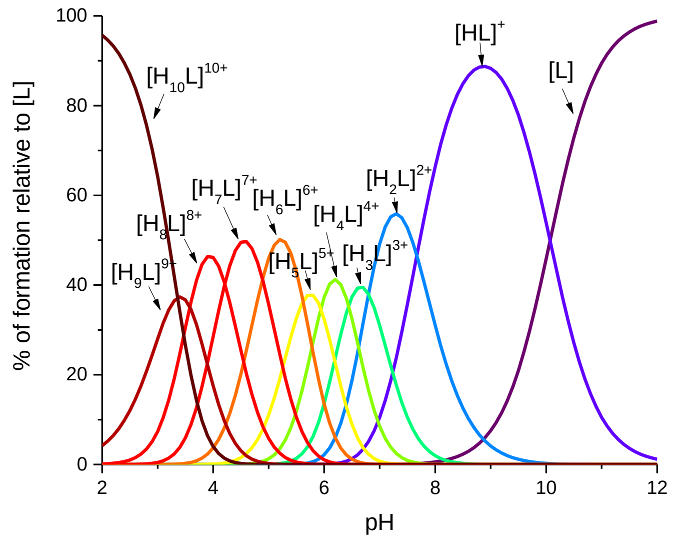

Figure S 3. Representative distribution diagram for EGRERDHELRHRrhHHQSPK peptide in aqueous solution of 4 mM $HClO_4$ with I = 100 mM $NaClO_4$, dependent on pH values. C_L = 4 mM.

Table S 1. m/z values (for monoisotopic masses) for individual ions of the complex forms and ligands (with the highest intensity) obtained by ESI-MS spectrometry for the studied peptides with Cu(II) and Zn(II) ions.

**Cu(II) – EGR 2D-aa**	
**Form**	**Monoisotopic mass *m/z***
**$[L]^{3+}$**	866.444
**$[CuL]^{3+}$**	886.748
**$[L]^{4+}$**	650.085
**$[CuL]^{4+}$**	665.313
**$[L+ClO_4^-]^{4+}$**	675.074
**$[CuL+ClO_4^-]^{4+}$**	690.302
**$[CuL+ K^+ +ClO_4^-]^{4+}$**	699.791
**$[L]^{5+}$**	520.269
**$[CuL]^{5+}$**	532.452
**$[L+ClO_4^-]^{5+}$**	540.260
**$[CuL+ClO_4^-]^{5+}$**	552.443
**$[L]^{6+}$**	433.725
**$[CuL]^{6+}$**	443.878
**$[L+ClO_4^-]^{6+}$**	450.385
**$[CuL+ClO_4^-]^{6+}$**	460.537
**$[L]^{7+}$**	371.909
**Zn(II) – EGR 2D-aa**	
**Form**	**Monoisotopic mass *m/z***
**$[L]^{3+}$**	866.444
**$[ZnL]^{3+}$**	887.082
**$[L+ClO_4^-]^{3+}$**	899.762
**$[ZnL+ClO_4^-]^{3+}$**	920.400
**$[L]^{4+}$**	650.085
**$[ZnL]^{4+}$**	665.563

**$[L+ClO_4^-]^{4+}$**	675.074
**$[ZnL+ClO_4^-]^{4+}$**	690.552
**$[L]^{5+}$**	520.269
**$[L+ClO_4^-]^{5+}$**	540.260

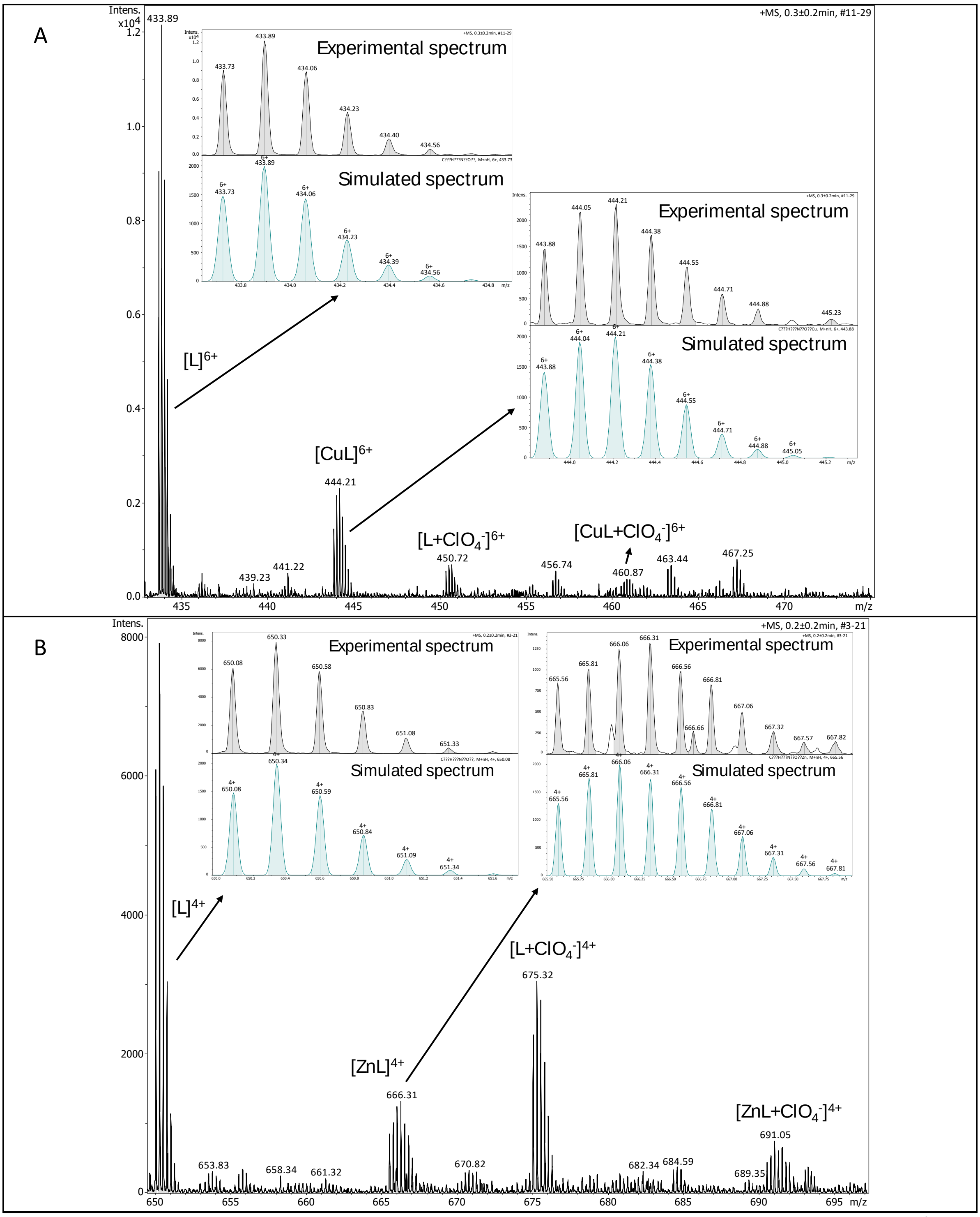

Figure S 4. ESI-MS spectra for (A) Cu(II) and (B) Zn(II) complexes with EGR 2D-aa peptide with enlarged chosen m/z regions. Comparisons of experimental and simulated spectra are presented for selected signals. Molar ratio M:L – 1:1. [L] = 0.0001 M. Samples prepared in MeOH:H_2O (50:50) mixture.

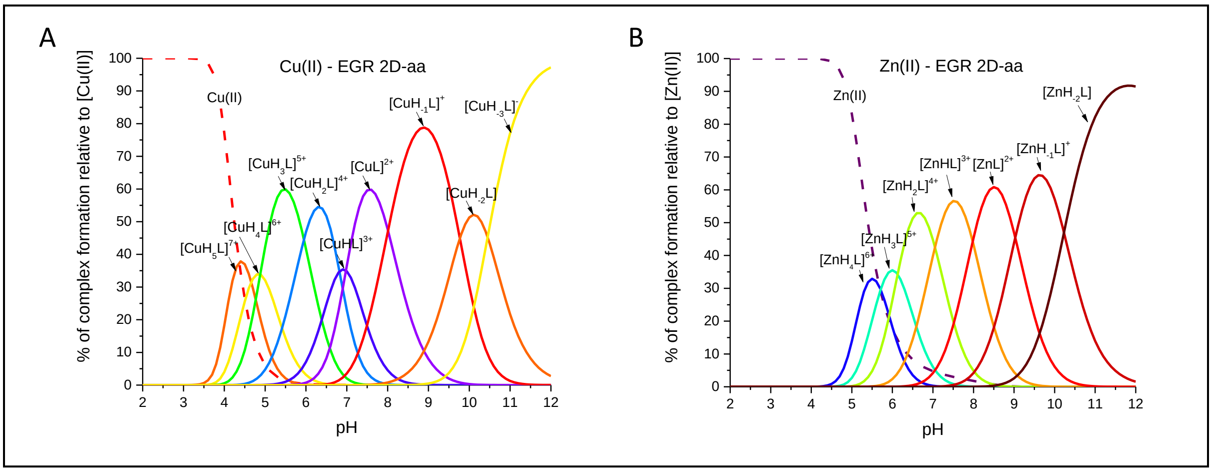

Figure S 5. Representative distribution diagram for the (A) Cu(II) – EGR 2D-aa and (B) Zn(II) – EGR 2D-aa systems in aqueous solution of 4 mM $HClO_4$ with I = 100 mM $NaClO_4$, dependent on pH values. C_L = 0.4 mM; molar ratio M/L–0.9:1.

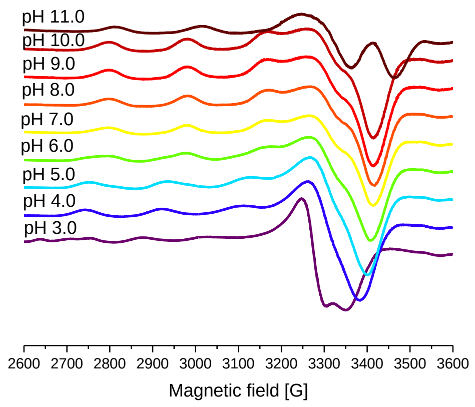

pH	A‖ [G] (A_{zz})	g‖ (g_{zz})	MW Frequency [GHz]	Suggested coordination mode
3.0	119/141	2.31/2.42	9.52606	4O/2N
4.0	177	2.26	9.52266	2N
5.0	178/184	2.25/2.22	9.526545	2N/3N
6.0	178/184	2.25/2.22	9.52602	2N/3N
7.0	185	2.22	9.52603	3N/4N
8.0	185	2.21	9.52621	4N
9.0	181	2.21	9.52123	4N
10.0	181	2.21	9.52708	4N
11.0	201	2.19	9.52635	4N

Figure S 6. pH-dependent EPR spectra and obtained parameters for the Cu(II) – EGR 2D-aa system in aqueous solution with the addition of ethylene glycol (30%) at a temperature of 77 K; [Cu(II)] = 1 mM; molar ratio M/L–0.9:1.

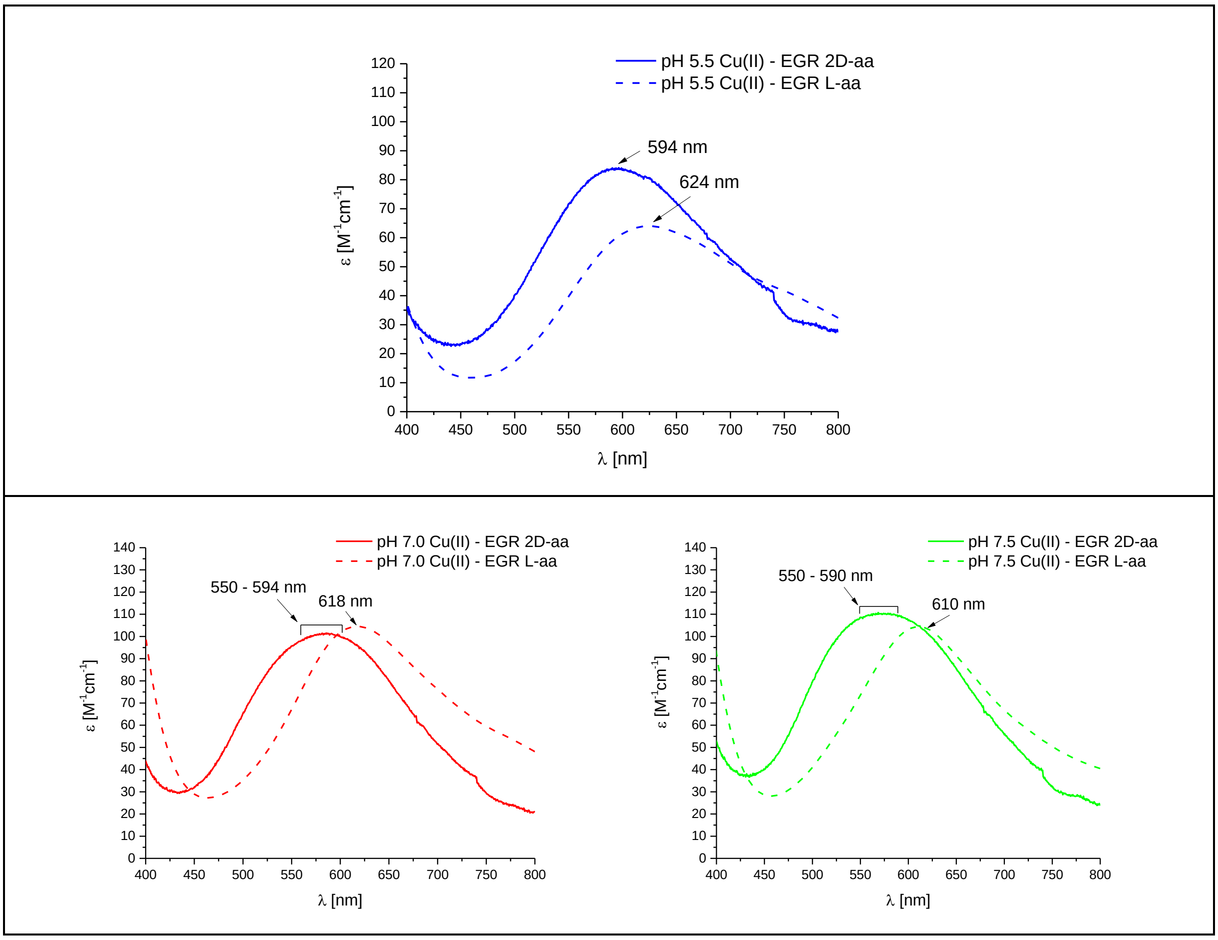

Figure S 7. Comparison of d-d band in UV-vis spectra at chosen pH values (5.5, 7.0, 7.5) for the Cu(II) – EGR L-aa (dashed line,[1]) and Cu(II) – EGR 2D-aa complexes (continuous line) in aqueous solution of 4 mM $HClO_4$ with I = 100 mM $NaClO_4$. Optical path length of 1 cm. C_L = 0.4 mM; molar ratio M/L–0.9:1.

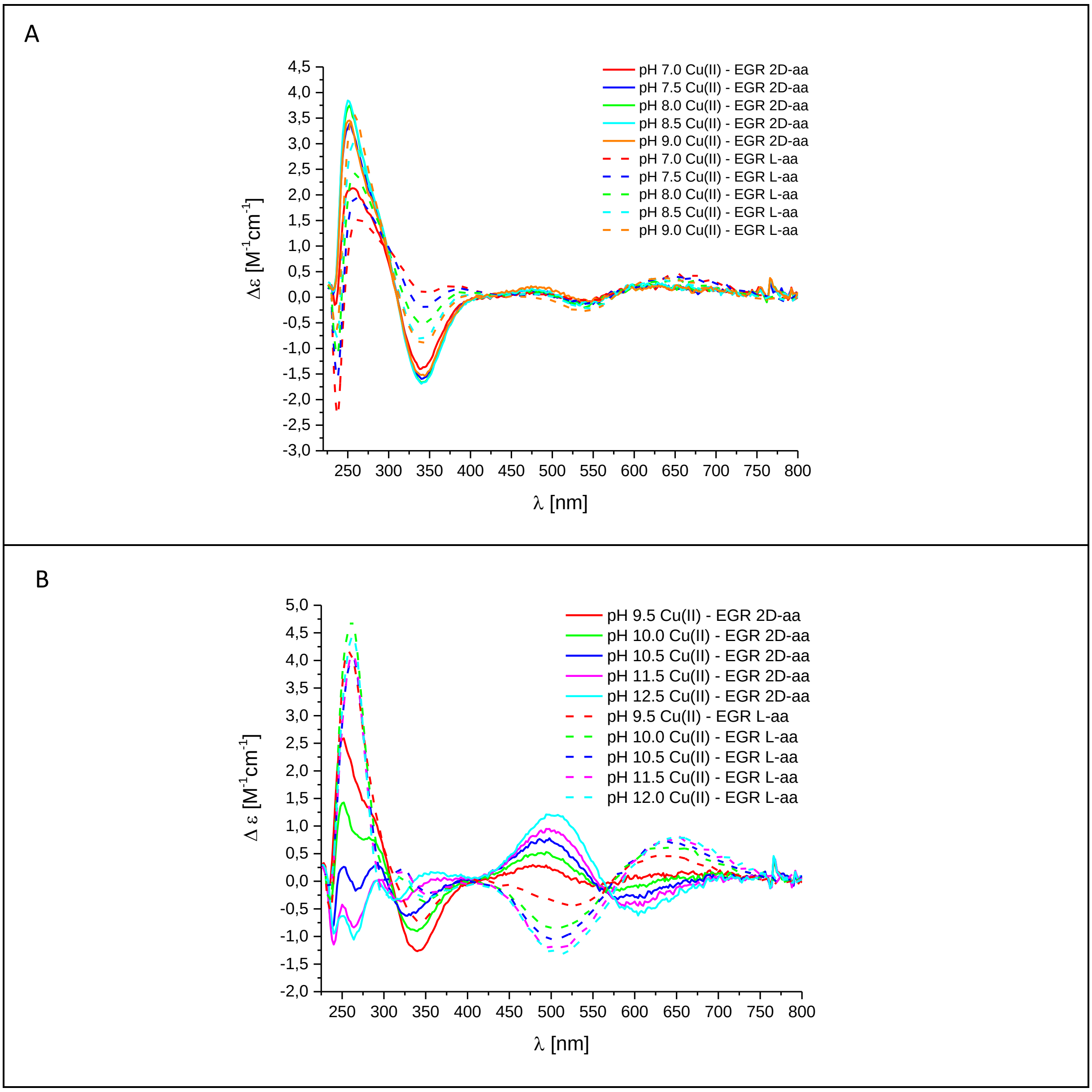

Figure S 8. Comparison of CD spectra in the pH ranges (A) 7.0 - 9.0 and (B) 9.5 - 12.5 for the Cu(II) – EGR L-aa (dashed lines, [1] and Cu(II) – EGR 2D-aa complexes (continuous lines). C_L = 0.4 mM; molar ratio M/L–0.9:1; I = 100 mM $NaClO_4$; the optical path length = 1 cm; T = 25 ˚C.

Table S 2. Thermodynamic and spectroscopic data for proton and the Cu(II) – EGR 2D-aa in aqueous solution of 4 mM $HClO_4$ for each calculated complex species with the proposed coordination modes, C_L = 0.4 mM; molar ratio M/L–0.9:1; I = 100 mM $NaClO_4$; T = 25 ˚C.

**Complex species**	**Complex species**			**UV-vis**		**CD**		**EPR**		**Coordination mode**
	logβ	pK_a	pH	nm	ε[M-1cm-1]	nm	Δ ε [M-1cm-1]	A‖ [G]	g‖	
**[CuH_5L]$^{7+}$**	42.46(1)		4.4	623	47.05	238	-3.92	177	2.26	2N {2N$_{im}$}
**[CuH_4L]$^{6+}$**	37.8(2)	4.66	4.8	604	71.71	238<br>286<br>338	-2.61<br>0.19<br>-0.31	178/184	2.25/2.22	3N {3N$_{im}$} polymorphic
**[CuH_3L]$^{5+}$**	32.92(1)	4.88	5.5	590	83.36	240<br>284<br>338	-2.37<br>0.31<br>-0.51	178/184	2.25/2.22	3N {3N$_{im}$} polymorphic

**$[CuH_2L]^{4+}$**	26.97(1)	5.95	6.3	589	94.44	240<br>280<br>340	-1.78<br>0.64<br>-0.77	178/184	2.25/2.22	3N<br>{3N_{im}}<br>polymorphic
**$[CuHL]^{3+}$**	20.15(2)	6.82	6.9	580	100.98	234<br>254<br>342	-0.15<br>2.12<br>-1.37	185	2.22	4N<br>{3N_{im}+N_{am} }
**$[CuL]^{2+}$**	13.17(1)	6.98	7.6	569	109.99	254<br>340<br>540<br>624	3.37<br>-1.60<br>-0.11<br>0,24	185	2.22	4N<br>{3N_{im}+N_{am} }<br>deprotonation of non-bonding N-terminus
**$[CuH_{-1}L]^{+}$**	5.16(2)	8.01	8.9	557	114.50	252<br>342<br>480<br>546<br>624	3.45<br>-1.51<br>0.21<br>-0.09<br>0,20	185	2.21	4N<br>{2N_{im} + 2N_{am}}
**$[CuH_{-2}L]$**	-4.61(2)	9.77	10.1	551	118.84	252<br>286<br>338<br>490<br>586	1.43<br>0.74<br>-0.89<br>0.50<br>-0.15	185	2.21	4N<br>{N_{im} + 3N_{am}}
**$[CuH_{-3}L]^{-}$**	-15.06 (2)	10.45								4N<br>{N_{im} + 3N_{am}},<br>deprotonation of non-bonding Lys

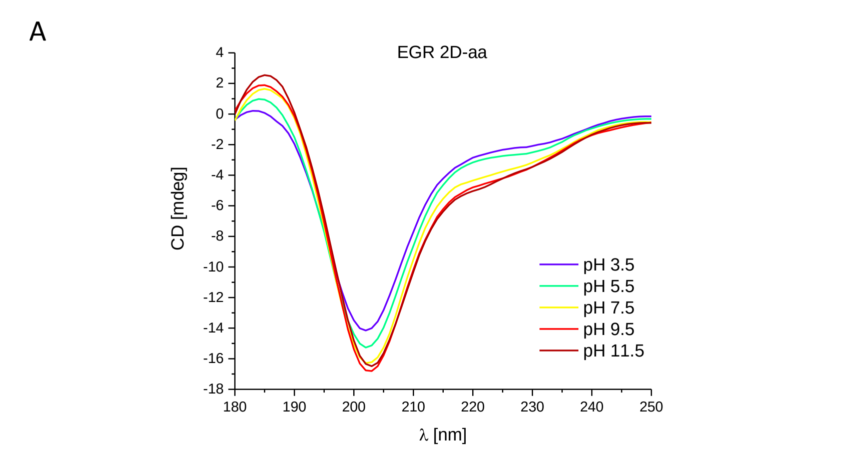

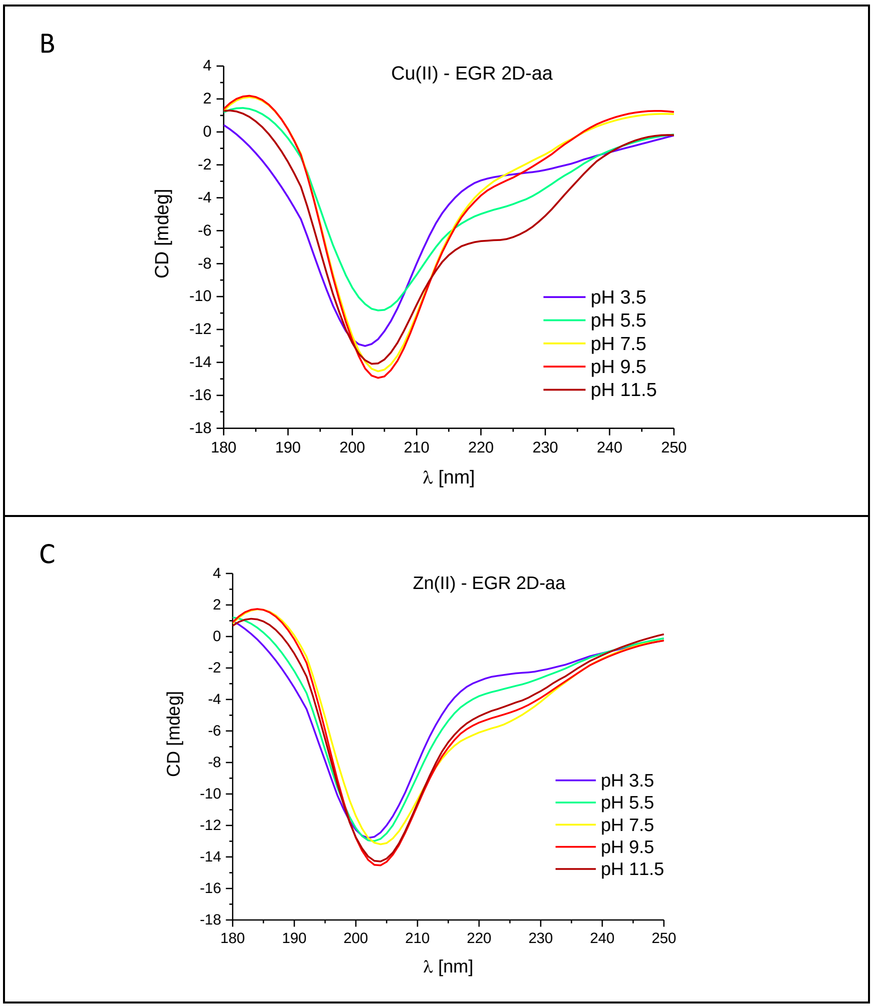

Figure S 9. Far-UV CD spectra at 180-250 nm of (A) the EGR 2D-aa peptide, and its (B) Cu(II), and (C) Zn(II) complexes at chosen pH values (3.5, 5.5, 7.5, 9.5, and 11.5) in aqueous solution of 4 mM $HClO_4$ with I = 100 mM $NaClO_4$; the optical path length = 0.2 mm; C_L= 0.3 mM.

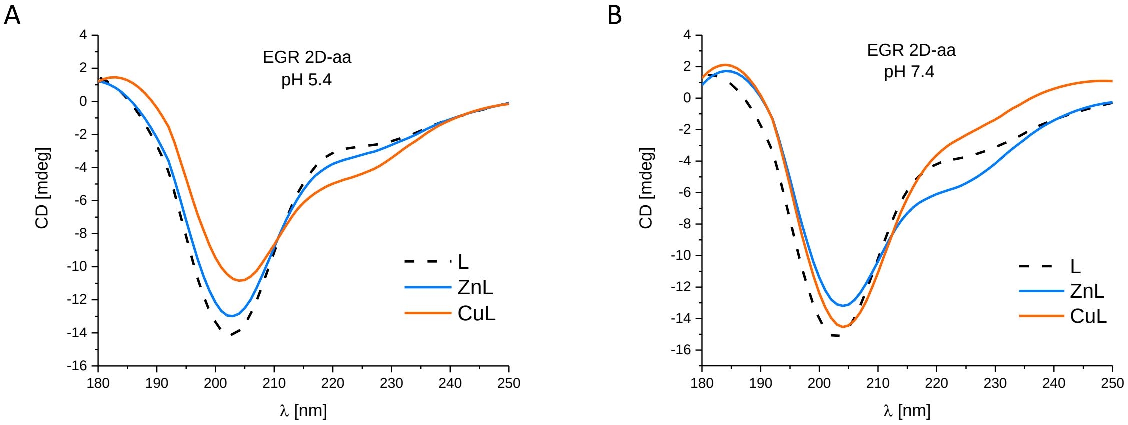

Figure S 10. Far-UV CD spectra at 180–250 nm at (A) pH 5.4, and (B) pH 7.4 for the EGR 2D-aa complexes with Zn(II) and Cu(II) in aqueous solution of 4 mM $HClO_4$ with I = 100 mM $NaClO_4$; molar ratio M/L 0.9:1; the optical path length = 0.2 mm; C_L =0.3 mM.

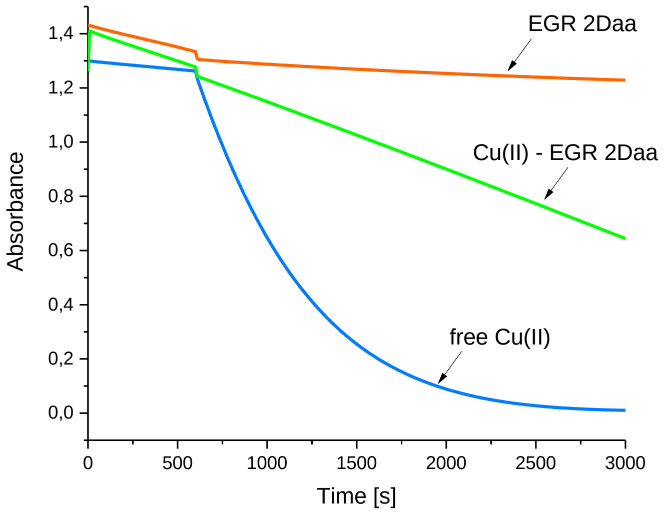

Figure S 11. UV-visible spectra showing ascorbate consumption by (A) Cu(II) (blue line); (B) Cu(II) – EGR 2D-aa complex (green line) and (C) EGR 2D-aa peptide (orange line) in phosphate buffer (pH 5.4) as a function of time. λ = 265 nm; [Asc] = 100 µM; [Cu(II)] = 8 µM; [EGR 2D-aa] =10 µM.

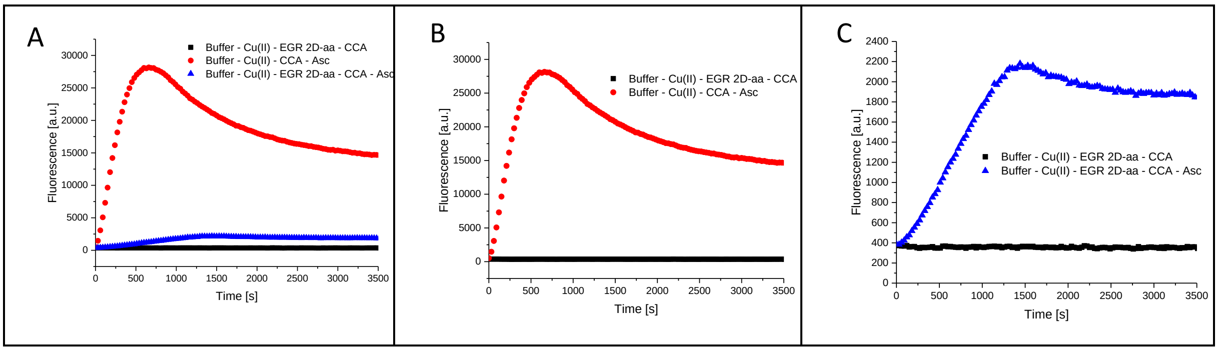

Figure S 12. Fluorescence curves of ROS production. Fluorescence of the 7-OH-CCA (λ_{exc} = 395 nm, λ_{em} = 452 nm) as a function of time, showing the scavenging kinetics of HO· produced by Cu(II) (50 µM), ascorbate (200 µM) and peptide (60 µM) in phosphate-buffered solution (50mM, pH 5.4) with CCA (200 µM). The comparison of curves were presented: (A) control sample with Cu(II), CCA and Asc (red curve); control sample with peptide EGR 2D-aa, Cu(II) and CCA (black curve); sample with peptide EGR 2D-aa, Cu(II), CCA and Asc (blue curve); (B) control sample with Cu(II), CCA and Asc (red curve); sample with peptide EGR 2D-aa, Cu(II) and CCA (black curve); (C) control sample with peptide EGR 2D-aa, Cu(II) and CCA (back curve); sample with peptide EGR 2D-aa, Cu(II), CCA and Asc (blue curve).

Table S 3. The antibacterial and anti-Candida activities of peptidomimetic/complexes were assessed in vitro by determining their MIC (µg/mL). Antimicrobial tests were conducted in a 10 mM MES buffer at pH 5.4. Experiments were performed for the peptide and its metal ion complexes. n/d, not determined within the concentration range used in this study.

**Strain**	**EGR 2D-aa**	**+Cu(II)**	**+Zn(II)**
E. coli ATCC 25922	n/d	n/d	n/d
P. aeruginosa ATCC 15442	n/d	n/d	n/d
E. faecalis ATCC 29212	n/d	n/d	n/d
S. aureus ATCC 25923	n/d	n/d	n/d
S. mutans PCM 2502	n/d	**500**	**250**
S. sanguinis PMC 2335	n/d	**250**	**125**
C. albicans SC5314	n/d	n/d	n/d

Table S 4. The antibacterial and anti-Candida activities of peptidomimetic/complexes were assessed in vitro by determining their MIC (μg/mL). Antimicrobial tests were conducted in a 10 mM HEPES buffer at pH 7.4. Experiments were performed for the peptide and its metal ion complexes. n/d, not determined within the concentration range used in this study.

Strain	EGR 2D-aa	+Cu(II)	+Zn(II)
E. coli ATCC 25922	n/d	n/d	n/d
P. aeruginosa ATCC 15442	n/d	n/d	n/d
E. faecalis ATCC 29212	n/d	n/d	n/d
S. aureus ATCC 25923	n/d	n/d	n/d
S. mutans PCM 2502	n/d	**500**	**500**
S. sanguinis PMC 2335	n/d	**250**	**250**
C. albicans SC5314	n/d	n/d	n/d

Table S 5. The antibacterial and anti-Candida activities of copper (Cu) and zinc (Zn) metal ions were assessed in vitro by determining their MIC (μg/mL). Antimicrobial tests were conducted in a 10 mM MES buffer at pH 5.4. n/d, not determined within the concentration range used in this study.

Strain	Cu(II)	Zn(II)
E. coli ATCC 25922	n/d	n/d
P. aeruginosa ATCC 15442	n/d	n/d
E. faecalis ATCC 29212	n/d	n/d
S. aureus ATCC 25923	n/d	n/d
S. mutans PCM 2502	n/d	n/d
S. sanguinis PMC 2335	n/d	n/d
C. albicans SC5314	n/d	n/d

Experiments were performed with copper (Cu(II)) and zinc (Zn(II)) ions at concentrations ranging from 0.3 to 38 μg/mL, corresponding to the metal ion concentrations present in the respective complexes.

Table S 6. The antibacterial and anti-Candida activities of copper (Cu) and zinc (Zn) metal ions were assessed in vitro by determining their MIC (μg/mL). Antimicrobial tests were conducted in a 10 mM HEPES buffer at pH 7.4. n/d, not determined within the concentration range used in this study.

Strain	Cu(II)	Zn(II)
E. coli ATCC 25922	n/d	n/d
P. aeruginosa ATCC 15442	n/d	n/d
E. faecalis ATCC 29212	n/d	n/d
S. aureus ATCC 25923	n/d	n/d
S. mutans PCM 2502	n/d	n/d
S. sanguinis PMC 2335	n/d	n/d
C. albicans SC5314	n/d	n/d

Experiments were performed with copper (Cu(II)) and zinc (Zn(II)) ions at concentrations ranging from 0.3 to 38 μg/mL, corresponding to the metal ion concentrations present in the respective complexes.

XYZ files of 22 short models presented in the publication.

202

$[CuH_3L]^{5+}$ with {His-7, His-11, His-15} coordination

N 12.58378 5.88308 -10.75499

C 13.43535 5.00250 -11.53468

H 14.44405 5.43052 -11.60147

C	13.50918	3.61508	-10.85479
H	13.78942	3.75853	-9.80487
H	12.52207	3.13975	-10.87177
C	14.52194	2.76122	-11.53410
N	15.77968	2.53297	-11.01223
H	16.08856	2.78964	-10.08321
C	16.52583	1.89236	-11.92753
H	17.55137	1.58614	-11.77051
N	15.80443	1.68880	-13.03403
C	14.54808	2.21681	-12.78901
H	13.77008	2.20094	-13.53891
C	12.93984	4.88721	-12.99110
O	13.71108	5.04455	-13.94769
N	11.64082	4.58940	-13.13341
H	11.05603	4.49131	-12.30365
C	10.99303	4.41015	-14.43200
H	11.36692	3.50028	-14.92121
C	9.48093	4.32572	-14.17293
H	9.29504	3.45084	-13.53703
H	9.19552	5.21131	-13.59703
C	8.59332	4.26205	-15.40957
H	8.78923	5.12807	-16.05965
H	8.78529	3.35939	-15.99891
C	7.09804	4.31749	-15.02678
O	6.31096	3.53792	-15.61908
O	6.76342	5.16696	-14.14033
C	11.30425	5.55974	-15.39059
O	11.67297	5.34968	-16.55575
N	11.09274	6.79853	-14.91174
H	10.76903	6.92434	-13.95320
C	11.20884	7.96072	-15.77541
C	10.55704	9.18174	-15.11823

C	9.03309	9.06362	-14.95739
C	8.31778	9.00729	-16.30800
C	8.52418	10.24003	-14.12160
C	12.64775	8.26147	-16.18543
O	12.85646	9.00850	-17.16395
N	13.64009	7.70455	-15.48058
H	13.42274	7.06638	-14.71862
C	15.02759	7.83538	-15.89722
H	15.18160	8.86208	-16.25979
C	15.97850	7.59050	-14.72265
H	15.77855	6.59684	-14.29876
H	17.00251	7.58364	-15.11349
C	15.81661	8.67370	-13.65608
H	16.13892	9.63861	-14.06992
H	14.76215	8.76824	-13.37952
C	16.60235	8.34434	-12.38506
H	16.35754	7.32691	-12.06032
H	17.68517	8.35996	-12.57266
N	16.25776	9.21728	-11.26346
H	15.61024	8.85635	-10.54201
C	16.57859	10.50821	-11.17576
N	17.44535	11.06762	-12.04006
H	18.05941	10.48416	-12.58664
H	17.75793	12.01221	-11.87707
N	16.02243	11.24532	-10.20424
H	15.13223	10.91329	-9.78611
H	16.22506	12.23231	-10.17195
C	15.36122	6.95278	-17.10216
O	16.41783	7.12889	-17.71882
N	14.46654	6.02809	-17.49934
H	13.56996	5.91859	-17.02732
C	14.68329	5.34011	-18.76322

H	15.73288	5.03184	-18.81722
C	13.80553	4.07744	-18.84783
H	12.76240	4.33127	-18.62842
H	13.84637	3.69118	-19.87123
C	14.32957	3.04335	-17.90362
N	14.81489	1.82024	-18.32298
H	14.75728	1.45253	-19.26388
C	15.37244	1.18367	-17.27422
H	15.84099	0.21080	-17.33460
N	15.26043	1.93870	-16.18551
C	14.60850	3.09853	-16.56340
H	14.39785	3.88101	-15.84792
C	14.50054	6.29514	-19.96027
O	15.17258	6.15005	-20.98329
N	13.58320	7.27171	-19.80502
H	13.07312	7.33911	-18.93291
C	13.38930	8.33610	-20.78242
H	13.49753	7.90179	-21.78249
C	11.96001	8.88925	-20.61105
H	11.32479	8.01516	-20.42652
H	11.90804	9.50215	-19.70151
C	11.36982	9.62170	-21.82205
H	11.62054	9.07515	-22.73758
H	10.27634	9.58799	-21.73741
C	11.77685	11.07553	-22.02736
H	12.86707	11.16998	-22.11901
H	11.32665	11.43498	-22.96280
N	11.30099	11.89472	-20.90562
H	10.73404	11.44337	-20.20150
C	11.37779	13.22743	-20.86591
N	11.97463	13.90033	-21.85604
H	12.49332	13.41371	-22.56953

H	12.16015	14.88593	-21.75073
N	10.83178	13.89819	-19.84446
H	10.42601	13.40874	-19.06266
H	10.94020	14.89705	-19.77060
C	14.50112	9.40940	-20.66802
O	15.02307	9.88264	-21.68977
N	14.81645	9.77062	-19.41331
H	14.28352	9.33714	-18.66319
C	15.84598	10.70286	-18.93915
H	15.52357	10.96800	-17.92753
C	16.03047	11.98778	-19.76261
H	16.80513	12.57507	-19.24927
H	16.42921	11.71578	-20.74210
C	14.78243	12.84929	-19.94705
H	14.99799	13.59706	-20.71844
H	13.95627	12.23926	-20.33115
C	14.31965	13.60982	-18.70550
H	15.18244	14.09369	-18.23530
H	13.60714	14.39431	-18.99176
N	13.69469	12.70506	-17.73713
H	13.09377	11.98095	-18.11049
C	13.89482	12.70419	-16.41618
N	14.50722	13.72913	-15.81252
H	14.53099	14.63905	-16.24554
H	14.67762	13.68912	-14.81942
N	13.49345	11.64526	-15.70480
H	13.28470	10.77309	-16.17904
H	13.55864	11.64503	-14.69890
C	17.23040	10.04157	-18.83488
O	17.82898	9.64734	-19.84360
N	17.73520	9.99895	-17.58799
H	17.22102	10.48807	-16.86236

C	19.15935	9.85600	-17.27294
H	19.20420	9.53613	-16.22060
C	19.86322	11.21285	-17.39708
H	20.94638	11.06667	-17.36675
H	19.62153	11.63429	-18.38161
C	19.46415	12.16047	-16.30808
N	18.15696	12.29975	-15.86878
C	18.19188	13.22674	-14.92412
H	17.34594	13.58779	-14.35295
N	19.45250	13.68637	-14.73438
H	19.73711	14.38938	-14.06736
C	20.27930	13.01119	-15.60352
H	21.34346	13.19287	-15.64846
C	19.87165	8.73788	-18.05009
O	21.01388	8.87991	-18.48620
N	19.21097	7.55746	-18.10919
H	18.21859	7.53078	-17.87138
C	19.81531	6.39955	-18.75520
H	20.76504	6.15185	-18.26376
C	18.85687	5.20200	-18.70972
H	17.91452	5.50764	-19.18462
H	19.27951	4.40737	-19.33323
C	18.55435	4.62506	-17.36772
N	18.74389	5.25160	-16.15385
H	19.15902	6.16372	-16.00997
C	18.27203	4.45791	-15.17357
H	18.28835	4.71689	-14.12338
N	17.79561	3.33171	-15.70070
C	17.96691	3.42658	-17.06823
H	17.66271	2.64013	-17.74422
C	20.14047	6.59837	-20.24554
O	21.01607	5.91608	-20.78056

N	19.33673	7.46049	-20.89949
H	18.68149	8.03864	-20.37355
C	19.34961	7.59971	-22.33970
H	20.26695	7.11002	-22.69514
C	18.14256	6.91242	-22.99617
H	17.21559	7.20127	-22.49185
H	18.08471	7.24155	-24.03826
C	18.30407	5.43856	-22.94550
N	19.29686	4.80042	-23.67189
H	19.95950	5.28758	-24.29007
C	19.27466	3.49372	-23.41674
H	19.92760	2.74622	-23.84315
N	18.29107	3.27398	-22.54365
H	18.04369	2.36411	-22.17695
C	17.67092	4.46689	-22.22737
H	16.84116	4.53579	-21.53856
C	19.49324	9.05821	-22.78750
O	18.75796	9.56059	-23.63808
N	20.55480	9.69916	-22.24068
Cu	16.61866	2.12873	-14.73634
H	10.70651	7.74257	-16.72815
H	10.78394	10.06040	-15.73528
H	11.02493	9.34179	-14.13629
H	8.55667	9.89391	-16.90921
H	7.23244	8.97910	-16.16299
H	8.59244	8.11905	-16.88743
H	8.73708	11.19223	-14.62411
H	9.00428	10.25972	-13.13682
H	7.44132	10.17172	-13.97182
H	8.81080	8.14013	-14.40571
C	11.79890	5.83767	-9.85935
H	12.02826	6.32571	-11.48702

O	11.65133	5.28063	-8.77809
C	10.99751	6.45797	-10.05437
H	11.13945	6.93730	-11.00041
H	10.92509	7.19931	-9.28622
H	10.09709	5.88042	-10.07868
H	20.73143	10.64080	-22.46730
H	21.14931	9.22312	-21.61683

$[CuH_3L]^{5+}$ with {His-11, D-His-14, His-15} coordination

202

C	12.40830	7.30220	-10.48358
O	13.05683	7.84329	-11.38719
N	12.43245	5.96988	-10.28380
H	11.90348	5.54945	-9.52262
C	13.18473	5.11902	-11.18865
H	14.23870	5.42536	-11.20945
C	13.09267	3.65218	-10.71391
H	13.61187	3.56081	-9.75502
H	12.04063	3.38263	-10.56056
C	13.70452	2.72574	-11.70288
N	12.96768	2.10070	-12.69487
H	11.96112	2.15438	-12.80318
C	13.77262	1.39375	-13.48855
H	13.47230	0.79444	-14.33578
N	15.01356	1.55181	-13.03138
H	15.83290	1.11982	-13.43991
C	15.00380	2.37226	-11.92369
H	15.90527	2.63903	-11.39410
C	12.70180	5.25045	-12.64050
O	13.52378	5.30064	-13.56775

N	11.37640	5.25172	-12.83850
H	10.73252	5.17458	-12.05180
C	10.84662	5.37425	-14.18934
H	11.30487	4.60226	-14.82232
C	9.32519	5.18408	-14.20103
H	9.12012	4.20921	-13.74170
H	8.84959	5.95259	-13.58061
C	8.73955	5.21522	-15.61457
H	9.33984	4.60760	-16.30091
H	7.74150	4.75638	-15.58518
C	8.52723	6.60740	-16.22556
O	8.58498	6.70691	-17.48531
O	8.24696	7.55541	-15.43507
C	11.27856	6.69695	-14.82277
O	11.63024	6.72138	-16.00999
N	11.26564	7.79771	-14.05632
H	10.91724	7.75653	-13.10107
C	11.66269	9.07815	-14.62226
C	11.38004	10.21531	-13.63285
C	9.89088	10.57014	-13.50663
C	9.38491	11.29592	-14.75511
C	9.66992	11.42404	-12.25682
C	13.13653	9.09274	-15.02329
O	13.51288	9.73933	-16.02033
N	13.99182	8.42698	-14.23661
H	13.63814	7.92439	-13.42561
C	15.40666	8.33290	-14.57850
H	15.80778	9.33728	-14.75933
C	16.19069	7.66419	-13.44601
H	15.73331	6.69014	-13.22282
H	17.20813	7.47042	-13.80783
C	16.23590	8.52019	-12.18122

H	16.76610	9.45919	-12.38464
H	15.21980	8.77665	-11.86362
C	16.91222	7.75953	-11.03791
H	16.45588	6.76795	-10.93978
H	17.97610	7.58996	-11.24810
N	16.74799	8.41076	-9.74270
H	15.90166	8.20734	-9.19928
C	17.42991	9.47316	-9.31304
N	18.45650	9.98247	-10.00763
H	18.83693	9.48724	-10.79778
H	19.05020	10.67533	-9.57860
N	17.07409	10.01667	-8.13655
H	16.16117	9.73314	-7.77368
H	17.43416	10.92437	-7.88600
C	15.60802	7.57302	-15.89530
O	16.39829	7.99103	-16.75140
N	14.92236	6.42241	-16.03004
H	14.28907	6.11796	-15.29354
C	15.09167	5.60463	-17.22093
H	16.15926	5.61128	-17.47619
C	14.65130	4.14883	-16.94272
H	14.77642	3.94817	-15.87073
H	13.58228	4.02233	-17.16097
C	15.47523	3.15706	-17.69562
N	15.60029	1.83429	-17.31262
H	15.13508	1.39686	-16.52822
C	16.46742	1.21469	-18.13805
H	16.75505	0.17649	-18.04681
N	16.90332	2.07642	-19.04901
C	16.29064	3.29082	-18.78191
H	16.46207	4.16882	-19.38712
C	14.39173	6.20384	-18.45783

O	14.80684	5.93832	-19.59173
N	13.34895	7.01850	-18.23086
H	12.96241	7.10885	-17.29407
C	12.65849	7.69470	-19.32316
H	12.37361	6.96603	-20.09310
C	11.41099	8.40553	-18.78131
H	10.84661	7.68483	-18.18020
H	11.72052	9.21298	-18.10243
C	10.52998	8.96277	-19.89888
H	11.14219	9.48268	-20.64653
H	10.02654	8.13867	-20.42091
C	9.50528	9.97705	-19.40852
H	10.03198	10.84691	-18.98752
H	8.91669	10.32551	-20.26971
N	8.61841	9.41226	-18.38902
H	8.68218	8.41457	-18.11116
C	7.66909	10.12895	-17.78778
N	7.45164	11.40837	-18.14144
H	7.79380	11.75819	-19.02225
H	6.67395	11.90384	-17.73401
N	6.93649	9.55927	-16.81697
H	7.38804	8.78106	-16.28886
H	6.31544	10.16036	-16.29587
C	13.60274	8.68779	-20.02463
O	13.73820	8.69366	-21.24997
N	14.24057	9.55303	-19.20439
H	14.12523	9.44588	-18.20221
C	15.27360	10.47411	-19.68432
H	15.72866	10.90953	-18.78556
C	14.73369	11.59020	-20.58102
H	15.56474	12.27110	-20.80407
H	14.42503	11.14696	-21.53095

C	13.56032	12.38751	-20.00543
H	13.15479	13.01069	-20.81030
H	12.74629	11.71427	-19.70191
C	13.90606	13.33133	-18.85588
H	14.83466	13.86104	-19.10004
H	13.10930	14.07838	-18.73472
N	14.09502	12.60145	-17.59886
H	13.47537	11.82151	-17.41568
C	14.85183	13.01268	-16.57644
N	15.53179	14.16645	-16.64369
H	15.24560	14.88711	-17.28736
H	16.06432	14.46657	-15.84127
N	14.96048	12.23286	-15.49808
H	14.57416	11.29291	-15.52180
H	15.51001	12.52328	-14.70556
C	16.38178	9.66339	-20.39821
O	16.75041	9.91974	-21.54969
N	16.89850	8.68488	-19.63417
H	16.50461	8.56846	-18.70228
C	17.64374	7.50494	-20.06770
H	16.94228	6.65889	-20.03015
C	18.78631	7.22544	-19.06879
H	19.64307	7.86802	-19.30420
H	18.43801	7.48749	-18.06426
C	19.13765	5.77842	-19.08212
N	19.47168	5.05673	-17.95365
H	19.61251	5.43977	-17.02738
C	19.55940	3.75183	-18.27691
H	19.77561	2.96504	-17.56712
N	19.32170	3.59596	-19.57638
C	19.06058	4.85377	-20.08549
H	18.79549	5.00760	-21.12067

C	18.20313	7.60675	-21.49602
O	19.28431	8.15477	-21.72730
N	17.49638	6.95505	-22.44818
H	16.61281	6.52579	-22.20415
C	18.02148	6.78582	-23.79334
H	19.11643	6.80727	-23.71155
C	17.62174	5.43065	-24.40617
H	16.53260	5.31393	-24.39872
H	17.95395	5.43743	-25.44964
C	18.23558	4.30381	-23.65737
N	19.60316	4.11008	-23.56848
H	20.31395	4.70742	-24.00528
C	19.83853	3.09037	-22.72532
H	20.82747	2.75550	-22.44242
N	18.68599	2.58613	-22.27815
C	17.67833	3.34558	-22.85787
H	16.63174	3.15715	-22.65965
C	17.74864	7.93658	-24.76564
O	18.25250	7.89228	-25.88925
N	17.03228	8.98552	-24.31167
H	16.69879	8.98034	-23.35184
C	17.11806	10.26525	-24.99933
H	17.15016	10.06735	-26.07236
C	15.89424	11.14395	-24.69986
H	15.80832	11.30608	-23.61957
H	16.06303	12.11905	-25.17274
C	14.63878	10.52357	-25.21582
N	14.43036	10.29207	-26.56549
C	13.24131	9.71461	-26.65780
H	12.75765	9.39592	-27.57272
N	12.68094	9.56373	-25.43645
H	11.77814	9.15267	-25.24582

C	13.55840	10.06969	-24.50382
H	13.35145	10.04930	-23.44351
C	18.43051	11.01045	-24.69087
O	18.93248	11.75613	-25.53565
N	18.95954	10.80382	-23.46848
Cu	18.26251	2.12850	-20.43064
H	11.11603	9.24508	-15.55984
H	11.93138	11.10631	-13.96320
H	11.78978	9.92843	-12.65448
H	9.91161	12.25047	-14.88083
H	8.31287	11.50619	-14.67465
H	9.53591	10.70421	-15.66493
H	10.25295	12.35203	-12.31583
H	9.97858	10.88562	-11.35335
H	8.61395	11.69518	-12.14997
H	9.32014	9.63845	-13.38677
H	19.79766	11.25249	-23.21258
H	18.50782	10.20423	-22.83155
C	11.48899	7.99966	-9.83790
H	11.58560	9.03536	-10.08868
H	11.62126	7.87413	-8.78355
H	10.51620	7.65386	-10.11899

$[CuH_3L]^{5+}$ with {His-11, D-His-14, His-15} coordination

202

N	12.14345	5.17112	-11.02092
C	12.73546	4.46931	-12.14426
H	13.74341	4.88140	-12.28342
C	12.84838	2.95637	-11.88342
H	12.99884	2.80571	-10.80634

H	11.91519	2.44128	-12.14681
C	14.01067	2.35644	-12.60603
N	14.39751	1.04011	-12.44167
C	15.52201	0.81546	-13.14955
N	15.88355	1.93320	-13.77094
C	14.94563	2.89671	-13.44504
H	15.00584	3.89355	-13.84829
C	12.01628	4.79259	-13.46020
O	12.67632	4.81871	-14.52141
N	10.71623	5.07257	-13.42557
H	10.19185	4.98715	-12.55411
C	10.01132	5.55025	-14.61696
H	10.08978	4.80937	-15.42259
C	8.54121	5.81482	-14.26288
H	8.06238	4.85748	-14.03257
H	8.50952	6.41813	-13.34824
C	7.77795	6.54111	-15.36343
H	8.27626	7.48095	-15.63584
H	7.74602	5.94001	-16.28308
C	6.33080	6.89445	-14.99038
O	5.73944	7.71451	-15.74950
O	5.83037	6.34501	-13.96942
C	10.66636	6.82559	-15.15757
O	10.90289	6.95607	-16.36660
N	10.91742	7.79169	-14.26047
C	11.47738	9.07071	-14.67601
C	11.51489	10.02717	-13.47781
C	10.13086	10.55983	-13.07272
C	9.57900	11.54060	-14.10809
C	10.21895	11.21974	-11.69572
C	12.86143	8.91168	-15.30719
O	13.21424	9.65332	-16.24125

N	13.65891	7.95872	-14.80155
H	13.33227	7.40390	-14.01533
C	14.95977	7.67386	-15.39212
H	15.47007	8.62246	-15.59769
C	15.80812	6.82557	-14.44340
H	15.27861	5.88286	-14.25496
H	16.74643	6.57923	-14.95611
C	16.09854	7.52417	-13.11728
H	16.68230	8.43565	-13.29536
H	15.16107	7.81956	-12.63488
C	16.84272	6.59274	-12.15521
H	16.32440	5.62716	-12.11001
H	17.85820	6.37969	-12.51568
N	16.86386	7.10191	-10.78572
H	16.09957	6.78783	-10.15524
C	17.56311	8.16197	-10.37644
N	18.57489	8.65545	-11.11178
H	18.99785	8.09160	-11.83193
H	19.14479	9.39449	-10.73009
N	17.23868	8.72460	-9.20369
H	16.27207	8.58961	-8.85545
H	17.75078	9.53608	-8.89463
C	14.83339	6.98832	-16.76360
O	15.61486	7.26750	-17.67241
N	13.85867	6.06219	-16.91705
H	13.21039	5.87079	-16.15943
C	13.61758	5.49107	-18.23213
H	14.55031	5.07799	-18.63050
C	12.56052	4.36946	-18.18616
H	11.69557	4.70492	-17.59944
H	12.21516	4.20252	-19.21073
C	13.05716	3.06401	-17.67233

N	13.11001	2.73563	-16.32986
H	12.87707	3.37804	-15.55975
C	13.53023	1.47982	-16.18740
H	13.64666	0.94102	-15.25941
N	13.75396	0.99102	-17.40643
H	14.07933	0.05170	-17.59682
C	13.47408	1.95427	-18.35080
H	13.57462	1.77172	-19.40970
C	13.15835	6.53497	-19.26265
O	13.41045	6.36176	-20.46157
N	12.44814	7.56527	-18.78465
H	12.17127	7.55589	-17.80569
C	11.87529	8.59188	-19.64356
H	11.38604	8.11885	-20.50555
C	10.83950	9.37297	-18.82150
H	10.18325	8.63232	-18.34933
H	11.35129	9.90384	-18.00641
C	10.01655	10.35375	-19.64920
H	10.67343	11.01274	-20.22919
H	9.39325	9.81150	-20.37239
C	9.14852	11.26476	-18.78921
H	9.79442	11.83483	-18.10589
H	8.62266	11.97755	-19.44052
N	8.18136	10.50204	-17.98894
H	7.97694	9.54866	-18.25515
C	7.36683	11.07406	-17.09164
N	7.43844	12.39496	-16.88081
H	7.93000	13.00106	-17.51655
H	6.84847	12.82919	-16.18929
N	6.50206	10.33732	-16.39209
H	6.48469	9.31001	-16.38153
H	5.88709	10.79709	-15.73889

C	12.95953	9.51265	-20.24813
O	12.92991	9.79618	-21.45134
N	13.84186	10.01881	-19.36294
H	13.78875	9.65678	-18.41416
C	15.05110	10.79051	-19.65581
H	15.30579	11.29747	-18.71544
C	14.91768	11.85000	-20.76665
H	15.84178	12.44089	-20.73696
H	14.89059	11.34856	-21.73749
C	13.72257	12.80093	-20.68028
H	13.77846	13.46629	-21.54898
H	12.77990	12.25028	-20.78213
C	13.66118	13.69059	-19.44381
H	14.65726	14.09844	-19.22170
H	12.98266	14.53284	-19.63978
N	13.17998	12.93128	-18.28677
H	12.76384	12.02627	-18.46015
C	13.11171	13.40406	-17.04189
N	13.48613	14.66007	-16.77556
H	13.59542	15.33327	-17.51763
H	13.41651	15.02038	-15.83671
N	12.66629	12.60193	-16.06427
H	12.69746	11.59095	-16.19276
H	12.66867	12.94006	-15.11438
C	16.21257	9.83691	-20.01806
O	16.01643	8.74863	-20.55663
N	17.44598	10.30251	-19.73216
H	17.54197	11.17751	-19.23837
C	18.66240	9.52951	-19.99458
H	19.48814	10.12455	-19.58716
C	18.93932	9.30793	-21.49254
H	19.86644	8.73005	-21.57487

H	18.12598	8.72971	-21.94397
C	19.09964	10.58653	-22.24666
N	18.03921	11.20550	-22.88295
C	18.53981	12.30371	-23.42566
H	17.99340	13.03573	-24.00732
N	19.86784	12.41942	-23.16911
H	20.47438	13.16115	-23.48852
C	20.24366	11.32838	-22.41999
H	21.26156	11.17714	-22.08986
C	18.64474	8.26417	-19.11256
O	18.59283	8.38284	-17.88808
N	18.71330	7.07032	-19.74223
H	18.71010	7.03806	-20.75199
C	18.60265	5.80500	-19.03782
H	18.50676	6.04342	-17.97387
C	17.33918	5.04192	-19.47491
H	16.51204	5.75842	-19.45076
H	17.45151	4.66783	-20.49661
C	17.07265	3.92390	-18.53050
N	17.16908	2.58411	-18.84276
C	16.98136	1.85824	-17.72346
N	16.74740	2.67310	-16.69741
C	16.80304	3.96121	-17.18941
C	19.87135	4.96700	-19.29306
O	19.84593	4.01123	-20.07148
N	21.02463	5.34592	-18.68942
H	21.85140	4.85085	-19.01502
C	21.21271	6.29553	-17.60004
H	20.25885	6.78458	-17.38367
C	21.69383	5.57348	-16.31863
H	22.70798	5.17804	-16.45028
H	21.72187	6.29898	-15.49921

C	20.80559	4.43053	-15.95808
N	21.09225	3.13347	-16.34080
C	20.10991	2.32137	-15.92041
N	19.19319	3.03191	-15.26668
C	19.61168	4.35244	-15.29203
H	19.03825	5.14378	-14.82999
C	22.11322	7.43201	-18.12446
O	21.62766	8.27992	-18.88140
N	23.41385	7.43218	-17.77594
Cu	17.42049	2.39254	-14.83478
H	10.69005	7.64850	-13.27797
H	10.85899	9.48869	-15.48186
H	12.16970	10.87466	-13.71605
H	11.98043	9.49753	-12.63537
H	9.51774	11.09674	-15.10781
H	10.21942	12.42929	-14.17402
H	8.57074	11.86810	-13.83214
H	10.92543	12.05944	-11.71570
H	10.55967	10.50496	-10.93827
H	9.24262	11.60799	-11.38588
H	9.44148	9.70746	-12.99628
H	16.67415	4.82220	-16.55029
H	17.02809	0.77852	-17.68459
H	17.35798	2.20652	-19.76260
H	16.03214	-0.13702	-13.19201
H	13.92453	0.34894	-11.87334
H	20.08151	1.25520	-16.10001
H	21.91461	2.83668	-16.85088
H	24.00962	8.14682	-18.09779
H	23.76903	6.71766	-17.19927
C	11.26650	5.12436	-10.30665
H	12.71082	5.99236	-10.81100

C	10.83329	6.67403	-9.66779
O	10.32166	4.31440	-10.41080
H	11.62114	7.39172	-9.76320
H	10.83166	6.71517	-8.86484
H	9.85328	6.94919	-9.60902

$[CuL]^{2+}$ with {His-11, His-15, His-16, N_{am}-16} coordination

200

C	11.330043	6.440227	-10.545563
O	12.141099	7.192064	-11.109847
N	11.384928	5.100784	-10.719728
H	10.724246	4.502675	-10.243325
C	12.432611	4.505803	-11.532426
H	13.415379	4.797363	-11.138927
C	12.307156	2.967892	-11.495525
H	12.393162	2.652497	-10.447069
H	11.312091	2.675525	-11.850068
C	13.323928	2.284773	-12.339155
N	14.680313	2.365471	-12.109288
H	15.129687	2.861855	-11.352350
C	15.310316	1.658808	-13.086700
H	16.388646	1.568909	-13.133763
N	14.444731	1.122744	-13.928655
C	13.201876	1.513658	-13.468592
H	12.285660	1.222622	-13.967476
C	12.421504	5.015827	-12.974254
O	13.486220	5.165599	-13.586490
N	11.227056	5.254150	-13.543754
H	10.378780	5.154342	-13.001198
C	11.160274	5.751400	-14.912163
H	11.698347	5.056555	-15.567709

C	9.702759	5.831407	-15.377098
H	9.266081	4.836327	-15.224846
H	9.148128	6.541271	-14.752394
C	9.571336	6.221211	-16.849631
H	10.285437	5.664166	-17.466932
H	8.569383	5.933908	-17.197188
C	9.691361	7.719363	-17.153067
O	10.142604	8.049511	-18.288461
O	9.275336	8.526724	-16.272670
C	11.915293	7.077174	-15.066462
O	12.602069	7.287735	-16.070759
N	11.788220	7.984047	-14.075943
H	11.302457	7.737630	-13.222176
C	12.526717	9.236810	-14.126252
C	12.030828	10.210286	-13.051230
C	10.625802	10.771723	-13.310078
C	10.609626	11.706616	-14.521085
C	10.131459	11.506033	-12.061636
C	14.034507	9.026501	-13.978783
O	14.840112	9.742484	-14.597222
N	14.438917	8.057362	-13.140758
H	13.755128	7.470708	-12.672194
C	15.856871	7.761862	-13.010490
H	16.374653	8.658611	-12.650731
C	16.114043	6.623574	-12.020294
H	15.563333	5.729189	-12.338705
H	17.185006	6.388059	-12.069888
C	15.741863	6.999927	-10.586708
H	16.253668	7.930297	-10.305777
H	14.663706	7.182365	-10.501283
C	16.128537	5.879492	-9.619869
H	15.594899	4.958668	-9.879626

H	17.196987	5.643870	-9.692798
N	15.790791	6.185161	-8.230562
H	14.863570	5.941309	-7.914317
C	16.560154	6.873101	-7.382401
N	17.768907	7.315552	-7.748313
H	18.046924	7.338920	-8.715251
H	18.313181	7.866387	-7.103563
N	16.143694	7.073928	-6.125775
H	15.237773	6.749739	-5.827915
H	16.649138	7.691849	-5.511057
C	16.490209	7.429234	-14.369649
O	17.632211	7.828280	-14.643625
N	15.759265	6.652696	-15.195528
C	16.281800	6.165606	-16.462610
H	17.348996	6.429740	-16.476909
C	16.187971	4.623481	-16.567620
H	17.015756	4.267605	-17.187360
H	16.356630	4.224626	-15.561837
C	14.918711	4.066781	-17.115416
N	14.701621	3.934167	-18.479583
C	13.492023	3.413012	-18.634017
N	12.932236	3.190516	-17.428543
C	13.817138	3.592687	-16.455896
H	13.603949	3.510634	-15.400678
C	15.719672	6.912744	-17.677517
O	15.790155	6.428090	-18.818221
N	15.253937	8.150697	-17.455131
H	15.088316	8.445863	-16.498162
C	14.899177	9.074865	-18.522469
H	14.798528	8.493219	-19.445985
C	13.564387	9.799226	-18.217520
H	12.921332	9.094461	-17.679805

H	13.757617	10.642693	-17.538761
C	12.869393	10.294937	-19.487884
H	13.605339	10.757578	-20.151311
H	12.446104	9.437448	-20.028724
C	11.792913	11.340558	-19.234954
H	12.236429	12.187961	-18.692693
H	11.425875	11.716582	-20.202447
N	10.681316	10.799338	-18.450260
H	10.532842	9.769084	-18.407024
C	9.668118	11.555313	-18.028416
N	9.685932	12.892223	-18.203750
H	10.254876	13.283580	-18.939163
H	8.840140	13.404415	-18.001687
N	8.635506	10.972823	-17.399724
H	8.817100	10.045402	-16.962646
H	7.964555	11.577187	-16.949409
C	15.991418	10.129780	-18.778464
O	15.947123	10.820846	-19.799673
N	16.897726	10.297928	-17.793852
H	16.957368	9.582363	-17.079801
C	18.009968	11.241701	-17.868628
H	17.737413	12.008953	-18.600305
C	18.259565	11.873170	-16.499822
H	18.566061	11.089439	-15.796024
H	19.101418	12.571203	-16.581795
C	17.022836	12.573605	-15.933109
H	16.859389	13.536657	-16.431314
H	16.132187	11.961262	-16.119922
C	17.135401	12.822939	-14.427283
H	17.984765	13.477332	-14.215218
H	16.230066	13.315771	-14.056007
N	17.322598	11.548234	-13.714924

H	16.695282	10.796113	-13.994332
C	18.454965	11.188476	-13.093960
N	19.272238	12.122529	-12.580119
H	18.902181	13.022279	-12.317070
H	20.115574	11.829269	-12.111621
N	18.782072	9.899195	-12.995686
H	18.344539	9.183260	-13.585785
H	19.535008	9.621412	-12.385602
C	19.205361	10.471791	-18.471805
O	19.431224	10.522665	-19.679710
N	19.932217	9.717994	-17.601622
H	19.575605	9.664194	-16.657308
C	20.640714	8.513822	-18.024199
H	21.018611	8.050548	-17.102849
C	21.864899	8.786283	-18.926288
H	22.370520	7.833248	-19.114529
H	21.535667	9.188240	-19.886921
C	22.829287	9.733585	-18.285206
N	22.849390	11.083374	-18.588804
C	23.787715	11.610252	-17.818508
H	24.081988	12.652042	-17.791238
N	24.368100	10.666814	-17.033780
H	25.117510	10.816131	-16.373509
C	23.771888	9.461330	-17.323095
H	24.060274	8.543987	-16.829818
C	19.600815	7.524659	-18.611848
O	18.400452	7.797870	-18.615719
N	20.051848	6.330212	-19.038466
H	21.038282	6.116518	-19.044620
C	19.100200	5.308067	-19.463737
H	18.262461	5.342682	-18.759645
C	19.783335	3.942374	-19.403010

H	20.491148	3.845479	-20.236387
H	20.376614	3.898833	-18.479374
C	18.820866	2.795243	-19.391930
N	17.454233	2.947104	-19.240465
C	16.952400	1.721668	-19.128709
N	17.931050	0.798811	-19.215198
C	19.125723	1.457403	-19.384771
C	18.585985	5.611549	-20.884391
O	19.405819	6.031424	-21.731606
N	17.285669	5.423800	-21.128753
C	16.893934	5.809435	-22.483279
H	17.387854	5.169118	-23.231102
C	15.369238	5.701134	-22.687523
H	14.867611	6.317961	-21.928240
H	15.112574	6.105405	-23.671735
C	14.908734	4.294897	-22.595880
N	15.061436	3.591604	-21.418676
C	14.609947	2.362199	-21.642416
N	14.173734	2.257431	-22.913097
C	14.355386	3.470600	-23.538732
H	14.084182	3.637277	-24.570436
C	17.262717	7.274674	-22.782622
O	17.056683	8.179411	-21.970921
N	17.683181	7.505334	-24.044685
Cu	16.029210	4.543892	-19.859144
H	12.401221	9.675944	-15.123343
H	12.743208	11.044446	-12.992999
H	12.059019	9.696139	-12.080712
H	10.798535	12.342959	-11.819124
H	10.097898	10.837568	-11.194127
H	9.126705	11.912184	-12.220361
H	9.601721	12.097062	-14.696981

H	10.929767	11.202357	-15.439297
H	11.280501	12.558314	-14.350444
H	9.946223	9.928585	-13.504582
H	12.016616	2.791543	-17.273977
H	17.805058	-0.202029	-19.156278
H	20.067976	0.934957	-19.461131
H	13.774905	1.426942	-23.328199
H	14.850527	6.319916	-14.882342
H	14.573365	1.544928	-20.936100
H	15.912213	1.471839	-18.972598
H	13.003981	3.192037	-19.572472
H	17.952484	8.441585	-24.309700
H	17.973514	6.743247	-24.636973
C	10.230163	6.974237	-9.670905
H	9.583267	7.613459	-10.280315
H	10.674298	7.600721	-8.891777
H	9.627659	6.190191	-9.207205

$[CuL]^{2+}$ with {His-11, His-15, His-16, N_{am}-15} coordination

200

C	11.797659	7.348595	-10.129727
O	12.612314	8.048090	-10.753433
N	11.890758	6.001574	-10.136433
H	11.225874	5.446593	-9.615797
C	12.988231	5.341681	-10.826492
H	13.942011	5.741815	-10.457263
C	12.915289	3.826814	-10.552865
H	12.932823	3.687918	-9.463144
H	11.958666	3.436937	-10.919540
C	14.009702	3.045691	-11.189187

N	15.345223	3.218335	-10.893389
H	15.733597	3.884900	-10.239950
C	16.052511	2.307470	-11.616785
H	17.131824	2.234743	-11.558565
N	15.257161	1.558439	-12.359429
C	13.980303	2.017946	-12.099809
H	13.105638	1.583971	-12.568616
C	12.995238	5.636871	-12.329937
O	14.059720	5.849777	-12.918943
N	11.808509	5.615733	-12.967253
H	10.956307	5.461847	-12.443753
C	11.738801	5.894432	-14.396701
H	12.443115	5.233493	-14.919512
C	10.324334	5.625051	-14.926237
H	10.091556	4.577838	-14.697684
H	9.602048	6.254091	-14.393124
C	10.190354	5.854299	-16.432790
H	11.030903	5.406092	-16.973763
H	9.287746	5.335787	-16.785005
C	10.011732	7.311578	-16.867248
O	10.362737	7.628932	-18.049276
O	9.468667	8.105029	-16.055105
C	12.227745	7.309018	-14.723861
O	12.851714	7.517400	-15.768659
N	11.943027	8.288274	-13.842916
H	11.510008	8.059361	-12.957644
C	12.455638	9.637415	-14.042646
C	11.840144	10.603338	-13.023383
C	10.371454	10.953466	-13.304352
C	10.252243	11.909710	-14.492550
C	9.736414	11.568001	-12.055332
C	13.985006	9.678538	-13.955873

O	14.657723	10.332171	-14.765023
N	14.547807	8.992155	-12.945891
H	13.966732	8.456429	-12.305933
C	15.995588	8.945422	-12.792098
H	16.387741	9.949860	-12.583586
C	16.344607	7.991520	-11.641611
H	15.967232	8.436964	-10.711342
H	15.802915	7.049204	-11.795278
C	17.837828	7.706702	-11.513414
H	18.215616	7.250708	-12.438009
H	18.404557	8.632183	-11.353750
C	18.082397	6.748848	-10.344931
H	17.820246	7.231094	-9.398180
H	17.436083	5.865092	-10.429145
N	19.476969	6.328259	-10.223778
H	20.075164	6.874038	-9.621045
C	20.059545	5.387214	-10.972422
N	19.355860	4.700158	-11.883155
H	18.350188	4.759522	-11.903995
H	19.767094	3.892998	-12.326564
N	21.368264	5.149630	-10.834182
H	21.916850	5.655912	-10.157824
H	21.819860	4.417700	-11.358590
C	16.687096	8.510398	-14.088602
O	17.703039	9.089805	-14.492039
N	16.163778	7.436224	-14.719813
C	16.802507	6.878169	-15.897022
H	17.780471	7.382183	-15.972443
C	17.087129	5.372138	-15.749767
H	17.850067	5.085661	-16.479276
H	17.535312	5.232232	-14.760699
C	15.924081	4.459515	-15.903223

N	15.450989	4.061040	-17.145884
C	14.470921	3.198919	-16.903313
N	14.299492	3.032260	-15.575024
C	15.214607	3.819755	-14.923005
H	15.279313	3.863852	-13.848049
C	16.107184	7.259204	-17.204088
O	16.380408	6.665372	-18.271438
N	15.343384	8.354201	-17.200151
H	15.134424	8.815778	-16.318832
C	14.764785	8.914435	-18.421573
H	14.327451	8.096709	-19.004200
C	13.658220	9.919691	-18.064787
H	13.009114	9.421986	-17.334477
H	14.097842	10.793480	-17.566454
C	12.843606	10.362714	-19.279351
H	13.483290	10.900971	-19.983880
H	12.458697	9.485304	-19.815508
C	11.683292	11.285867	-18.931259
H	12.047632	12.119201	-18.312728
H	11.279475	11.708368	-19.863024
N	10.625889	10.571154	-18.210897
H	10.632235	9.542508	-18.190963
C	9.489109	11.158688	-17.826414
N	9.295838	12.468409	-18.031087
H	9.985176	13.039042	-18.490848
H	8.484891	12.929194	-17.651156
N	8.530310	10.411174	-17.257599
H	8.834768	9.519086	-16.816191
H	7.756918	10.895277	-16.826384
C	15.833271	9.500129	-19.362734
O	15.562385	9.693273	-20.546515
N	17.040006	9.771210	-18.809631

H	17.156819	9.540004	-17.830553
C	18.285855	9.807459	-19.586843
H	18.077409	10.268620	-20.557198
C	19.332644	10.600409	-18.812600
H	19.463366	10.149413	-17.818627
H	20.298372	10.523252	-19.325231
C	18.944778	12.072692	-18.629876
H	19.222408	12.652732	-19.515872
H	17.856593	12.167757	-18.518107
C	19.616822	12.703452	-17.415050
H	20.702624	12.547087	-17.462624
H	19.424732	13.785339	-17.400062
N	19.103160	12.079389	-16.188621
H	18.202676	11.621025	-16.235976
C	19.659831	12.202745	-14.980617
N	20.749709	12.966309	-14.820872
H	20.975280	13.679030	-15.496670
H	21.147123	13.075649	-13.900840
N	19.135033	11.548107	-13.937317
H	18.530209	10.737618	-14.086690
H	19.582797	11.627149	-13.037020
C	18.591565	8.311260	-19.836389
O	18.047182	7.724932	-20.778865
N	19.356227	7.710433	-18.905755
H	19.760335	8.280038	-18.167214
C	19.332402	6.280066	-18.596419
H	18.466240	6.087559	-17.964339
C	20.602241	5.904727	-17.807370
H	20.567567	4.827526	-17.609270
H	21.478606	6.099221	-18.436961
C	20.711016	6.632829	-16.505862
N	20.916389	8.004021	-16.446874

C	20.936027	8.310986	-15.160306
H	21.068189	9.300226	-14.740300
N	20.763452	7.208302	-14.391659
H	20.743087	7.185264	-13.382717
C	20.612847	6.128967	-15.232636
H	20.459734	5.125523	-14.862822
C	19.216356	5.392638	-19.828768
O	20.158990	5.403298	-20.651187
N	18.142936	4.574322	-19.866067
C	18.194014	3.604006	-20.961146
H	19.208557	3.587558	-21.381781
C	17.876648	2.182389	-20.452218
H	18.252753	1.435367	-21.156543
H	18.413144	2.045493	-19.505576
C	16.409627	1.994100	-20.298662
N	15.594979	3.051640	-19.943986
C	14.335319	2.645504	-20.092044
N	14.318806	1.365701	-20.501815
C	15.617484	0.934143	-20.646046
C	17.258302	3.971493	-22.118131
O	16.972061	3.165690	-23.009837
N	16.749446	5.222422	-22.072345
H	17.100706	5.865309	-21.366845
C	15.632254	5.631733	-22.896620
H	14.971229	4.767911	-23.047835
C	14.868476	6.766306	-22.193256
H	15.600405	7.519321	-21.868397
H	14.225575	7.250537	-22.935041
C	13.994741	6.397213	-21.033088
N	14.378297	5.704581	-19.893075
C	13.346147	5.770534	-19.056238
N	12.318102	6.450996	-19.596298

C	12.711464	6.857000	-20.847177
H	12.075961	7.446096	-21.492698
C	16.026989	6.097957	-24.307565
O	15.163880	6.193746	-25.183592
N	17.318520	6.418776	-24.493219
Cu	16.180132	4.775094	-19.098998
H	12.221753	9.956598	-15.065435
H	12.433541	11.527785	-13.018742
H	11.940617	10.156985	-12.025037
H	10.271815	12.480992	-11.765462
H	9.770599	10.871986	-11.209597
H	8.689720	11.834122	-12.238590
H	9.202582	12.099232	-14.740743
H	10.740414	11.511593	-15.388525
H	10.725680	12.869964	-14.251581
H	9.833735	10.023750	-13.544214
H	13.615381	2.425731	-15.144634
H	13.485801	0.826365	-20.693327
H	15.864386	-0.050144	-21.014252
H	11.474621	6.758246	-19.067320
H	15.343598	6.973849	-14.334234
H	13.875019	2.673451	-17.635854
H	13.443786	3.230998	-19.920769
H	13.320901	5.355475	-18.057184
H	17.630531	6.735560	-25.398877
H	17.996714	6.302228	-23.756118
C	10.656779	7.955447	-9.362043
H	10.002082	8.472977	-10.071341
H	11.055933	8.706866	-8.674965
H	10.073471	7.219771	-8.804599

$[CuL]^{2+}$ with {D-His-14, His-15, His-16, N_{am}-16} coordination

200

C	12.02439	6.43167	-10.00619
O	12.73396	7.04201	-10.80422
N	11.85427	5.10375	-10.04625
H	11.39495	4.63998	-9.27311
C	12.67898	4.28385	-10.92232
H	13.73622	4.37209	-10.64150
C	12.19464	2.82673	-10.79113
H	12.46273	2.46493	-9.79691
H	11.10305	2.81861	-10.87242
C	12.77052	1.93307	-11.83836
N	13.91520	1.20230	-11.64109
H	14.46314	1.18579	-10.80451
C	14.15607	0.50160	-12.77674
H	15.00239	-0.15057	-12.89635
N	13.23996	0.75136	-13.67812
C	12.36878	1.64108	-13.11240
H	11.50956	2.01282	-13.64836
C	12.51923	4.77616	-12.36136
O	13.48571	4.94553	-13.10755
N	11.25662	4.95540	-12.73070
H	10.51646	4.79774	-12.05266
C	10.85523	5.39627	-14.06186
H	11.09495	4.62003	-14.80254
C	9.35029	5.68630	-14.00185
H	8.82595	4.78272	-13.67764
H	9.20178	6.44678	-13.23155
C	8.74463	6.20133	-15.30213
H	9.42616	6.88531	-15.81242
H	8.51821	5.38100	-15.98914

C	7.41020	6.96633	-14.98572
O	7.11399	7.87262	-15.80568
O	6.78949	6.62541	-13.97264
C	11.61224	6.66448	-14.44527
O	12.07805	6.82050	-15.57377
N	11.66897	7.60241	-13.49721
H	11.33086	7.42727	-12.55640
C	12.32920	8.86930	-13.74579
C	12.06803	9.82610	-12.57954
C	10.62191	10.35133	-12.57805
C	10.45131	11.47948	-13.59844
C	10.24532	10.84106	-11.17843
C	13.82784	8.65477	-13.93025
O	14.44436	9.20077	-14.84836
N	14.41871	7.84787	-13.05026
H	13.89427	7.39848	-12.30662
C	15.85348	7.62554	-13.13301
H	16.37897	8.58163	-13.01311
C	16.30272	6.62566	-12.06562
H	15.72711	5.70109	-12.17663
H	17.35675	6.39186	-12.24047
C	16.12433	7.17588	-10.64893
H	16.63731	8.13682	-10.54769
H	15.06191	7.33508	-10.44074
C	16.68405	6.18662	-9.61791
H	16.29258	5.18331	-9.81284
H	17.77427	6.14665	-9.66641
N	16.24495	6.56998	-8.28799
H	15.21058	6.41107	-8.12198
C	16.86249	7.41956	-7.49271
N	18.13576	7.80994	-7.69811
H	18.71733	7.26212	-8.30269

H	18.60067	8.26505	-6.93950
N	16.22206	7.87873	-6.42522
H	15.19250	7.78178	-6.38273
H	16.65772	8.57182	-5.85446
C	16.19541	7.07676	-14.51624
O	17.18266	7.45735	-15.14394
N	15.35369	6.14664	-14.96637
C	15.58634	5.46148	-16.22484
H	16.66469	5.26937	-16.31937
C	14.84502	4.11244	-16.21400
H	13.84523	4.25415	-15.79165
H	14.74398	3.75207	-17.24124
C	15.63431	3.12356	-15.41110
N	16.45842	2.20587	-16.02024
C	17.06320	1.54693	-15.05902
N	16.66608	2.00193	-13.85225
C	15.76630	3.01178	-14.05572
H	15.30948	3.53474	-13.23506
C	15.22474	6.35079	-17.41896
O	15.76904	6.18366	-18.50530
N	14.33114	7.31393	-17.18536
H	13.93047	7.43176	-16.26458
C	14.02989	8.33468	-18.17202
H	13.83844	7.85481	-19.14335
C	12.79230	9.11632	-17.71819
H	11.96053	8.41474	-17.60228
H	12.98866	9.56053	-16.73908
C	12.42416	10.21769	-18.71398
H	13.16390	11.02070	-18.66625
H	12.42503	9.82274	-19.73465
C	11.05094	10.82423	-18.41219
H	10.97727	11.10716	-17.36131

H	10.90414	11.72154	-19.01811
N	9.98204	9.88914	-18.74603
H	9.75501	9.81324	-19.72390
C	9.17300	9.27988	-17.88158
N	9.33612	9.35107	-16.56759
H	9.93573	10.04617	-16.16777
H	8.55564	9.00225	-16.00362
N	8.21358	8.48862	-18.35456
H	7.96002	8.54436	-19.31817
H	7.52605	8.15407	-17.67998
C	15.22446	9.27539	-18.36855
O	15.36169	9.91802	-19.39856
N	16.09248	9.31920	-17.34522
H	15.83777	8.92696	-16.44913
C	17.27550	10.15704	-17.38636
H	17.10483	10.98538	-18.08687
C	17.58011	10.68244	-15.97951
H	17.71173	9.82754	-15.30906
H	18.52213	11.23681	-16.00958
C	16.46052	11.56575	-15.42012
H	15.49335	11.07933	-15.56902
H	16.60818	11.67753	-14.34362
C	16.39008	12.95150	-16.05072
H	16.30289	12.89442	-17.14118
H	15.51893	13.48776	-15.66257
N	17.60215	13.70865	-15.70918
H	18.14438	13.35735	-14.93508
C	17.77129	14.98407	-16.02277
N	16.97114	15.60115	-16.89348
H	16.05446	15.22436	-17.05282
H	17.05163	16.59519	-16.96511
N	18.84690	15.62640	-15.56397

H	19.34961	15.22819	-14.79650
H	18.87570	16.62123	-15.65077
C	18.48989	9.35986	-17.87563
O	19.48145	9.90877	-18.33369
N	18.36353	8.03373	-17.75095
H	17.59501	7.67714	-17.19797
C	19.47603	7.12022	-17.92450
H	19.33452	6.55488	-18.85872
C	19.48836	6.14913	-16.73372
H	19.40479	6.72444	-15.80830
H	18.60505	5.50458	-16.79696
C	20.72609	5.31199	-16.65863
N	21.47474	4.94754	-17.75283
C	22.48776	4.23305	-17.29961
N	22.42097	4.12417	-15.96377
H	23.06425	3.63314	-15.37760
C	21.31193	4.80068	-15.53790
C	20.83188	7.83008	-17.95247
O	21.39103	8.14961	-16.92061
N	21.54721	7.77723	-19.13031
H	22.48347	8.12384	-18.94187
C	21.11219	8.30496	-20.43502
H	20.89490	9.37678	-20.31669
C	22.28979	8.14578	-21.42310
H	21.87690	8.06091	-22.43632
H	22.89377	9.05690	-21.40408
C	23.23065	6.99761	-21.18417
N	22.88509	5.72837	-20.77120
C	23.99850	5.02283	-20.72760
N	25.04366	5.77550	-21.10317
C	24.58050	7.02921	-21.38956
C	19.86176	7.65384	-21.04721

O	18.93795	8.37817	-21.43672
N	19.96183	6.34383	-21.22960
C	18.84262	5.72312	-21.93514
H	19.20119	4.76723	-22.34507
C	17.66329	5.45272	-21.00073
H	17.38412	6.37925	-20.48208
H	16.79678	5.14687	-21.59718
C	17.96529	4.38285	-19.99820
N	19.23694	3.98203	-19.64014
C	19.09196	3.05795	-18.70323
N	17.79300	2.84251	-18.45957
C	17.06331	3.67072	-19.26393
H	15.98836	3.69570	-19.23868
C	18.41288	6.56293	-23.13990
O	17.24583	6.72983	-23.45810
N	19.45411	6.96264	-23.89673
Cu	21.01286	5.21971	-19.86179
H	11.95188	9.28842	-14.68794
H	12.76859	10.66328	-12.64688
H	12.27423	9.29469	-11.64510
H	10.91603	11.63825	-10.85533
H	10.30966	10.01911	-10.46400
H	9.22364	11.22248	-11.17529
H	9.41078	11.80242	-13.63580
H	10.74359	11.15030	-14.59691
H	11.07127	12.33601	-13.33070
H	9.94852	9.52569	-12.84884
H	16.99906	1.68987	-12.96093
H	25.99727	5.47952	-21.14211
H	25.23338	7.81972	-21.70978
H	17.40139	2.28256	-17.70237
H	14.56997	5.85731	-14.39021

H	21.03332	4.85255	-14.50259
H	23.26552	3.79467	-17.89917
H	24.07679	3.99167	-20.43466
H	19.89040	2.54123	-18.20135
H	17.77828	0.75538	-15.19024
H	19.29740	7.50990	-24.69997
H	20.37136	6.70735	-23.64630
C	11.24404	7.12193	-9.03094
H	11.45411	8.16984	-9.08263
H	11.48828	6.75691	-8.05524
H	10.20461	6.95661	-9.22373

$[ZnH_4L]^+$ with {His-7, D-His-14, His-16} coordination

209

C	11.131454	6.098941	-10.143519
O	11.340556	7.129447	-10.798335
N	11.830043	4.958666	-10.381785
H	11.632523	4.130391	-9.837875
C	12.923997	4.939185	-11.336895
H	13.462327	5.894021	-11.241042
C	13.906832	3.771645	-11.051024
H	13.851082	3.546982	-9.978840
H	13.593735	2.871560	-11.588524
C	15.325156	4.068819	-11.397173
N	16.091192	4.976738	-10.691472
H	15.780546	5.533586	-9.905492
C	17.332277	5.000175	-11.213813
H	18.131233	5.629588	-10.845184
N	17.413893	4.144899	-12.225629
C	16.166374	3.556282	-12.346578

H	15.957497	2.808974	-13.098122
C	12.463333	4.935119	-12.792607
O	13.320586	5.053403	-13.691510
N	11.154843	4.890058	-13.059366
H	10.498966	4.784776	-12.295997
C	10.638124	5.210430	-14.392046
H	10.996005	4.471867	-15.119562
C	9.101796	5.183346	-14.361455
H	8.812626	4.251537	-13.859057
H	8.731978	6.017658	-13.753292
C	8.462187	5.202421	-15.747149
H	8.919085	4.440634	-16.389573
H	7.407075	4.914566	-15.642444
C	8.448194	6.535215	-16.507630
O	8.301700	6.447475	-17.762400
O	8.530926	7.612703	-15.855648
C	11.178032	6.568233	-14.863613
O	11.519225	6.741231	-16.040640
N	11.258800	7.535083	-13.928022
H	11.033160	7.326009	-12.958869
C	11.702051	8.880195	-14.253848
C	11.510940	9.812399	-13.050383
C	10.059089	9.979707	-12.579364
C	9.163528	10.557550	-13.675936
C	10.044320	10.871373	-11.335326
C	13.162772	8.937071	-14.700708
O	13.577383	9.935444	-15.317901
N	13.936460	7.892367	-14.371256
H	13.532482	7.133595	-13.831956
C	15.329643	7.764039	-14.743667
H	15.620592	8.714214	-15.214453
C	16.219638	7.563545	-13.513500

H	15.852094	6.706574	-12.933337
H	17.232426	7.326337	-13.858918
C	16.230421	8.838671	-12.674431
H	16.516200	9.675705	-13.324886
H	15.222780	9.057003	-12.297972
C	17.190654	8.770968	-11.488915
H	16.857692	8.023279	-10.763410
H	18.196226	8.467419	-11.808818
N	17.263138	10.042844	-10.764097
H	16.639904	10.159312	-9.977901
C	17.872526	11.142377	-11.218780
N	18.644850	11.099275	-12.306595
H	18.835669	10.233667	-12.782604
H	19.141311	11.919668	-12.616510
N	17.684560	12.313663	-10.590171
H	17.220930	12.328003	-9.694772
H	18.301965	13.086809	-10.787944
C	15.575773	6.736151	-15.853702
O	16.711587	6.623515	-16.318992
N	14.523398	6.056488	-16.364394
H	13.589849	6.230614	-15.998826
C	14.622648	5.493979	-17.703814
H	15.606065	5.024805	-17.819071
C	13.526906	4.439504	-17.932478
H	12.547690	4.868219	-17.681340
H	13.516157	4.175370	-18.993450
C	13.763163	3.194006	-17.154117
N	13.680851	3.112036	-15.772842
H	13.467936	3.878460	-15.119244
C	13.940698	1.865100	-15.378953
H	13.943136	1.505914	-14.359732
N	14.195855	1.143468	-16.469381

H	14.418957	0.156090	-16.465857
C	14.094400	1.942697	-17.587488
H	14.253205	1.563921	-18.585450
C	14.562723	6.583938	-18.796949
O	15.006042	6.356550	-19.926660
N	14.002374	7.746789	-18.428532
H	13.594894	7.810707	-17.506193
C	13.897035	8.933698	-19.269950
H	13.916003	8.618337	-20.317994
C	12.569645	9.621615	-18.941118
H	11.790738	8.859440	-19.067020
H	12.563266	9.913204	-17.880104
C	12.252428	10.829826	-19.812998
H	13.013157	11.611544	-19.690631
H	12.258108	10.539750	-20.871879
C	10.892020	11.446413	-19.467924
H	10.867816	11.776906	-18.424201
H	10.727092	12.339579	-20.078956
N	9.775884	10.534237	-19.729941
H	9.572808	10.372268	-20.706958
C	9.238866	9.653286	-18.867266
N	9.488652	9.699209	-17.557239
H	10.151332	10.356567	-17.181728
H	9.105895	8.965627	-16.933610
N	8.369079	8.731408	-19.312216
H	8.307337	8.588839	-20.309475
H	8.236731	7.889915	-18.708757
C	15.140044	9.835482	-19.082910
O	15.891703	10.078596	-20.027721
N	15.346199	10.298728	-17.828811
H	14.685098	10.064480	-17.093455
C	16.450921	11.184717	-17.460167

H	16.332648	11.352690	-16.379756
C	16.406395	12.542106	-18.171172
H	17.277190	13.118911	-17.843230
H	16.518064	12.375232	-19.246766
C	15.131015	13.353200	-17.919450
H	15.100607	14.171663	-18.647220
H	14.236699	12.744593	-18.109567
C	15.021348	13.997034	-16.537557
H	15.980045	14.461511	-16.280400
H	14.255708	14.784267	-16.555980
N	14.686098	13.010307	-15.504910
H	14.033931	12.274888	-15.750630
C	15.078455	13.058321	-14.229676
N	15.761555	14.109349	-13.755834
H	15.687869	15.006955	-14.208522
H	16.032548	14.115649	-12.784203
N	14.836244	12.008884	-13.437235
H	14.425384	11.172445	-13.832807
H	14.952071	12.074865	-12.438250
C	17.838094	10.517878	-17.540507
O	18.870546	11.186148	-17.617943
N	17.849457	9.181022	-17.377564
H	16.975380	8.679553	-17.308269
C	19.048537	8.422474	-17.092931
H	18.677839	7.399434	-16.969905
C	19.743946	8.842112	-15.771763
H	20.700416	9.345490	-15.957628
H	19.108199	9.590533	-15.282961
C	19.923222	7.687623	-14.837161
N	20.544426	7.802924	-13.609569
H	20.993448	8.632929	-13.244900
C	20.455195	6.620618	-12.962122

H	20.859675	6.446536	-11.973770
N	19.816072	5.736998	-13.716637
C	19.486400	6.391070	-14.888594
H	18.929799	5.891202	-15.664349
C	19.954241	8.282244	-18.316769
O	19.537121	8.443636	-19.464967
N	21.195371	7.797071	-18.072473
H	21.478473	7.583009	-17.124755
C	21.994461	7.275713	-19.170665
H	22.145365	8.061963	-19.919793
C	23.361758	6.820199	-18.616117
H	23.846734	7.685445	-18.148000
H	23.210069	6.059438	-17.842017
C	24.245937	6.243277	-19.658947
N	24.833875	6.995022	-20.661771
H	24.744494	7.997293	-20.780483
C	25.547176	6.206205	-21.465671
H	26.118364	6.519594	-22.327510
N	25.433689	4.963265	-21.002292
H	25.880014	4.155029	-21.417221
C	24.631995	4.954477	-19.880518
H	24.400960	4.049157	-19.340788
C	21.304971	6.122081	-19.922805
O	21.516620	5.957519	-21.122964
N	20.516352	5.313501	-19.177173
H	20.408293	5.504381	-18.188011
C	19.787663	4.185738	-19.727310
H	20.222070	3.969767	-20.711986
C	19.955556	2.930934	-18.843164
H	19.525418	2.079362	-19.378571
H	21.025523	2.743952	-18.716270
C	19.327775	3.057228	-17.497803

N	17.960488	3.023177	-17.297390
H	17.270376	2.923422	-18.034352
C	17.709571	3.180936	-15.986366
H	16.715354	3.216762	-15.562172
N	18.849842	3.306399	-15.316292
C	19.867472	3.235116	-16.251846
H	20.908844	3.311175	-15.971043
C	18.293272	4.479144	-19.970236
O	17.511082	3.531698	-20.133269
N	17.912198	5.759575	-20.009506
H	18.557911	6.523157	-19.856651
Zn	19.046923	3.831055	-13.375542
H	11.130822	9.250435	-15.114494
H	11.906652	10.796804	-13.331405
H	12.126541	9.437247	-12.221041
H	9.566216	11.511471	-14.040265
H	8.156147	10.742123	-13.287275
H	9.066413	9.876825	-14.527283
H	9.662269	8.995451	-12.290755
H	10.423314	11.872450	-11.576934
H	10.671625	10.453248	-10.539943
H	9.026293	10.979660	-10.946584
H	16.922115	5.969462	-20.117364
C	10.089741	6.037476	-9.060640
H	10.356383	6.762149	-8.284847
H	9.991195	5.047323	-8.610877
H	9.128957	6.345299	-9.483663
O	20.807519	3.105499	-12.531697
H	20.639485	2.248664	-12.112626
O	18.654653	1.457820	-12.917488
H	18.428508	0.952653	-13.709095
H	21.233155	3.654746	-11.858757

H 17.972113 1.226948 -12.274377

$[ZnH_4L]^+$ with {His-7, His-11, His-15} coordination

209

C	12.03218	6.03902	-10.52052
O	12.43938	6.82347	-11.36504
N	12.34888	4.71467	-10.58909
H	11.99526	4.08075	-9.85795
C	13.14171	4.15326	-11.67200
H	14.16030	4.61386	-11.65227
C	13.30169	2.62172	-11.52851
H	13.82072	2.44311	-10.55671
H	12.32975	2.08484	-11.51024
C	14.17821	2.17276	-12.68258
N	15.55783	2.07727	-12.54713
H	16.08719	2.18813	-11.66849
C	16.10406	1.88089	-13.85520
H	17.20189	1.78247	-14.02070
N	15.00575	1.92449	-14.79193
C	13.81063	2.02167	-13.97504
H	12.80759	2.07121	-14.34823
C	12.50869	4.42107	-13.00937
O	13.22856	4.64223	-13.96557
N	11.17032	4.29749	-13.16865
H	10.60290	4.09088	-12.33425
C	10.50721	4.43719	-14.45749
H	10.86229	3.62033	-15.12830
C	8.99525	4.32105	-14.22328
H	8.80329	3.34044	-13.73756
H	8.68771	5.13294	-13.52271
C	8.16028	4.42413	-15.50325

H	8.29396	5.42771	-15.95991
H	8.49779	3.64769	-16.22143
C	6.69420	4.24080	-15.23488
O	5.90165	4.31185	-16.15893
O	6.24125	3.99958	-13.98771
C	10.80790	5.76162	-15.11626
O	10.69862	5.85424	-16.32893
N	11.08688	6.85994	-14.36796
H	11.07806	6.77234	-13.34857
C	11.30679	8.16216	-14.96937
C	11.25932	9.27743	-13.91180
C	9.96660	9.27737	-13.06068
C	8.70207	9.32807	-13.92990
C	9.99837	10.46408	-12.09699
C	12.60998	8.22998	-15.72772
O	12.77172	9.14218	-16.50020
N	13.59078	7.30970	-15.55842
H	13.46025	6.61049	-14.84369
C	14.80841	7.27816	-16.36046
H	15.30341	8.26191	-16.29129
C	15.76697	6.19756	-15.81893
H	15.23987	5.21614	-15.86029
H	16.67530	6.13779	-16.45156
C	16.21832	6.46331	-14.37208
H	16.78672	7.41395	-14.31662
H	15.33701	6.54440	-13.70086
C	17.05760	5.29043	-13.87024
H	16.42434	4.39367	-13.97820
H	17.97229	5.10984	-14.47392
N	17.37456	5.43439	-12.46530
H	16.83978	4.86263	-11.79405
C	18.48489	6.18533	-11.96820

N	19.25488	6.86134	-12.75648
H	19.09668	6.90060	-13.77160
H	20.06112	7.37810	-12.37565
N	18.75857	6.15505	-10.57527
H	18.16564	5.59578	-9.94474
H	19.56011	6.66886	-10.18268
C	14.47335	6.96807	-17.81533
O	15.24818	7.27361	-18.70222
N	13.33133	6.31322	-18.12805
H	12.70909	6.03499	-17.37515
C	12.97587	5.94984	-19.48525
H	13.88256	5.55416	-20.00129
C	11.95053	4.82340	-19.43334
H	11.03236	5.15987	-18.91078
H	11.67136	4.59876	-20.48924
C	12.50996	3.54932	-18.78913
N	12.60901	2.38193	-19.53592
H	12.56216	2.35858	-20.57095
C	13.02093	1.29464	-18.68207
H	13.73522	0.66749	-19.23386
N	13.46563	1.97158	-17.43320
C	12.98015	3.31553	-17.54408
H	13.03846	4.03296	-16.74433
C	12.46580	7.15447	-20.27643
O	12.25535	7.04491	-21.47343
N	12.30670	8.35565	-19.66795
H	12.41685	8.40545	-18.65752
C	12.16020	9.60796	-20.38361
H	11.86038	9.44090	-21.44240
C	11.08674	10.47395	-19.69291
H	10.14197	9.90366	-19.54734
H	11.45725	10.74850	-18.68090

C	10.77048	11.73744	-20.51810
H	11.69903	12.19373	-20.90969
H	10.14781	11.44622	-21.39149
C	10.08700	12.81647	-19.67566
H	10.82528	13.15368	-18.93342
H	9.85020	13.69582	-20.31161
N	8.86791	12.30327	-19.08294
H	8.12666	11.99590	-19.73093
C	8.66314	12.11094	-17.67404
N	9.61526	12.54607	-16.70088
H	10.44288	13.10254	-16.93619
H	9.46478	12.33235	-15.70470
N	7.59266	11.51351	-17.27619
H	6.89182	11.18273	-17.95521
H	7.42027	11.35449	-16.27351
C	13.51613	10.32068	-20.31490
O	14.02297	10.75734	-21.33483
N	14.12907	10.47062	-19.09934
H	13.63921	10.10153	-18.29440
C	15.33564	11.21735	-18.76948
H	15.21191	11.49504	-17.70580
C	15.53314	12.53546	-19.53081
H	16.44734	13.03064	-19.13964
H	15.72844	12.30311	-20.59862
C	14.37657	13.53669	-19.45250
H	14.66454	14.38244	-20.11057
H	13.44870	13.09825	-19.86308
C	14.10743	14.11603	-18.05420
H	15.05866	14.50951	-17.63736
H	13.39294	14.95282	-18.18070
N	13.48774	13.13929	-17.18191
H	12.82699	12.46645	-17.59993

C	13.82526	12.99716	-15.79951
N	14.52736	14.01582	-15.10243
H	14.70180	14.93165	-15.53386
H	14.77132	13.88564	-14.10998
N	13.45757	11.94162	-15.16901
H	12.93514	11.22017	-15.66315
H	13.67579	11.80571	-14.17178
C	16.61306	10.40667	-18.76910
O	17.26282	10.34296	-19.79212
N	17.11683	9.93128	-17.58756
H	16.53294	10.02859	-16.74411
C	18.50747	9.52888	-17.35032
H	18.54233	9.07504	-16.33514
C	19.37918	10.80572	-17.32621
H	20.43976	10.56168	-17.10538
H	19.33190	11.29376	-18.32502
C	18.90565	11.74876	-16.25434
N	18.98822	11.46175	-14.87787
H	19.39298	10.60928	-14.46585
C	18.44861	12.57759	-14.14961
H	18.39089	12.70326	-13.04940
N	18.05191	13.50535	-15.17835
H	17.61928	14.42661	-15.02156
C	18.35930	12.94353	-16.43299
H	18.16421	13.42423	-17.38287
C	19.09290	8.47424	-18.28640
O	20.26979	8.55703	-18.60525
N	18.36555	7.40321	-18.69886
H	17.41797	7.31064	-18.35284
C	18.91584	6.37955	-19.58954
H	19.83636	5.98567	-19.10799
C	17.91384	5.21220	-19.81333

H	16.98187	5.58766	-20.29820
H	18.39524	4.51633	-20.53385
C	17.60648	4.40062	-18.55532
N	18.59767	3.91308	-17.69163
H	19.61070	4.05375	-17.81417
C	17.96626	3.22099	-16.58622
H	18.52559	2.41348	-16.05849
N	16.57246	3.12860	-16.98312
C	16.41558	3.95571	-18.15033
H	15.49233	4.11388	-18.69225
C	19.32847	6.93122	-20.96205
O	20.04817	6.25093	-21.67980
N	18.90176	8.15131	-21.38670
H	18.29645	8.68362	-20.77627
C	19.19647	8.72565	-22.69477
H	19.67776	7.97217	-23.35401
C	17.87228	9.18113	-23.34927
H	17.45168	10.04951	-22.79069
H	18.05555	9.50185	-24.39767
C	16.87730	8.05766	-23.33924
N	17.09472	6.80674	-23.94711
H	17.93835	6.52724	-24.46300
C	15.95588	5.97308	-23.68191
H	15.79011	4.91274	-23.95240
N	15.09126	6.80939	-22.90319
H	14.19558	6.51202	-22.51789
C	15.70333	8.06220	-22.73035
H	15.28132	8.87761	-22.16620
C	20.15515	9.90216	-22.58601
O	20.43484	10.53726	-23.59125
N	20.72970	10.24585	-21.40788
H	20.54322	9.71927	-20.55441

Zn	15.34976	1.49590	-16.75958
H	10.49223	8.33820	-15.70559
H	11.33911	10.25357	-14.43150
H	12.13465	9.16881	-13.23151
H	8.72804	10.21173	-14.59840
H	7.79678	9.37363	-13.29126
H	8.60875	8.40922	-14.54685
H	10.01944	11.42162	-12.65999
H	10.90013	10.40560	-11.45003
H	9.10089	10.44605	-11.44395
H	9.91513	8.35748	-12.44164
C	11.16228	6.54739	-9.41332
H	10.22591	6.97151	-9.83570
H	11.70632	7.33429	-8.85408
H	10.89113	5.72974	-8.71311
H	21.39086	11.03267	-21.35438
O	16.94209	0.68097	-16.19812
H	17.38483	0.18392	-16.92199
O	15.77314	1.12102	-18.42292
H	15.61304	1.76890	-19.14245
H	15.95602	0.18856	-18.72823
H	16.81376	0.02309	-15.45879

$[ZnH_4L]^+$ with {His-7, D-His-14, His-15} coordination

209

C	10.952182	5.413754	-9.211953
O	11.386155	6.561480	-9.084159
N	11.443590	4.551024	-10.143736
H	11.065680	3.614573	-10.189620
C	12.547420	4.892157	-11.018918
H	13.004797	5.803936	-10.611917

C	13.602903	3.744899	-11.049858
H	13.555511	3.250145	-10.072428
H	13.314916	3.002932	-11.799960
C	15.022531	4.138699	-11.292762
N	15.797167	4.734457	-10.315004
H	15.478067	5.021050	-9.398620
C	17.057980	4.860078	-10.765782
H	17.865805	5.293577	-10.191266
N	17.147983	4.370636	-11.999477
C	15.882153	3.924828	-12.338526
H	15.677575	3.493218	-13.306103
C	12.110307	5.228987	-12.445324
O	12.971462	5.388980	-13.316816
N	10.797231	5.355766	-12.719699
H	10.112838	5.171263	-11.998112
C	10.349716	5.472041	-14.108469
H	10.752210	4.626035	-14.681585
C	8.817804	5.428005	-14.173126
H	8.509649	4.473787	-13.729053
H	8.395069	6.235305	-13.563049
C	8.285055	5.541827	-15.604304
H	8.927202	5.003607	-16.310326
H	7.300653	5.057124	-15.648951
C	8.045936	6.979212	-16.105226
O	8.219242	7.185414	-17.352149
O	7.633102	7.828596	-15.282259
C	10.930786	6.699914	-14.817444
O	11.431338	6.579661	-15.948393
N	10.869981	7.886647	-14.192886
C	11.459159	9.081091	-14.794030
C	11.101612	10.325871	-13.975749
C	9.632287	10.760130	-14.079443

C	9.273707	11.210399	-15.497085
C	9.367007	11.884118	-13.075406
C	12.984476	8.962465	-14.926815
O	13.592278	9.563127	-15.825517
N	13.585445	8.203416	-13.996358
H	12.998593	7.713050	-13.333813
C	14.997699	7.881178	-13.971833
H	15.548364	8.733894	-14.397015
C	15.463323	7.649950	-12.533727
H	14.813371	6.895665	-12.070840
H	16.471218	7.225881	-12.574552
C	15.471430	8.933743	-11.709667
H	16.151096	9.659473	-12.175664
H	14.473391	9.388824	-11.691221
C	15.913382	8.651630	-10.272938
H	15.186539	8.000669	-9.776396
H	16.869586	8.111792	-10.254855
N	16.016267	9.861432	-9.455426
H	15.197704	10.137798	-8.931819
C	17.026232	10.734842	-9.510117
N	18.106008	10.493106	-10.261027
H	18.240837	9.600431	-10.705560
H	18.910096	11.096610	-10.192064
N	16.945788	11.885641	-8.829352
H	16.177329	12.049169	-8.198779
H	17.755520	12.479426	-8.738947
C	15.393318	6.711585	-14.887080
O	16.587699	6.405143	-14.952475
N	14.427739	6.138456	-15.626135
H	13.461807	6.337023	-15.392311
C	14.679761	5.305566	-16.784627
H	15.721271	4.978757	-16.761020

C	13.740306	4.080826	-16.850524
H	12.708155	4.397720	-16.666199
H	13.781155	3.681181	-17.872088
C	14.073706	2.988352	-15.900530
N	15.240399	2.244155	-15.982929
H	15.994205	2.390990	-16.644656
C	15.243735	1.288084	-15.053255
H	16.026980	0.563243	-14.883535
N	14.103752	1.396525	-14.373465
H	13.836133	0.795643	-13.603513
C	13.358442	2.442773	-14.874842
H	12.393778	2.706707	-14.471707
C	14.519959	6.102704	-18.092371
O	15.343369	5.987053	-19.008850
N	13.432550	6.882622	-18.169550
H	12.776325	6.911302	-17.393433
C	13.059007	7.615906	-19.374609
H	12.805431	6.912140	-20.178975
C	11.838050	8.477216	-19.023618
H	11.101116	7.808888	-18.558840
H	12.125197	9.211794	-18.257492
C	11.226648	9.187292	-20.227679
H	12.003514	9.725094	-20.783876
H	10.787379	8.455146	-20.916913
C	10.182594	10.224559	-19.831437
H	10.649864	10.987463	-19.188116
H	9.817534	10.720946	-20.739818
N	9.053107	9.605925	-19.133630
H	9.150286	8.673641	-18.743526
C	7.958488	10.272598	-18.747880
N	7.813808	11.572737	-19.035982
H	8.454747	12.043411	-19.653779

H	6.928461	12.023510	-18.863689
N	7.006006	9.608078	-18.082623
H	7.291415	8.697443	-17.676069
H	6.284285	10.139967	-17.620329
C	14.229731	8.453437	-19.929210
O	14.532691	8.389747	-21.119471
N	14.804287	9.281965	-19.025956
H	14.477599	9.205211	-18.067498
C	16.027022	10.081610	-19.213184
H	16.002122	10.825774	-18.406965
C	16.141058	10.815824	-20.546559
H	17.010487	11.480281	-20.457416
H	16.367062	10.097975	-21.336950
C	14.921007	11.639080	-20.966164
H	15.146974	12.085202	-21.940904
H	14.054145	10.986759	-21.127515
C	14.528068	12.785385	-20.037952
H	15.425151	13.332185	-19.718273
H	13.876934	13.485784	-20.579818
N	13.832810	12.283482	-18.847692
H	13.397412	11.372947	-18.906761
C	13.634568	12.985679	-17.730935
N	13.995578	14.273073	-17.673474
H	14.140782	14.802631	-18.518754
H	13.839531	14.798246	-16.827348
N	13.082515	12.389820	-16.664519
H	13.128597	11.377441	-16.555891
H	12.950366	12.927221	-15.821190
C	17.263587	9.179255	-18.952607
O	17.997379	8.763087	-19.853600
N	17.421814	8.887939	-17.649053
H	16.758528	9.298276	-17.003532

C	18.362596	7.974846	-17.006758
H	17.743660	7.347242	-16.354103
C	19.368152	8.709655	-16.084996
H	20.297907	8.911139	-16.624874
H	18.937523	9.669107	-15.781380
C	19.583884	7.867225	-14.868585
N	19.196638	8.273206	-13.606925
H	18.917258	9.210226	-13.346804
C	19.222777	7.213983	-12.777943
H	18.962372	7.264692	-11.728900
N	19.614262	6.127765	-13.438174
C	19.860717	6.532774	-14.739973
H	20.179068	5.834872	-15.500332
C	19.086934	6.981672	-17.936104
O	20.310845	6.992792	-18.061126
N	18.297435	6.014513	-18.459305
H	17.283259	6.082477	-18.384523
C	18.833970	4.856853	-19.146166
H	19.919454	4.855341	-18.977139
C	18.236645	3.550627	-18.577267
H	17.144992	3.592928	-18.685197
H	18.591931	2.717149	-19.188035
C	18.594939	3.325232	-17.145636
N	19.446464	2.325056	-16.713586
H	19.894599	1.633731	-17.300861
C	19.575578	2.405044	-15.374760
H	20.187621	1.734449	-14.786520
N	18.843265	3.413204	-14.911436
C	18.230409	3.992068	-16.007682
H	17.594860	4.856868	-15.887005
C	18.658371	4.901844	-20.672133
O	18.815642	3.878313	-21.349024

N	18.385058	6.105648	-21.204865
H	18.304074	6.923861	-20.603172
C	18.216796	6.299132	-22.643482
H	17.784384	5.378250	-23.056522
C	17.262802	7.477462	-22.869259
H	16.378731	7.330437	-22.242685
H	17.748135	8.403482	-22.540261
C	16.797277	7.626143	-24.272517
N	17.608932	8.049428	-25.310563
H	18.601753	8.244224	-25.195210
C	16.911236	8.110631	-26.443473
H	17.286805	8.403473	-27.413230
N	15.663088	7.739618	-26.159856
H	14.909958	7.698288	-26.835207
C	15.561501	7.432625	-24.818865
H	14.634486	7.114166	-24.367795
C	19.594832	6.526973	-23.308440
O	19.920499	7.625420	-23.775155
N	20.387740	5.448459	-23.323648
Zn	18.835093	4.281195	-13.088985
H	10.440932	7.948353	-13.277743
H	11.094344	9.179614	-15.823819
H	11.738324	11.151173	-14.317483
H	11.365899	10.137238	-12.925451
H	9.586326	11.562525	-12.051291
H	8.320527	12.204210	-13.116844
H	9.995768	12.755077	-13.300140
H	8.993573	9.904329	-13.816423
H	9.902117	12.057336	-15.801934
H	8.226915	11.529721	-15.544469
H	9.403551	10.408177	-16.231698
H	21.328231	5.521286	-23.684362

H	20.080807	4.585236	-22.890704
C	9.837838	4.880660	-8.347615
H	10.161389	4.919305	-7.302870
H	9.544968	3.858268	-8.596742
H	8.971632	5.541120	-8.449506
O	20.256177	3.282411	-12.006586
H	21.127273	3.369792	-12.482631
O	22.534164	3.722125	-13.362578
H	22.570215	3.209549	-14.181026
H	20.094953	2.334487	-11.903430
H	22.499548	4.641175	-13.660522

$[ZnHL]^{2-}$ with {Glu-1, His-7, D-His-14, His-16} coordination

299

N	18.145798	2.999309	-9.778774
C	18.498337	2.024151	-8.703659
H	19.425825	1.522133	-8.996727
C	18.702061	2.768829	-7.367184
H	19.687559	3.245273	-7.417143
H	17.964850	3.579009	-7.309548
C	18.579219	1.931775	-6.084472
H	18.558898	0.854434	-6.304121
H	19.452474	2.075246	-5.440779
C	17.333120	2.234944	-5.221673
O	17.351756	1.837691	-4.036882
O	16.366054	2.870052	-5.769023
C	17.446907	0.912037	-8.643240
O	17.695715	-0.231864	-9.026419
N	16.240528	1.291250	-8.159810
H	16.233218	2.103369	-7.528697
C	15.209106	0.288777	-7.887848

H	15.540229	-0.443153	-7.141876
H	14.965877	-0.253313	-8.811151
C	13.966498	0.978378	-7.340750
O	13.466645	0.645389	-6.268147
N	13.470100	1.982143	-8.116675
H	14.006018	2.233372	-8.939942
C	12.662574	3.045934	-7.527079
H	12.441031	2.705580	-6.506224
C	13.469381	4.361316	-7.481919
H	14.528897	4.078298	-7.456062
H	13.310459	4.925521	-8.407423
C	13.203027	5.254459	-6.268580
H	13.926102	6.079321	-6.300440
H	12.203021	5.696987	-6.313197
C	13.375915	4.491436	-4.943438
H	12.445547	3.994302	-4.653169
H	14.130678	3.702723	-5.047895
N	13.744543	5.359591	-3.825657
H	12.997711	5.855486	-3.360583
C	14.992365	5.779139	-3.587263
N	16.035691	5.234162	-4.213515
H	15.977946	4.364747	-4.760945
H	16.961964	5.544684	-3.962709
N	15.188904	6.795732	-2.727040
H	14.412675	7.140551	-2.183923
H	16.106122	6.932288	-2.330583
C	11.283671	3.213106	-8.165326
O	10.628940	4.245482	-7.966511
N	10.810834	2.176049	-8.874478
H	11.387558	1.351672	-8.969247
C	9.407452	2.096016	-9.279914
H	9.343462	1.250785	-9.979562

C	8.506735	1.798610	-8.069361
H	9.030223	1.042392	-7.476238
H	8.437004	2.693218	-7.442117
C	7.100504	1.270369	-8.421610
H	6.939343	1.305911	-9.510044
H	6.994024	0.222021	-8.131852
C	5.952059	2.080591	-7.818378
O	4.941959	1.473397	-7.384496
O	6.074802	3.350888	-7.844897
C	9.061813	3.324293	-10.140159
O	9.798114	3.624494	-11.094526
N	7.939027	4.002815	-9.857442
H	7.343705	3.757612	-9.047737
C	7.480848	5.075356	-10.729676
H	7.545102	4.733029	-11.774530
C	6.024296	5.438231	-10.419071
H	5.918955	5.579812	-9.335698
H	5.802964	6.400851	-10.891784
C	5.046247	4.385179	-10.946782
H	5.002833	4.458355	-12.039471
H	5.399495	3.372484	-10.708983
C	3.634811	4.554675	-10.395838
H	3.319364	5.604883	-10.490032
H	2.931697	3.939154	-10.971924
N	3.612502	4.129089	-8.994281
H	4.509561	3.887462	-8.544281
C	2.507344	3.830549	-8.316926
N	2.625687	3.168551	-7.154168
H	3.457746	2.568582	-7.048403
H	1.786797	2.914497	-6.655292
N	1.300547	4.176976	-8.794595
H	1.225134	4.920475	-9.471959

H	0.478594	3.984954	-8.243455
C	8.360849	6.324577	-10.717278
O	8.207180	7.174574	-11.606090
N	9.312751	6.450318	-9.775985
H	9.494541	5.683838	-9.132350
C	10.326472	7.491264	-9.934544
H	9.855479	8.480236	-9.898168
C	11.357001	7.353237	-8.797937
H	10.908511	7.741518	-7.878185
H	11.579710	6.291744	-8.662877
C	12.662633	8.085163	-9.104445
O	12.792238	9.276202	-8.732124
O	13.542716	7.416852	-9.740131
C	10.997508	7.414582	-11.316539
O	11.343030	8.439994	-11.912321
N	11.224437	6.174199	-11.798942
H	10.807559	5.362861	-11.343254
C	11.915959	5.962175	-13.056441
H	12.749838	6.676173	-13.118838
C	12.469652	4.531834	-13.100446
H	11.711455	3.846387	-12.699519
H	12.640723	4.246216	-14.142209
C	13.766983	4.392090	-12.379078
N	14.151948	5.119485	-11.271949
H	13.638954	5.858865	-10.770742
C	15.417248	4.764339	-10.958862
H	15.966677	5.218365	-10.144012
N	15.866274	3.834321	-11.793880
C	14.837185	3.595993	-12.682297
H	14.925311	2.884921	-13.491334
C	11.090738	6.264178	-14.311268
O	11.667212	6.334334	-15.406713

N	9.776433	6.486953	-14.160746
H	9.361543	6.499834	-13.232466
C	8.959535	6.964272	-15.271742
H	9.054683	6.264847	-16.112740
C	7.492594	7.028146	-14.816462
H	7.267046	6.073382	-14.326593
H	7.390611	7.801927	-14.047366
C	6.492684	7.258058	-15.938899
H	6.622719	8.251044	-16.384945
H	6.661893	6.541068	-16.754180
C	5.015316	7.134099	-15.505295
O	4.163712	7.277577	-16.426834
O	4.763394	6.904777	-14.289943
C	9.440939	8.324483	-15.791354
O	9.148323	8.696918	-16.932910
N	10.157842	9.075086	-14.929320
H	10.334203	8.725131	-13.992804
C	10.647047	10.404284	-15.238193
C	10.566110	11.293155	-13.984405
C	9.181277	11.336896	-13.325148
C	8.094465	11.795651	-14.298628
C	9.238031	12.249548	-12.097702
C	12.091071	10.435679	-15.764654
O	12.589389	11.526187	-16.063414
N	12.755165	9.260681	-15.838129
H	12.276938	8.402801	-15.575071
C	14.195976	9.193003	-16.063604
H	14.556023	10.225935	-16.102668
C	14.911073	8.463376	-14.921266
H	14.541588	7.431715	-14.831298
H	15.977136	8.402182	-15.181214
C	14.735342	9.179151	-13.585549

H	15.030197	10.231764	-13.683954
H	13.681168	9.159587	-13.283320
C	15.567504	8.495586	-12.499646
H	15.324816	7.426301	-12.478183
H	16.638986	8.563312	-12.731538
N	15.295302	8.997907	-11.159806
H	14.630665	8.449109	-10.578463
C	15.701387	10.170534	-10.679011
N	16.558386	10.949772	-11.363558
H	17.100446	10.558628	-12.118120
H	16.954329	11.755783	-10.905603
N	15.237237	10.566235	-9.483843
H	14.378510	10.117507	-9.128751
H	15.465697	11.490709	-9.153066
C	14.542368	8.601630	-17.437288
O	14.955092	9.335381	-18.353657
N	14.425020	7.276936	-17.597120
H	14.051628	6.712076	-16.846414
C	14.735949	6.613481	-18.866574
H	14.671679	5.536838	-18.671441
C	13.718954	6.953776	-19.972906
H	13.777127	8.016433	-20.230419
H	13.975750	6.364331	-20.862216
C	12.325350	6.649089	-19.540397
N	11.853514	5.355756	-19.416670
C	10.615683	5.475920	-18.959458
H	9.935106	4.662127	-18.741067
N	10.273925	6.777779	-18.790349
H	9.428036	7.148794	-18.370460
C	11.359342	7.540496	-19.146889
H	11.352851	8.619463	-19.087312
C	16.218587	6.860647	-19.202768

O	17.102252	6.474685	-18.421375
N	16.498391	7.495463	-20.349900
H	15.737272	7.860875	-20.904949
C	17.855699	7.834299	-20.764958
H	18.446138	6.914808	-20.869377
C	17.768188	8.549870	-22.122727
H	17.231702	7.874938	-22.803335
H	17.155792	9.456115	-22.004904
C	19.120710	8.915845	-22.724308
H	19.642297	9.638359	-22.086888
H	19.759207	8.025131	-22.785476
C	18.999791	9.544412	-24.112978
H	18.400309	10.459578	-24.079214
H	19.993516	9.840369	-24.465174
N	18.450429	8.623592	-25.111419
H	19.067341	7.881928	-25.413771
C	17.202828	8.591627	-25.583791
N	16.288425	9.499861	-25.228054
H	16.446163	10.137992	-24.466462
H	15.339782	9.408083	-25.556640
N	16.853475	7.618258	-26.442163
H	17.570563	7.069257	-26.890741
H	15.970592	7.681650	-26.924817
C	18.628882	8.690845	-19.749151
O	19.856691	8.611408	-19.710406
N	17.910865	9.537211	-18.978546
H	16.897111	9.488304	-19.010773
C	18.517579	10.377777	-17.944197
H	17.677112	10.901154	-17.468112
C	19.505934	11.419493	-18.491448
H	19.767675	12.087441	-17.664127
H	20.424309	10.905956	-18.788749

C	19.007961	12.253040	-19.675300
H	19.860451	12.823855	-20.060895
H	18.680105	11.604305	-20.499669
C	17.912867	13.269379	-19.353986
H	18.177686	13.804985	-18.435010
H	17.837938	14.005005	-20.166461
N	16.619658	12.609647	-19.160212
H	16.408920	11.828201	-19.765457
C	15.652208	12.999727	-18.325257
N	15.751981	14.155903	-17.656482
H	16.353061	14.891682	-17.992788
H	15.010063	14.425859	-17.029135
N	14.583856	12.207920	-18.158702
H	14.702781	11.204209	-18.317698
H	13.906184	12.434431	-17.440385
C	19.169347	9.550697	-16.801765
O	20.090266	10.013392	-16.131298
N	18.558158	8.368098	-16.563914
H	17.884123	8.060676	-17.256575
C	19.020458	7.278154	-15.720227
H	18.144390	6.626301	-15.609278
C	19.469749	7.651927	-14.287008
H	20.450591	8.134180	-14.301032
H	18.748886	8.368652	-13.880831
C	19.485820	6.418308	-13.441600
N	20.592674	5.608959	-13.298722
H	21.504373	5.671090	-13.812221
C	20.217843	4.503001	-12.629212
H	20.898178	3.694186	-12.397676
N	18.927546	4.551358	-12.311016
C	18.462225	5.751164	-12.813620
H	17.428532	6.050527	-12.709489

C	20.121380	6.482728	-16.452332
O	21.014495	7.023403	-17.097179
N	19.990635	5.142733	-16.326493
H	19.236534	4.783498	-15.753465
C	20.759345	4.168100	-17.081225
H	21.257438	4.713996	-17.895033
C	21.827420	3.456997	-16.235437
H	21.356202	3.045343	-15.333671
H	22.210463	2.614915	-16.818267
C	22.965751	4.357251	-15.875553
N	22.908342	5.296962	-14.860322
C	24.074728	5.923425	-14.869015
H	24.376442	6.718974	-14.199704
N	24.878346	5.431637	-15.840280
H	25.821406	5.733960	-16.038857
C	24.190889	4.432371	-16.489876
H	24.620549	3.874015	-17.308816
C	19.769515	3.172417	-17.726974
O	19.992583	1.965946	-17.782410
N	18.659844	3.761418	-18.242934
H	18.522329	4.752902	-18.078093
C	17.464026	3.025239	-18.605478
H	17.759945	1.982715	-18.787370
C	16.417900	3.081523	-17.472026
H	16.095832	4.120404	-17.343128
H	15.541646	2.502143	-17.780999
C	16.923441	2.597910	-16.159776
N	17.229596	1.281599	-15.884687
H	17.196786	0.511862	-16.540194
C	17.597772	1.185870	-14.588917
H	17.886318	0.254599	-14.118941
N	17.546712	2.375662	-14.003818

C	17.135100	3.264397	-14.980607
H	16.977270	4.314618	-14.772165
C	16.853217	3.553333	-19.916240
O	15.634498	3.532027	-20.101362
N	17.732051	3.989384	-20.834926
Zn	17.735968	2.834361	-11.922294
H	10.023436	10.821404	-16.037841
H	10.863390	12.306694	-14.275778
H	11.305077	10.935798	-13.253136
H	8.345983	12.775997	-14.723237
H	7.132210	11.887386	-13.783353
H	7.958726	11.090770	-15.126683
H	8.925314	10.325305	-12.977152
H	9.468880	13.280602	-12.393561
H	10.009613	11.917589	-11.393454
H	8.276715	12.255127	-11.572918
H	18.894053	3.692946	-9.804292
H	17.336091	3.519796	-9.442859
H	17.391589	4.343414	-21.716539
H	18.719437	4.041335	-20.637544
O	19.454842	1.376952	-12.024982
H	19.971765	1.408902	-12.841430
O	16.473944	0.928978	-11.612987
H	15.906802	0.772695	-12.380568
H	15.847008	1.095795	-10.895500
H	20.113949	1.377916	-11.318205

$[ZnHL]^{2-}$ with {Glu-1, His-11, His-15, His-16} coordination

299

N	13.666732	0.681462	-18.229472
C	12.791150	0.991249	-17.052524

H	12.712316	2.081332	-16.980425
C	11.393572	0.389300	-17.196642
H	10.935760	0.802077	-18.102644
H	11.474631	-0.692298	-17.373859
C	10.509484	0.679321	-15.958551
H	11.120055	1.032432	-15.118054
H	9.789664	1.470509	-16.195262
C	9.748020	-0.586971	-15.542969
O	10.151289	-1.202186	-14.505824
O	8.797861	-0.949414	-16.281120
C	13.500817	0.547653	-15.767395
O	14.157061	1.345086	-15.082580
N	13.398061	-0.752513	-15.441554
H	12.830971	-1.372462	-16.000876
C	14.092314	-1.290790	-14.284123
H	13.954867	-2.377407	-14.283692
H	15.164020	-1.078714	-14.352378
C	13.640886	-0.742540	-12.918381
O	14.506967	-0.540254	-12.062285
N	12.310469	-0.606695	-12.770699
H	11.695996	-0.813653	-13.574649
C	11.557361	-0.107924	-11.618303
H	10.726474	-0.813422	-11.471132
C	12.338597	-0.040314	-10.302182
H	12.781624	-1.028347	-10.131753
H	13.165082	0.669085	-10.389368
C	11.456015	0.336749	-9.110599
H	11.262667	1.416855	-9.096773
H	10.480431	-0.165350	-9.169515
C	12.142224	-0.080528	-7.807598
H	12.215867	-1.173100	-7.765291
H	13.165544	0.304816	-7.768846

N	11.411507	0.347936	-6.618050
H	10.595519	-0.191924	-6.366683
C	11.631488	1.468071	-5.923694
N	12.633068	2.294888	-6.218678
H	13.251238	2.197156	-7.036218
H	12.766415	3.114069	-5.645878
N	10.851121	1.729569	-4.854920
H	10.004052	1.198544	-4.723501
H	10.859721	2.657329	-4.460512
C	10.956097	1.264380	-11.999335
O	11.635067	2.142430	-12.536956
N	9.640967	1.405814	-11.726547
H	9.159531	0.630623	-11.293699
C	8.822197	2.498593	-12.222578
H	9.293559	2.848183	-13.151860
C	7.403324	1.978609	-12.563960
H	7.204587	1.071012	-11.980320
H	6.650202	2.706785	-12.256162
C	7.239712	1.688738	-14.050288
H	7.994646	0.969773	-14.390517
H	6.268009	1.209663	-14.232055
C	7.306379	2.928077	-14.964337
O	7.376617	2.712014	-16.199871
O	7.264043	4.078224	-14.423363
C	8.805980	3.739012	-11.300496
O	9.463572	3.829105	-10.256071
N	8.021044	4.716274	-11.787699
H	7.606567	4.558128	-12.719634
C	7.715282	5.943286	-11.065139
H	7.099959	6.542262	-11.751990
C	6.891661	5.703839	-9.793142
H	7.490141	5.170427	-9.044557

H	6.617879	6.682038	-9.375926
C	5.612908	4.918828	-10.078765
H	5.060434	5.390305	-10.902940
H	5.862436	3.897733	-10.390112
C	4.724520	4.858146	-8.843950
H	5.275826	4.403001	-8.008686
H	4.428722	5.874767	-8.547709
N	3.531079	4.061207	-9.139621
H	3.439360	3.677103	-10.069499
C	2.557971	3.812248	-8.263887
N	1.481789	3.103949	-8.640438
H	1.493396	2.605099	-9.516709
H	0.833049	2.776687	-7.941391
N	2.642338	4.272561	-7.011464
H	3.359447	4.929337	-6.748879
H	1.859854	4.170724	-6.384312
C	8.986049	6.784304	-10.890579
O	9.716900	7.005304	-11.866602
N	9.251343	7.300942	-9.678969
H	8.668456	7.074111	-8.886266
C	10.390656	8.190453	-9.481762
H	10.312774	9.043664	-10.166225
C	10.403531	8.703668	-8.039673
H	9.390840	9.055300	-7.797625
H	10.648752	7.891937	-7.346930
C	11.346495	9.901370	-7.814851
O	11.364929	10.809497	-8.673247
O	12.008648	9.883660	-6.726720
C	11.717265	7.507219	-9.824159
O	12.670755	8.166458	-10.255561
N	11.810243	6.184389	-9.592073
H	10.980961	5.658969	-9.337136

C	13.015807	5.452191	-9.965487
H	13.883759	5.878747	-9.448483
C	12.823257	3.981095	-9.560566
H	12.472287	3.970811	-8.522516
H	12.020856	3.549228	-10.173855
C	14.056227	3.140225	-9.632617
N	14.464537	2.415001	-8.528230
C	15.542238	1.743505	-8.905681
H	16.123238	1.068934	-8.289898
N	15.847518	2.012113	-10.195941
H	16.614211	1.614904	-10.720201
C	14.910095	2.893088	-10.681139
H	14.936559	3.252351	-11.699102
C	13.294746	5.599221	-11.470472
O	14.422947	5.860264	-11.906114
N	12.235254	5.386175	-12.270026
H	11.317112	5.242375	-11.868924
C	12.345073	5.486298	-13.714540
H	13.181833	4.857145	-14.044612
C	11.054137	4.974812	-14.370004
H	10.871852	3.990827	-13.925595
H	10.217117	5.631147	-14.108603
C	11.161598	4.815411	-15.882772
H	12.048876	4.228221	-16.139053
H	10.294591	4.233438	-16.228357
C	11.141467	6.112384	-16.695347
O	11.822045	6.147748	-17.762369
O	10.404966	7.047083	-16.266967
C	12.700824	6.910337	-14.134349
O	13.539065	7.123703	-15.031620
N	12.095631	7.922719	-13.505891
H	11.395146	7.737234	-12.791540

C	12.421172	9.291154	-13.887805
C	11.576492	10.295910	-13.099257
C	10.070879	10.247377	-13.401868
C	9.767226	10.564716	-14.867296
C	9.343881	11.225308	-12.475332
C	13.913075	9.579235	-13.686161
O	14.555830	10.203493	-14.545641
N	14.457942	9.160264	-12.533480
H	13.888509	8.648356	-11.862713
C	15.849627	9.445047	-12.211622
H	16.036247	10.520618	-12.335310
C	16.152068	9.035864	-10.766385
H	15.864247	7.983350	-10.630615
H	17.236196	9.099603	-10.615219
C	15.430162	9.919823	-9.751119
H	15.780257	10.955195	-9.849220
H	14.351418	9.917111	-9.941487
C	15.660590	9.418115	-8.324658
H	15.408231	8.353712	-8.261555
H	16.716578	9.502266	-8.038608
N	14.812757	10.094789	-7.346313
H	13.861363	9.739157	-7.222958
C	14.975235	11.354599	-6.926907
N	16.109541	12.029920	-7.161146
H	16.931448	11.545545	-7.485650
H	16.259153	12.915091	-6.701654
N	13.972654	11.922631	-6.244131
H	13.062149	11.453599	-6.300232
H	14.022494	12.898030	-5.996847
C	16.837736	8.763811	-13.163968
O	17.918087	9.300858	-13.421105
N	16.479101	7.560608	-13.664726

H	15.606643	7.133735	-13.366699
C	17.381388	6.821264	-14.534287
H	18.404949	7.022206	-14.195955
C	17.141422	5.283981	-14.446664
H	18.113062	4.784744	-14.441376
H	16.677848	5.078918	-13.473007
C	16.348625	4.666872	-15.548231
N	15.115992	5.108358	-15.978374
H	14.544726	5.884899	-15.589812
C	14.715551	4.323219	-16.995096
H	13.778224	4.467206	-17.519420
N	15.626121	3.388783	-17.246633
C	16.655013	3.599334	-16.350205
H	17.545115	2.986258	-16.337522
C	17.342852	7.279107	-15.995240
O	18.137735	6.779400	-16.804402
N	16.416716	8.186431	-16.351704
H	15.758179	8.521249	-15.653915
C	16.089383	8.434782	-17.760034
H	16.340184	7.512956	-18.308119
C	14.605200	8.740412	-17.926242
H	14.032534	8.105100	-17.242561
H	14.417770	9.784695	-17.650510
C	14.175560	8.476792	-19.370906
H	14.953060	8.816695	-20.066575
H	14.071100	7.393540	-19.515132
C	12.889529	9.179152	-19.777175
H	13.016598	10.261208	-19.633103
H	12.699813	9.000253	-20.847235
N	11.762324	8.711483	-18.971967
H	11.818415	7.777962	-18.516027
C	10.566557	9.300894	-18.986705

N	10.359912	10.417890	-19.709982
H	10.978537	10.645530	-20.472181
H	9.423590	10.785756	-19.774934
N	9.574369	8.777015	-18.254493
H	9.838291	8.123903	-17.488636
H	8.714106	9.296908	-18.169261
C	16.957231	9.570278	-18.366756
O	16.473949	10.650479	-18.701429
N	18.267695	9.258844	-18.486521
H	18.546897	8.316055	-18.229773
C	19.200249	10.058148	-19.273839
H	18.694641	11.005752	-19.496844
C	20.512588	10.348762	-18.547841
H	20.971012	9.411516	-18.200578
H	21.195846	10.795674	-19.281748
C	20.429097	11.338560	-17.379007
H	21.453164	11.667348	-17.168097
H	19.879093	12.231777	-17.702810
C	19.850350	10.807541	-16.055374
H	20.006069	9.726744	-15.981231
H	20.386617	11.260358	-15.211280
N	18.412776	11.030289	-15.869061
H	17.839326	10.205638	-15.760151
C	17.894364	12.168348	-15.397324
N	18.651178	13.269583	-15.291388
H	19.553375	13.322951	-15.735058
H	18.219640	14.146601	-15.044116
N	16.606360	12.214112	-15.037601
H	16.069138	11.364798	-14.872940
H	16.238527	13.062235	-14.635405
C	19.333649	9.343015	-20.637260
O	18.331522	9.189731	-21.345032

N	20.543096	8.860631	-20.974084
H	21.332125	9.032364	-20.370150
C	20.803557	8.208999	-22.253420
H	21.852327	7.884097	-22.215056
C	20.646793	9.158541	-23.460207
H	20.996760	8.621555	-24.345644
H	19.586818	9.386974	-23.606061
C	21.419729	10.424243	-23.288121
N	20.894827	11.521044	-22.625754
C	21.850342	12.435946	-22.637153
H	21.788396	13.430373	-22.212645
N	22.961746	11.981647	-23.270835
H	23.816679	12.498216	-23.419574
C	22.703173	10.698412	-23.694697
H	23.431184	10.112599	-24.237330
C	19.970664	6.928050	-22.437822
O	19.695590	6.505295	-23.562479
N	19.608511	6.308299	-21.299374
H	19.924312	6.666013	-20.401410
C	18.791374	5.109835	-21.238344
H	18.288088	5.150588	-20.261023
C	19.596180	3.790546	-21.285466
H	19.765686	3.495636	-22.325160
H	20.569396	3.961828	-20.815622
C	18.853734	2.750300	-20.514119
N	19.354242	2.175211	-19.363227
H	20.305160	2.254141	-19.027898
C	18.365398	1.503997	-18.741993
H	18.491191	0.973644	-17.809761
N	17.233082	1.613402	-19.433689
C	17.538086	2.373806	-20.550649
H	16.796351	2.628751	-21.289349

C	17.667352	5.112702	-22.294597
O	17.523700	4.190160	-23.093777
N	16.828137	6.175352	-22.223270
H	16.925630	6.815294	-21.443620
C	15.666054	6.277686	-23.081959
H	15.782091	5.506504	-23.852891
C	14.348215	6.011799	-22.309677
H	14.207409	6.814847	-21.580641
H	13.520412	6.066345	-23.022536
C	14.337477	4.706385	-21.597739
N	13.869859	3.536333	-22.160256
H	13.469036	3.440580	-23.084019
C	14.011561	2.538505	-21.262717
H	13.721033	1.514306	-21.458377
N	14.550647	2.999830	-20.140431
C	14.764426	4.351530	-20.344832
H	15.216226	4.984667	-19.594103
C	15.545280	7.609099	-23.835063
O	14.582208	7.781283	-24.588156
N	16.507896	8.526051	-23.647531
Zn	15.387121	1.845591	-18.547530
H	12.255821	9.412510	-14.965798
H	11.953164	11.301454	-13.331585
H	11.742187	10.128695	-12.025382
H	9.673374	12.254055	-12.668695
H	9.544363	10.998988	-11.421733
H	8.261151	11.181429	-12.634654
H	8.685471	10.572778	-15.040349
H	10.202660	9.827407	-15.549049
H	10.161025	11.553533	-15.135481
H	9.700983	9.235815	-13.180632
H	13.097201	0.814992	-19.064273

H	13.893135	-0.312953	-18.224756
H	16.423264	9.411677	-24.125369
H	17.265638	8.418628	-22.984792
O	16.377882	0.527789	-16.793265
H	16.233550	-0.426838	-16.836135
O	20.433267	6.299404	-18.449624
H	21.181862	6.824608	-18.142360
H	15.935755	0.810605	-15.975617
H	19.770079	6.388529	-17.746882

$[ZnHL]^{2-}$ with {Glu-1, His-7, His-11, D-His-14} coordination

299

N	13.666732	0.681462	-18.229472
C	12.791150	0.991249	-17.052524
H	12.712316	2.081332	-16.980425
C	11.393572	0.389300	-17.196642
H	10.935760	0.802077	-18.102644
H	11.474631	-0.692298	-17.373859
C	10.509484	0.679321	-15.958551
H	11.120055	1.032432	-15.118054
H	9.789664	1.470509	-16.195262
C	9.748020	-0.586971	-15.542969
O	10.151289	-1.202186	-14.505824
O	8.797861	-0.949414	-16.281120
C	13.500817	0.547653	-15.767395
O	14.157061	1.345086	-15.082580
N	13.398061	-0.752513	-15.441554
H	12.830971	-1.372462	-16.000876
C	14.092314	-1.290790	-14.284123
H	13.954867	-2.377407	-14.283692
H	15.164020	-1.078714	-14.352378

C	13.640886	-0.742540	-12.918381
O	14.506967	-0.540254	-12.062285
N	12.310469	-0.606695	-12.770699
H	11.695996	-0.813653	-13.574649
C	11.557361	-0.107924	-11.618303
H	10.726474	-0.813422	-11.471132
C	12.338597	-0.040314	-10.302182
H	12.781624	-1.028347	-10.131753
H	13.165082	0.669085	-10.389368
C	11.456015	0.336749	-9.110599
H	11.262667	1.416855	-9.096773
H	10.480431	-0.165350	-9.169515
C	12.142224	-0.080528	-7.807598
H	12.215867	-1.173100	-7.765291
H	13.165544	0.304816	-7.768846
N	11.411507	0.347936	-6.618050
H	10.595519	-0.191924	-6.366683
C	11.631488	1.468071	-5.923694
N	12.633068	2.294888	-6.218678
H	13.251238	2.197156	-7.036218
H	12.766415	3.114069	-5.645878
N	10.851121	1.729569	-4.854920
H	10.004052	1.198544	-4.723501
H	10.859721	2.657329	-4.460512
C	10.956097	1.264380	-11.999335
O	11.635067	2.142430	-12.536956
N	9.640967	1.405814	-11.726547
H	9.159531	0.630623	-11.293699
C	8.822197	2.498593	-12.222578
H	9.293559	2.848183	-13.151860
C	7.403324	1.978609	-12.563960
H	7.204587	1.071012	-11.980320

H	6.650202	2.706785	-12.256162
C	7.239712	1.688738	-14.050288
H	7.994646	0.969773	-14.390517
H	6.268009	1.209663	-14.232055
C	7.306379	2.928077	-14.964337
O	7.376617	2.712014	-16.199871
O	7.264043	4.078224	-14.423363
C	8.805980	3.739012	-11.300496
O	9.463572	3.829105	-10.256071
N	8.021044	4.716274	-11.787699
H	7.606567	4.558128	-12.719634
C	7.715282	5.943286	-11.065139
H	7.099959	6.542262	-11.751990
C	6.891661	5.703839	-9.793142
H	7.490141	5.170427	-9.044557
H	6.617879	6.682038	-9.375926
C	5.612908	4.918828	-10.078765
H	5.060434	5.390305	-10.902940
H	5.862436	3.897733	-10.390112
C	4.724520	4.858146	-8.843950
H	5.275826	4.403001	-8.008686
H	4.428722	5.874767	-8.547709
N	3.531079	4.061207	-9.139621
H	3.439360	3.677103	-10.069499
C	2.557971	3.812248	-8.263887
N	1.481789	3.103949	-8.640438
H	1.493396	2.605099	-9.516709
H	0.833049	2.776687	-7.941391
N	2.642338	4.272561	-7.011464
H	3.359447	4.929337	-6.748879
H	1.859854	4.170724	-6.384312
C	8.986049	6.784304	-10.890579

O	9.716900	7.005304	-11.866602
N	9.251343	7.300942	-9.678969
H	8.668456	7.074111	-8.886266
C	10.390656	8.190453	-9.481762
H	10.312774	9.043664	-10.166225
C	10.403531	8.703668	-8.039673
H	9.390840	9.055300	-7.797625
H	10.648752	7.891937	-7.346930
C	11.346495	9.901370	-7.814851
O	11.364929	10.809497	-8.673247
O	12.008648	9.883660	-6.726720
C	11.717265	7.507219	-9.824159
O	12.670755	8.166458	-10.255561
N	11.810243	6.184389	-9.592073
H	10.980961	5.658969	-9.337136
C	13.015807	5.452191	-9.965487
H	13.883759	5.878747	-9.448483
C	12.823257	3.981095	-9.560566
H	12.472287	3.970811	-8.522516
H	12.020856	3.549228	-10.173855
C	14.056227	3.140225	-9.632617
N	14.464537	2.415001	-8.528230
C	15.542238	1.743505	-8.905681
H	16.123238	1.068934	-8.289898
N	15.847518	2.012113	-10.195941
H	16.614211	1.614904	-10.720201
C	14.910095	2.893088	-10.681139
H	14.936559	3.252351	-11.699102
C	13.294746	5.599221	-11.470472
O	14.422947	5.860264	-11.906114
N	12.235254	5.386175	-12.270026
H	11.317112	5.242375	-11.868924

C	12.345073	5.486298	-13.714540
H	13.181833	4.857145	-14.044612
C	11.054137	4.974812	-14.370004
H	10.871852	3.990827	-13.925595
H	10.217117	5.631147	-14.108603
C	11.161598	4.815411	-15.882772
H	12.048876	4.228221	-16.139053
H	10.294591	4.233438	-16.228357
C	11.141467	6.112384	-16.695347
O	11.822045	6.147748	-17.762369
O	10.404966	7.047083	-16.266967
C	12.700824	6.910337	-14.134349
O	13.539065	7.123703	-15.031620
N	12.095631	7.922719	-13.505891
H	11.395146	7.737234	-12.791540
C	12.421172	9.291154	-13.887805
C	11.576492	10.295910	-13.099257
C	10.070879	10.247377	-13.401868
C	9.767226	10.564716	-14.867296
C	9.343881	11.225308	-12.475332
C	13.913075	9.579235	-13.686161
O	14.555830	10.203493	-14.545641
N	14.457942	9.160264	-12.533480
H	13.888509	8.648356	-11.862713
C	15.849627	9.445047	-12.211622
H	16.036247	10.520618	-12.335310
C	16.152068	9.035864	-10.766385
H	15.864247	7.983350	-10.630615
H	17.236196	9.099603	-10.615219
C	15.430162	9.919823	-9.751119
H	15.780257	10.955195	-9.849220
H	14.351418	9.917111	-9.941487

C	15.660590	9.418115	-8.324658
H	15.408231	8.353712	-8.261555
H	16.716578	9.502266	-8.038608
N	14.812757	10.094789	-7.346313
H	13.861363	9.739157	-7.222958
C	14.975235	11.354599	-6.926907
N	16.109541	12.029920	-7.161146
H	16.931448	11.545545	-7.485650
H	16.259153	12.915091	-6.701654
N	13.972654	11.922631	-6.244131
H	13.062149	11.453599	-6.300232
H	14.022494	12.898030	-5.996847
C	16.837736	8.763811	-13.163968
O	17.918087	9.300858	-13.421105
N	16.479101	7.560608	-13.664726
H	15.606643	7.133735	-13.366699
C	17.381388	6.821264	-14.534287
H	18.404949	7.022206	-14.195955
C	17.141422	5.283981	-14.446664
H	18.113062	4.784744	-14.441376
H	16.677848	5.078918	-13.473007
C	16.348625	4.666872	-15.548231
N	15.115992	5.108358	-15.978374
H	14.544726	5.884899	-15.589812
C	14.715551	4.323219	-16.995096
H	13.778224	4.467206	-17.519420
N	15.626121	3.388783	-17.246633
C	16.655013	3.599334	-16.350205
H	17.545115	2.986258	-16.337522
C	17.342852	7.279107	-15.995240
O	18.137735	6.779400	-16.804402
N	16.416716	8.186431	-16.351704

H	15.758179	8.521249	-15.653915
C	16.089383	8.434782	-17.760034
H	16.340184	7.512956	-18.308119
C	14.605200	8.740412	-17.926242
H	14.032534	8.105100	-17.242561
H	14.417770	9.784695	-17.650510
C	14.175560	8.476792	-19.370906
H	14.953060	8.816695	-20.066575
H	14.071100	7.393540	-19.515132
C	12.889529	9.179152	-19.777175
H	13.016598	10.261208	-19.633103
H	12.699813	9.000253	-20.847235
N	11.762324	8.711483	-18.971967
H	11.818415	7.777962	-18.516027
C	10.566557	9.300894	-18.986705
N	10.359912	10.417890	-19.709982
H	10.978537	10.645530	-20.472181
H	9.423590	10.785756	-19.774934
N	9.574369	8.777015	-18.254493
H	9.838291	8.123903	-17.488636
H	8.714106	9.296908	-18.169261
C	16.957231	9.570278	-18.366756
O	16.473949	10.650479	-18.701429
N	18.267695	9.258844	-18.486521
H	18.546897	8.316055	-18.229773
C	19.200249	10.058148	-19.273839
H	18.694641	11.005752	-19.496844
C	20.512588	10.348762	-18.547841
H	20.971012	9.411516	-18.200578
H	21.195846	10.795674	-19.281748
C	20.429097	11.338560	-17.379007
H	21.453164	11.667348	-17.168097

H	19.879093	12.231777	-17.702810
C	19.850350	10.807541	-16.055374
H	20.006069	9.726744	-15.981231
H	20.386617	11.260358	-15.211280
N	18.412776	11.030289	-15.869061
H	17.839326	10.205638	-15.760151
C	17.894364	12.168348	-15.397324
N	18.651178	13.269583	-15.291388
H	19.553375	13.322951	-15.735058
H	18.219640	14.146601	-15.044116
N	16.606360	12.214112	-15.037601
H	16.069138	11.364798	-14.872940
H	16.238527	13.062235	-14.635405
C	19.333649	9.343015	-20.637260
O	18.331522	9.189731	-21.345032
N	20.543096	8.860631	-20.974084
H	21.332125	9.032364	-20.370150
C	20.803557	8.208999	-22.253420
H	21.852327	7.884097	-22.215056
C	20.646793	9.158541	-23.460207
H	20.996760	8.621555	-24.345644
H	19.586818	9.386974	-23.606061
C	21.419729	10.424243	-23.288121
N	20.894827	11.521044	-22.625754
C	21.850342	12.435946	-22.637153
H	21.788396	13.430373	-22.212645
N	22.961746	11.981647	-23.270835
H	23.816679	12.498216	-23.419574
C	22.703173	10.698412	-23.694697
H	23.431184	10.112599	-24.237330
C	19.970664	6.928050	-22.437822
O	19.695590	6.505295	-23.562479

N	19.608511	6.308299	-21.299374
H	19.924312	6.666013	-20.401410
C	18.791374	5.109835	-21.238344
H	18.288088	5.150588	-20.261023
C	19.596180	3.790546	-21.285466
H	19.765686	3.495636	-22.325160
H	20.569396	3.961828	-20.815622
C	18.853734	2.750300	-20.514119
N	19.354242	2.175211	-19.363227
H	20.305160	2.254141	-19.027898
C	18.365398	1.503997	-18.741993
H	18.491191	0.973644	-17.809761
N	17.233082	1.613402	-19.433689
C	17.538086	2.373806	-20.550649
H	16.796351	2.628751	-21.289349
C	17.667352	5.112702	-22.294597
O	17.523700	4.190160	-23.093777
N	16.828137	6.175352	-22.223270
H	16.925630	6.815294	-21.443620
C	15.666054	6.277686	-23.081959
H	15.782091	5.506504	-23.852891
C	14.348215	6.011799	-22.309677
H	14.207409	6.814847	-21.580641
H	13.520412	6.066345	-23.022536
C	14.337477	4.706385	-21.597739
N	13.869859	3.536333	-22.160256
H	13.469036	3.440580	-23.084019
C	14.011561	2.538505	-21.262717
H	13.721033	1.514306	-21.458377
N	14.550647	2.999830	-20.140431
C	14.764426	4.351530	-20.344832
H	15.216226	4.984667	-19.594103

C	15.545280	7.609099	-23.835063
O	14.582208	7.781283	-24.588156
N	16.507896	8.526051	-23.647531
Zn	15.387121	1.845591	-18.547530
H	12.255821	9.412510	-14.965798
H	11.953164	11.301454	-13.331585
H	11.742187	10.128695	-12.025382
H	9.673374	12.254055	-12.668695
H	9.544363	10.998988	-11.421733
H	8.261151	11.181429	-12.634654
H	8.685471	10.572778	-15.040349
H	10.202660	9.827407	-15.549049
H	10.161025	11.553533	-15.135481
H	9.700983	9.235815	-13.180632
H	13.097201	0.814992	-19.064273
H	13.893135	-0.312953	-18.224756
H	16.423264	9.411677	-24.125369
H	17.265638	8.418628	-22.984792
O	16.377882	0.527789	-16.793265
H	16.233550	-0.426838	-16.836135
O	20.433267	6.299404	-18.449624
H	21.181862	6.824608	-18.142360
H	15.935755	0.810605	-15.975617
H	19.770079	6.388529	-17.746882

Reference:

1 K. Szarszoń, S. Andrä, T. Janek and J. Wątły, *Inorg Chem*, 2024, 63, 11616–11627.
